# Evaluation of Interventions for Cognitive Symptoms in Long COVID: A Randomized Clinical Trial

**DOI:** 10.1001/jamaneurol.2025.4415

**Published:** 2025-11-10

**Authors:** David S. Knopman, Deborah Koltai, Daniel T. Laskowitz, Jacqueline Becker, Leigh Charvet, Juan Wisnivesky, Alex Federman, Adam Silverstein, Yuliya Lokhnygina, Giuseppina Pilloni, Michelle Haddad, Henry Mahncke, Tom Van Vleet, Rong Huang, Wendy Cox, Diana Terry, Jeannie Karwowski, Netia McCray, Jenny J. Lin, Grace A. McComsey, Upinder Singh, Linda N. Geng, Helen Y. Chu, Rebecca Reece, James Moy, Zoe Arvanitakis, Sairam Parthasarathy, Thomas F. Patterson, Aditi Gupta, Luis Ostrosky-Zeichner, Jeffrey Parsonnet, Elaine T. Kiriakopoulos, Tamara G. Fong, Janet Mullington, Sarah Jolley, Nirav S. Shah, Sarah Shizuko Morimoto, Joyce K. Lee-Iannotti, William D. S. Killgore, Brigid Dwyer, William Stringer, Carmen Isache, Jennifer A. Frontera, Jerry A. Krishnan, Ashley O’Steen, Melissa James, Barrie L. Harper, Kanecia O. Zimmerman

**Affiliations:** 1 Department of Neurology, Mayo Clinic, Rochester, Minnesota; 2 Department of Neurology, Duke University School of Medicine, Durham, North Carolina; 3 Division of General Internal Medicine, Icahn School of Medicine at Mount Sinai, New York, New York; 4 Department of Neurology, Grossman School of Medicine, New York University, New York; 5 Duke Clinical Research Institute, Duke University School of Medicine, Durham, North Carolina; 6 Department of Biostatistics and Bioinformatics, Duke University School of Medicine, Durham, North Carolina; 7 Department of Rehabilitation Medicine, Emory University School of Medicine, Atlanta, Georgia; 8 Posit Science, San Francisco, California; 9 RECOVER Patient, Caregiver, or Community Representative, Durham, North Carolina; 10 Department of Medicine, Icahn School of Medicine at Mount Sinai, New York, New York; 11 Department of Medicine and Pediatrics, Case Western Reserve University and University Hospitals Cleveland Medical Center, Cleveland, Ohio; 12 Department of Internal Medicine, University of Iowa, Iowa City; 13 Department of Medicine, Stanford University School of Medicine, Stanford, California; 14 Department of Medicine, University of Washington, Seattle; 15 Department of Medicine, West Virginia University School of Medicine, Morgantown; 16 Department of Internal Medicine, Rush University Medical Center, Chicago, Illinois; 17 Department of Neurological Sciences, Rush Alzheimer’s Disease Center, Rush University Medical Center, Chicago, Illinois; 18 Department of Medicine, University of Arizona, Tucson; 19 Department of Medicine/Infectious Diseases, University of Texas Health San Antonio, San Antonio; 20 Departments of Internal Medicine and Neurology, University of Kansas Medical Center, Kansas City; 21 Division of Infectious Diseases, University of Texas Health Science Center at Houston, Houston; 22 Department of Medicine, Dartmouth-Hitchcock Medical Center, Lebanon, New Hampshire; 23 Department of Neurology, Dartmouth Health, Geisel School of Medicine, Dartmouth College, Hanover, New Hampshire; 24 Department of Neurology, Beth Israel Deaconess Medical Center and Harvard School of Medicine, Boston, Massachusetts; 25 Division of Pulmonary and Critical Care Medicine, University of Colorado Anschutz Medical Center, Aurora; 26 Division of Infectious Diseases, Endeavor Health, Evanston, Illinois; 27 Population Health Sciences, Division of Health Systems Innovation and Research, University of Utah School of Medicine, Salt Lake City; 28 Department of Neurology, Barrow Neurological Institute, University of Arizona College of Medicine—Phoenix, Phoenix; 29 Department of Psychiatry, University of Arizona, Tucson; 30 Chobanian & Avedisian School of Medicine at Boston University, Boston, Massachusetts; 31 Department of Medicine, Lundquist Institute for Biomedical Innovation at Harbor-UCLA Medical Center, Torrance, California; 32 Department of Medicine, University of Florida-Jacksonville, Jacksonville; 33 Department of Medicine, Illinois Research Network and University of Illinois Chicago, Chicago; 34 Department of Pediatrics, Duke University School of Medicine, Durham, North Carolina

## Abstract

**Importance:**

Treatment for cognitive dysfunction due to postacute sequelae of long COVID (ie, symptoms of fatigue, malaise, weakness, confusion that persist beyond 12 weeks after an initial COVID infection) remains a significant unmet need.

**Objective:**

To test evidence-based rehabilitation strategies for improving cognitive symptoms in persons with long COVID.

**Design, Setting, and Participants:**

This was a 5-arm, multicenter, randomized clinical trial of 3 remotely delivered interventions conducted between August 17, 2023, and June 10, 2024. The study took place at 22 trial sites and included the screening of individuals with cognitive long COVID.

**Interventions:**

Participants were randomized to 1 of 5 arms: adaptive computerized cognitive training (BrainHQ [Posit Science]), cognitive-behavioral rehabilitation involving both group and individual counseling sessions (PASC-Cognitive Recovery [PASC-CoRE]) paired with BrainHQ, and transcranial direct current stimulation (tDCS) paired with BrainHQ. Two comparator arms were included as follows: unstructured computer puzzles and games (active comparator) and sham tDCS paired with BrainHQ. The interventions occurred 5 times per week over 10 weeks.

**Main Outcomes and Measures:**

Cognitive and behavioral in-person assessments were performed at baseline, midintervention, at the end of intervention, and 3 months after the end of the intervention. The primary outcome measure was the modified Everyday Cognition Scale 2 (ECog2) completed at the end of the intervention compared to the baseline visit based on participant self-report looking back over the prior 7 days.

**Results:**

A total of 378 individuals were screened, from which there were 328 participants (median [IQR] age, 48.0 [37.0-58.0] years; 241 female [73.5%]; race: 15 Asian [4.6%], 47 Black [14.3%], and 235 White [71.6%]; ethnicity: 52 Hispanic [15.9%]). None of the 3 active interventions demonstrated benefits on the modified ECog2 in the intention-to-treat population by the end of the intervention period. The adjusted differences in mean change were 0.0 (95% CI, −0.2 to 0.2) for BrainHQ vs active comparator, 0.1 (95% CI, −0.1 to 0.3) for PASC-CoRE + BrainHQ vs active comparator, 0.0 (95% CI, −0.2 to 0.2) for tDCS-active + BrainHQ vs tDCS-sham + BrainHQ, and 0.1 (95% CI, −0.1 to 0.3) for PASC-CoRE + BrainHQ vs BrainHQ alone. Secondary participant-reported outcomes and neuropsychological tests showed no differential benefits for any treatment arm. All 5 arms demonstrated some improvements over time on the modified ECog2 and on secondary outcomes. There were no serious adverse events attributable to the interventions.

**Conclusions and Relevance:**

This phase 2 randomized clinical trial failed to demonstrate differential benefits for online cognitive training, a structured cognitive rehabilitation program, and tDCS for cognitive long COVID.

**Trial Registration:**

ClinicalTrials.gov Identifier: NCT05965739.

## Key Points

**Question:**

Do evidence-based rehabilitation strategies improve cognitive symptoms in persons with long COVID (ie, symptoms of fatigue, malaise, weakness, confusion that persist beyond 12 weeks after an initial COVID infection)?

**Findings:**

This randomized clinical trial included 328 participants randomly assigned to 3 active interventions over 10 weeks at 22 clinical sites. None of the interventions demonstrated benefits on the modified Everyday Cognition Scale 2 in the intention-to-treat population by the end of the intervention period.

**Meaning:**

The trial failed to demonstrate differential benefits from online cognitive training, a structured cognitive rehabilitation program, or transcranial direct current stimulation in participants with cognitive long COVID.

## Teaser

This randomized clinical trial investigates if evidence-based rehabilitation strategies improve cognitive symptoms in persons with long COVID.

## Introduction

Long COVID, the postacute sequelae of SARS-CoV-2 infection (PASC), is estimated to impact 7% of US adults.^1^ Cognitive symptoms with long COVID include executive dysfunction, memory difficulties, and reduced mental agility, which may substantially interfere with employment and well-being in daily life,^2,3^ Symptoms of cognitive dysfunction may persist for several years.^4^

The Researching COVID to Enhance Recovery (RECOVER) project, conducted under the auspices of the National Institutes of Health, created work groups to conduct clinical trials for different manifestations of long COVID. The RECOVER-NEURO workgroup, focused on cognition, included clinical trialists and patient representatives. To test the hypothesis that cognitive long COVID would be responsive to rehabilitative strategies, the work group designed a multiarm trial to test 3 rehabilitation approaches in parallel, all of which could be administered remotely: (1) BrainHQ (Posit Science)—an online program with demonstrated benefits in improving cognitive efficiency by continuously adapting the training to individual performance^5^ and has been reported to improve cognition in persons with mild cognitive impairment due to neurodegenerative disease^5^ and multiple sclerosis^6^; (2) PASC-Cognitive Recovery (PASC-CoRE)—a cognitive rehabilitation intervention specifically adapted for persons with long COVID using evidence-based rehabilitation methodologies^7^ with demonstrated efficacy in improving cognition in other populations (eg, mild traumatic brain injury); and (3) transcranial direct current stimulation (tDCS), a noninvasive, remotely administered approach to brain stimulation intended to improve cognitive function.^8^

## Methods

### Study Design and Oversight

**Figure 1.**
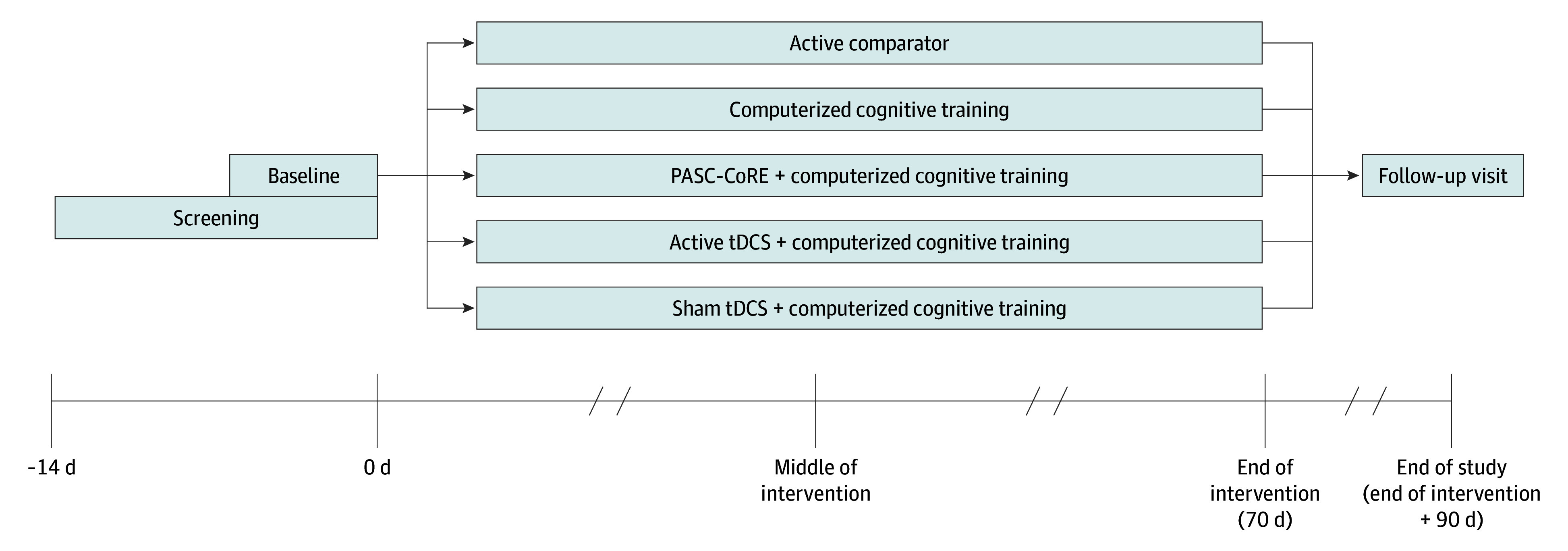
Study Design PASC indicates postacute sequelae of SARS-CoV-2 infection; PASC-CoRE, PASC-Cognitive Recovery; tDCS, transcranial direct current stimulation.

A detailed description of A Platform Protocol for Evaluation of Interventions for Cognitive Dysfunction in Postacute Sequelae of SARS-CoV-2 Infection (RECOVER-NEURO) study design has been published.^9^ The RECOVER-NEURO trial was approved by a central institutional review board and by each participating institution. The study protocol and statistical analysis plan are available in Supplement 1. The RECOVER-NEURO trial was designed as a 5-arm multicenter, phase 2, randomized clinical trial to treat the cognitive symptoms of long COVID, as depicted in Figure 1. Participants were recruited and enrolled at 22 trial sites in the US. Multiple strategies were used to encourage rapid and diverse enrollment. Ability to recruit from a diverse population was prioritized during site selection. All sites received recruitment flyers (English and Spanish) and email, letter, or call scripts. Sites were encouraged to prescreen. Participant self-referral was also possible through the RECOVER website.^10^ Within 2 weeks after a screening visit to determine eligibility for enrollment, participants were randomized and baseline assessments were administered. Interventions were initiated within 3 weeks and were 10 weeks in duration. Efficacy assessments occurred midway through the intervention at day 35 ± 3 days and at the end of the intervention (EOI) at day 70 ± 3. A follow-up evaluation at the end of the study (EOS) took place 3 months (day 160 ± 3) after the completion of the intervention to assess durability of any beneficial effects. This study followed the Consolidated Standards of Reporting Trials (CONSORT) reporting guidelines.

All intervention arms were delivered as contactless, telehealth-based treatments. The neuromodulation research team at NYU Langone Health (New York, New York) centrally coordinated intervention delivery for all participants across sites, providing each participant with a study kit that included a preprogrammed iPad (Apple Inc) for video visits, their assigned training module (BrainHQ or active comparator), and a tDCS device and headset when applicable. The team administered all 5 treatment arms via video visits, including remotely supervised tDCS, BrainHQ, and active comparator sessions. For participants assigned to the PASC-CoRE arm, the telehealth component was delivered by providers at the Icahn School of Medicine at Mount Sinai (New York, New York) and Emory University (Atlanta, Georgia).

### Study Participants

All participants were 18 years or older. Participants self-identified the following races: Asian, Black or African American, White, or other, which included American Indian or Alaska Native, multiracial, Native Hawaiian or Pacific Islander, other, unknown, and not reported. Participants also self-identified the following ethnicities: Hispanic, Latino, or Spanish. Because long COVID had disproportionate effects on Asian, Black or African American, American Indian or Alaska Native, multiracial, and Native Hawaiian or Pacific Islander individuals and those in the Hispanic community, extra resources were devoted to recruiting participants from those communities. Inclusion and exclusion criteria are outlined in eTable 1 in Supplement 2. The key symptom-specific inclusion criterion for enrollment was self-rated cognitive complaints after a SARS-CoV-2 infection that had been present for at least 12 weeks and was operationalized by a T score of less than 40 on the Patient-Reported Outcomes Measurement Information System (PROMIS) Cognitive Function Short Form 8a assessment. Because there is no established association between cognitive complaints and objective cognitive testing for diagnosis or prognosis in long COVID, low scores on objective cognitive tests were not required for participation.

Persons with cognitive disorders that preceded the index infection, or who had preexisting neuropsychiatric or medical conditions that would complicate assessment, were excluded, as were individuals on psychoactive medications that had been prescribed to treat the cognitive symptoms of long COVID. All participants provided written informed consent.

### Interventions

The RECOVER-NEURO trial tested 3 active interventions within the 5-arm trial. The study arms were (1) active comparator (video puzzles and games), the control condition for all 3 interventions; (2) BrainHQ; (3) PASC-CoRE + BrainHQ; (4) tDCS-active + BrainHQ; and (5) tDCS-sham + BrainHQ, the specific control arm for the tDCS-active arm.

The active comparator consisted of a set of 8 online cognitive puzzles and games provided by Posit Science and selected to have face validity with the BrainHQ exercises. While engaging, these exercises did not include the adaptive algorithm that drives continuous challenge for optimal learning. The activities were designed to be an active comparator to the structured cognitive training of BrainHQ. Rather than using a wait-list control, an active comparator matched participant engagement with the active treatments. Wait-list control conditions and no-intervention conditions were rejected because of their undesirability for participants and concerns about gross differences in engagement compared with the active interventions. We reasoned that if the interventions were to be clinically meaningful, the added expense and effort they required ought to be distinguishable from something easily or freely available such as computer games.

BrainHQ is an interactive, online, computer-based cognitive training program developed by Posit Science to improve cognitive function based on neuroplasticity principles. It targets memory, attention, and processing speed, spanning up to 29 training exercises for a total of 1160 possible training levels, emphasizing speed and accuracy with a built-in reward system. An adaptive algorithm individualizes training by adjusting to each user’s baseline performance learning rate, using visually appealing exercises that maintain user engagement.^5,6^ Both BrainHQ and the active comparator were administered for 30 minutes, 5 times per week, over the 10-week intervention.

PASC-CoRE is a manualized cognitive rehabilitation intervention that was specifically adapted for persons with long COVID and uses an evidence-based protocol that has demonstrated efficacy in improving attention and executive functions in other populations.^7^ PASC-CoRE consisted of 9, 90-minute group sessions and 3, 30-minute individual sessions. PASC-CoRE comprises 3 essential training components: (1) attention regulation and mindfulness, (2) goal management, and (3) fatigue management strategies (eg, pacing). Attention regulation training is grounded in mindfulness-based approaches to emphasize redirection of cognitive processes toward salient tasks by improving self-monitoring and reducing cognitive load. Goal management training encourages participants to leverage metacognitive strategies to identify, select, and execute personally relevant and functional goals. Fatigue management training teaches strategies for understanding, monitoring, and managing physical and cognitive fatigue pacing, planning, and avoiding overstimulation. Participants were instructed to use these principles in various personally relevant situations. All sessions were virtual and conducted through both individual and group sessions. Additionally, homework was assigned for more practice in applying these principles.

The tDCS intervention was administered with the goal of improving cognitive functioning by enhancing synaptic plasticity and network efficiency.^8^ Participants received a tDCS device (Soterix Medical Inc) specifically designed for home-based use (Soterix 1 × 1 mini-CT). For each stimulation session, the device delivered a low-intensity electrical current of 2.0 mA for 30 minutes through 2 sponge electrodes placed on the scalp, targeting the dorsolateral prefrontal cortex. The stimulation was administered simultaneously with performance of BrainHQ. According to the 10-20 electroencephalography system, the anode was positioned over F3 (left frontal lobe) and the cathode over F4 (right frontal lobe) using a standardized and validated headset.^11^ The electrodes were inserted in a presaturated saline sponge (single use for each session) and attached to a headset by snapping into place. Each tDCS device was preprogrammed to deliver either a constant direct current of 2.0 mA for 30 minutes (tDCS-active arm) or a placebo condition (tDCS-sham), consisting of a ramp up and ramp down to 2.0 mA during the first and last 60 seconds. The benefits of tDCS appear to be dependent on the underlying brain state.^12,13^ When applied to regions actively engaged during cognitive training, tDCS has been shown to enhance learning and cognitive performance,^14,15^ particularly when stimulation targets the dorsolateral prefrontal cortex, a region associated with working memory and processing speed. Both active and sham tDCS sessions plus BrainHQ were administered in 5, 30-minute sessions weekly.

### Outcomes

The primary outcome measure was the modified Everyday Cognition Scale 2 (ECog2),^16^ a 41-item participant-reported measure reflecting perceived cognitive and functional decline across multiple cognitive domains and functional skills. The modified ECog2 was slightly modified from its original form for the population with long COVID by changing the temporal reference anchor to the past 7 days (eTable 2 in Supplement 2 for the modified ECog2 instrument.) The score on the modified ECog2 represented the average rating (on a 1-5 scale from no difficulty to great difficulty) of all completed items.

Components of the participant self-report PROMIS^17^ instrument that included cognitive functioning, general health status, fatigue symptoms, depression, anxiety, and sleep-related impairments in the prior 7 days were administered as secondary outcomes.

Patient-reported outcomes including the primary outcome measure (modified ECog2) were administered in person at baseline, end of intervention, and end of study. These assessments were completed remotely at the middle of intervention time point.

Neuropsychological testing (secondary outcomes) was administered in person and included the Auditory Verbal Learning Test (World Health Organization–University of California Los Angeles version)^18^ at baseline and EOS, and an alternative version^19^ at EOI; the Symbol Digit Modalities Test,^20^ the Digit Vigilance Test,^21^ the National Institutes of Health Toolbox Flanker Test,^22^ and lexical and semantic fluency. The computer-based Cogstate Brief Battery measuring reaction times and working memory was administered to a subset of participants; these outcomes will be reported in a future analysis.

### Sample Size Considerations

No published data were available to evaluate the minimal clinically meaningful effect size in the long COVID population. To increase the likelihood that the RECOVER-NEURO trial would detect an effect of practical benefit, we targeted an effect size of greater than 0.5 and derived a sample size based on that goal. A sample size of 50 participants per group provided 90% power to detect an effect size of 0.655 for comparing any 2 arms, using a 2-sample *t* test and assuming 1:1 randomization and a 2-sided type I error rate of 0.05. Assuming 20% loss to follow-up, 63 participants per arm were randomized.

Participants were randomly assigned to 1 of the 5 treatment arms^9^ (Supplement 1). All evaluators were blinded to treatment assignment. Participants assigned to PASC-CoRE were aware of their treatment. Participants assigned to receive tDCS treatment were blinded to active or sham stimulations. Percent adherence for each component of an intervention was calculated by dividing the number of completed sessions by the number of expected sessions and then multiplying by 100.

### Safety Assessments

All reported adverse events were tabulated. The Modified DePaul Symptom Questionnaire—Post-Exertional Malaise (DSQ-PEM)^23^ is a self-reported questionnaire administered to monitor postexertional malaise because of concerns about the tolerability of study procedures. The DSQ-PEM assessed symptom frequency and severity over a look-back period of since the previous visit when administered the day after baseline, at middle of intervention, at EOI, and at EOS to assess symptoms following in-person assessments. The DSQ-PEM was also administered twice weekly during the trial to assess symptoms related to the interventions, where the look-back period was the past 7 days. The DSQ-PEM was previously validated in patients with myalgic encephalomyelitis and chronic fatigue syndrome.^23^

### Statistical Analysis

The primary analysis was the change from baseline to EOI on scores from the modified ECog2 in the intention-to-treat (ITT) population. Categorical data are presented as No. (%), and continuous data are presented as either median (IQR) or mean (95% CI). All *P *values are 2-sided, and α was prespecified at .05. Further details on the analytic plan can be found in Supplement 1. Analyses were adjusted for baseline modified ECog2 score, age, sex, years of education, and baseline psychological distress (a metric combining PROMIS depression and anxiety domains). The primary pairwise comparisons were the following:

- BrainHQ vs active comparator
- PASC-CoRE + BrainHQ vs active comparator
- tDCS-active + BrainHQ vs tDCS-sham + BrainHQ
- PASC-CoRE + BrainHQ vs BrainHQ

The secondary pairwise comparisons were as follows:

- tDCS-active + BrainHQ vs active comparator
- tDCS-active + BrainHQ vs BrainHQ

The primary end point was also analyzed in the modified ITT population, defined as all participants who received at least 1 intervention session, and in the per-protocol populations (≥75% treatment compliance; no major protocol deviations). Secondary and exploratory efficacy end points (which included modified ECog2 change from baseline to EOS) were conducted on the ITT population. A tipping point analysis was performed^24^ using multiple imputation to determine the impact of missing data across a range of assumptions. Safety analyses were conducted on the modified ITT population. Data were analyzed using SAS, version 9.4 (SAS Institute).

## Results

### Baseline Characteristics

**Figure 2.**
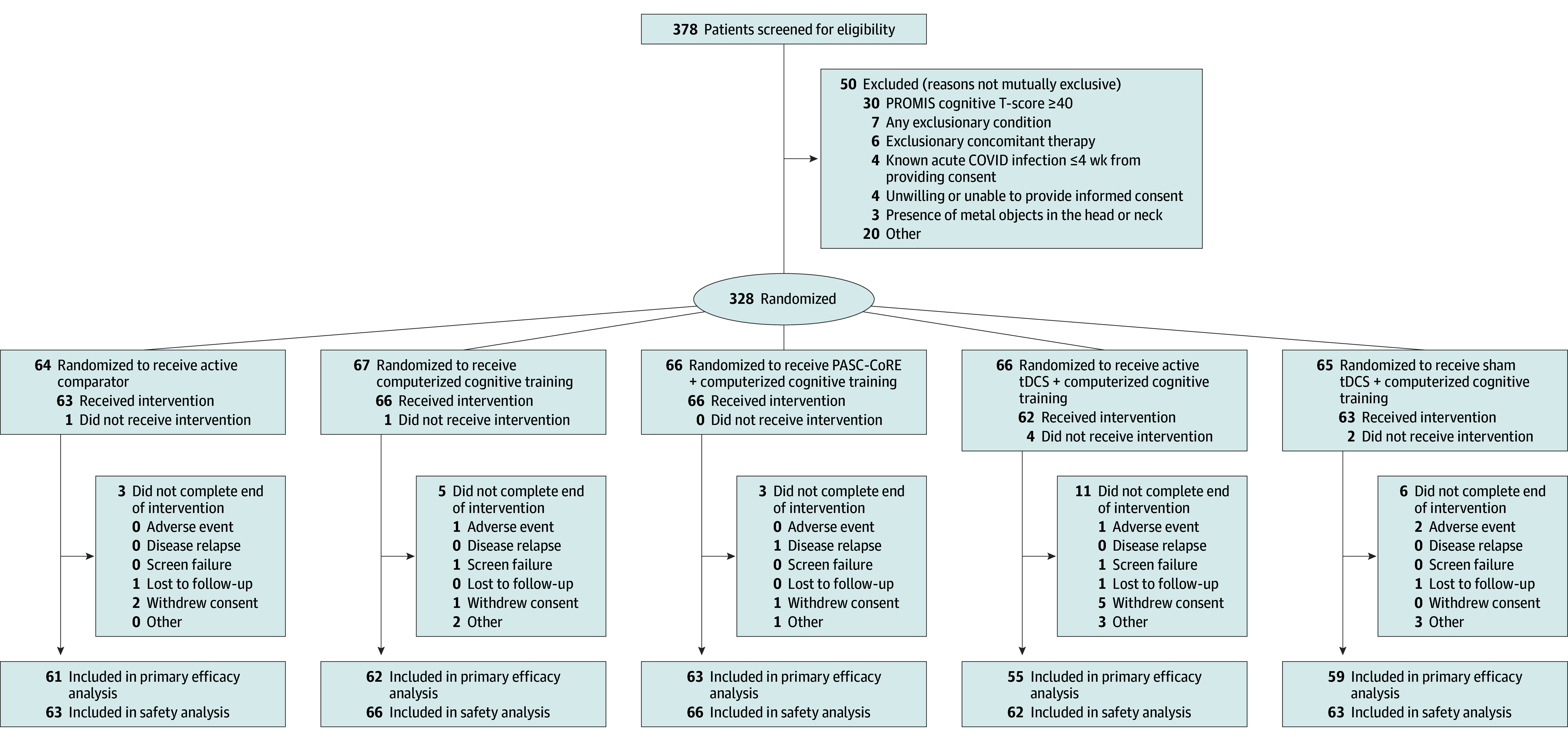
Patient Flow in A Platform Protocol for Evaluation of Interventions for Cognitive Dysfunction in Postacute Sequelae of SARS-CoV-2 Infection (RECOVER-NEURO) Trial PASC indicates postacute sequelae of SARS-CoV-2 infection; PASC-CoRE, PASC-Cognitive Recovery; PROMIS, Patient-Reported Outcomes Measurement Information System; tDCS, transcranial direct current stimulation.

A total of 378 persons were screened for participation from 22 trial sites, 328 were randomized (median [IQR] age, 48.0 [37.0-58.0] years; 241 female [73.5%]; 87 male [26.5%]; race: 15 Asian [4.6%], 47 Black [14.3%], 235 White [71.6%], and 31 other [9.5%]; ethnicity: 52 Hispanic [15.9%]), and 320 (the modified ITT population) received at least 1 intervention (Figure 2 shows the flow of participants). Enrollment occurred between August 17, 2023, and June 10, 2024. Reasons for exclusions and nonrandomizations are presented in eTable 3 in Supplement 2.

Table 1 shows the key demographic characteristics according to treatment assignment. All participants had at least a high school education, and 214 (65%) had a baccalaureate degree or higher. Many participants were on concomitant medications (eTable 4 in Supplement 2). Baseline scores for the modified ECog2, the primary outcome, are shown in Table 2, and baseline scores for the secondary and exploratory outcomes are shown in Table 3. At the baseline visit, participants reported problems with cognition, sleep, depression, anxiety, and fatigue. Neuropsychological test scores in Table 3 were not corrected for age, education, or race and ethnicity. Overall, 176 participants (60.9%) had no cognitive test scores falling −1.5 SDs below expectation based on applying age, sex, and education normative standards; 25 participants (8.7%) had impaired scores on 2 or more cognitive domains. These results will be reported in detail in the future.

**Table 1.** Baseline Characteristics by Treatment Arm

Characteristic	Active comparator (n = 64)	BrainHQ (n = 67)^a^	PASC-CoRE + BrainHQ (n = 66)	tDCS-active + BrainHQ (n = 66)	tDCS-sham + BrainHQ (n = 65)	All participants (N = 328)
Age at enrollment, median (IQR), y	44.5 (37.5-55.0)	49.0 (36.0-59.0)	53.0 (41.0-58.0)	47.0 (37.0-58.0)	49.0 (35.0-57.0)	48.0 (37.0-58.0)
Sex at birth, No. (%)						
Female	52 (81.3)	49 (73.1)	49 (74.2)	46 (69.7)	45 (69.2)	241 (73.5)
Male	12 (18.8)	18 (26.9)	17 (25.8)	20 (30.3)	20 (30.8)	87 (26.5)
Race, No. (%)						
Asian	2 (3.1)	2 (3.0)	6 (9.1)	2 (3.0)	3 (4.6)	15 (4.6)
Black or African American	6 (9.4)	12 (17.9)	7 (10.6)	10 (15.2)	12 (18.5)	47 (14.3)
White	47 (73.4)	46 (68.7)	49 (74.2)	50 (75.8)	43 (66.2)	235 (71.6)
Other^b^	9 (14.1)	7 (10.4)	4 (6.1)	4 (6.1)	7 (10.8)	31 (9.5)
Ethnicity, No. (%)						
Hispanic, Latino, or Spanish	6 (9.4)	12 (17.9)	9 (13.6)	11 (16.7)	14 (21.5)	52 (15.9)
Education, No. (%)						
High school graduate	12 (18.8)	8 (11.9)	14 (21.2)	7 (10.6)	6 (9.2)	47 (14.3)
Some college	9 (14.1)	15 (22.4)	6 (9.1)	21 (31.8)	16 (24.6)	67 (20.4)
Bachelor’s degree	22 (34.4)	23 (34.3)	22 (33.3)	21 (31.8)	27 (41.5)	115 (35.1)
Graduate or professional degree	21 (32.8)	21 (31.3)	24 (36.4)	17 (25.8)	16 (24.6)	99 (30.2)

Abbreviations: PASC, postacute sequelae of SARS-CoV-2 infection; PASC-CoRE, PASC-Cognitive Recovery; tDCS, transcranial direct current stimulation.

^a^ BrainHQ is manufactured by Posit Science.

^b^ Other race included American Indian or Alaska Native, multiracial, Native Hawaiian or Pacific Islander, other, unknown, and not reported.

**Table 2.** Main Outcome Measure (Modified ECog2) in Intention-to-Treat Population by Treatment Arm

Group	Baseline		EOI		Unadjusted change from baseline to EOI		Adjusted difference in mean change vs active comparator (95% CI)^b^	*P* value	Adjusted difference in mean change vs BrainHQ (95% CI)^b^	*P* value	Adjusted difference in mean change vs tDCS sham + BrainHQ (95% CI)^b^	*P* value
	No.	Mean Scores (95% CI)^a^	No.	Mean Scores (95% CI)^a^	No.	Mean (95% CI)						
All (n = 328)	327	2.8 (2.7 to 2.9)	300	2.4 (2.3 to 2.5)	300	−0.4 (−0.5 to −0.3)	NA	NA	NA	NA	NA	NA
Active comparator (n = 64)	64	2.8 (2.6 to 2.9)	61	2.4 (2.2 to 2.6)	61	−0.4 (−0.5 to −0.2)	NA	NA	NA	NA	NA	NA
BrainHQ (n = 67)	67	2.8 (2.7 to 3.0)	62	2.4 (2.2 to 2.6)	62	−0.4 (−0.5 to −0.3)	0.0 (−0.2 to 0.2)	0.98	NA	NA	NA	NA
PASC-CoRE + BrainHQ (n = 66)	66	2.8 (2.7 to 3.0)	63	2.5 (2.4 to 2.7)	63	−0.2 (−0.4 to −0.1)	0.1 (−0.1 to 0.3)	0.18	0.1 (−0.1 to 0.3)	0.17	NA	NA
tDCS-active + BrainHQ (n = 66)	66	2.9 (2.8 to 3.1)	55	2.4 (2.2 to 2.6)	55	−0.5 (−0.7 to −0.4)	−0.1 (−0.3 to 0.1)	0.36	−0.1 (−0.3 to 0.1)	0.37	0.0 (−0.2 to 0.2)	0.97
tDCS-sham + BrainHQ (n = 65)	64	2.7 (2.5 to 2.9)	59	2.2 (2.1 to 2.4)	59	−0.5 (−0.7 to −0.3)	NA	NA	NA	NA	NA	NA

Abbreviation: ECog2, Everyday Cognition Scale 2; EOI, end of intervention; NA, not applicable; PASC, postacute sequelae of SARS-CoV-2 infection; PASC-CoRE, PASC-Cognitive Recovery; tDCS, transcranial direct current stimulation.

^a^ Modified ECog2 has a score from 1 to 5, with 5 being worst.

^b^ Negative adjusted difference in mean change indicates the treatment group in the row did better than the treatment group in the column. BrainHQ is manufactured by Posit Science.

**Table 3.** Secondary Outcome Measures Baseline and Change Scores in Intention-to-Treat Population by Treatment Arm^a^

Outcome	Active comparator (n = 64)		BrainHQ (n = 67)		PASC-CoRE + BrainHQ (n = 66)		tDCS-active + BrainHQ (n = 66)		tDCS-sham + BrainHQ (n = 65)		All participants (N = 328)	
	No.	Mean (95% CI)	No.	Mean (95% CI)	No.	Mean (95% CI)	No.	Mean (95% CI)	No.	Mean (95% CI)	No.	Mean (95% CI)
**Patient-reported outcomes**												
PROMIS Sleep 8a T score^b^												
Baseline	63	57.2 (55.6 to 58.9)	67	57.6 (55.9 to 59.3)	66	58.7 (57.1 to 60.3)	66	59.7 (58.0 to 61.4)	65	58.1 (56.1 to 60.1)	327	58.3 (57.5 to 59.1)
Unadj EOI-BL diff	60	−1.3 (−3.0 to 0.5)	62	−1.0 (−2.9 to 0.9)	63	−0.6 (−1.9 to 0.6)	55	−3.5 (−5.3 to −1.7)	59	−2.2 (−4.0 to −0.5)	299	−1.7 (−2.4 to −0.9)
PROMIS Sleep 8b T score^b^												
Baseline	63	53.3 (52.1 to 54.5)	67	53.3 (52.2 to 54.5)	66	53.8 (52.6 to 55.1)	66	54.0 (53.0 to 55.0)	65	53.4 (52.2 to 54.5)	327	53.6 (53.0 to 54.1)
Unadj EOI-BL diff	60	−0.5 (−1.5 to 0.5)	62	−0.3 (−1.3 to 0.7)	63	−0.2 (−1.1 to 0.7)	55	−1.1 (−2.0 to −0.2)	59	−1.0 (−2.0 to −0.0)	299	−0.6 (−1.0 to −0.2)
PROMIS Cog 8a T score^c^												
Baseline	64	31.3 (30.1 to 32.5)	67	31.0 (29.8 to 32.3)	66	30.8 (29.5 to 32.0)	66	30.4 (29.1 to 31.8)	65	31.1 (29.8 to 32.5)	328	30.9 (30.4 to 31.5)
Unadj EOI-BL diff	61	5.4 (3.5 to 7.3)	62	6.6 (4.3 to 8.9)	63	4.4 (2.6 to 6.3)	55	6.6 (4.7 to 8.6)	59	6.9 (4.9 to 8.8)	300	6.0 (5.1 to 6.8)
PROMIS fatigue T score^b^												
Baseline	64	59.7 (58.4 to 61.0)	67	60.3 (59.1 to 61.6)	66	61.0 (59.8 to 62.2)	66	60.3 (58.7 to 62.0)	65	59.8 (58.3 to 61.2)	328	60.2 (59.6 to 60.8)
Unadj EOI-BL diff	61	−1.2 (−2.3 to −0.2)	62	−1.4 (−2.6 to −0.1)	62	−1.1 (−2.4 to 0.2)	55	−1.9 (−3.5 to −0.4)	59	−1.8 (−3.0 to −0.6)	299	−1.5 (−2.0 to −0.9)
PROMIS depression T score^b^												
Baseline	64	53.3 (51.4 to 55.3)	67	51.9 (49.7 to 54.1)	66	52.5 (50.3 to 54.7)	66	53.8 (51.6 to 56.0)	65	51.4 (49.4 to 53.5)	328	52.6 (51.7 to 53.5)
Unadj EOI-BL diff	61	−2.3 (−3.9 to −0.7)	62	−0.9 (−2.9 to 1.0)	63	−0.9 (−2.5 to 0.7)	55	−2.6 (−4.7 to −0.4)	59	−3.2 (−4.9 to −1.5)	300	−2.0 (−2.8 to −1.2)
PROMIS anxiety T score^b^												
Baseline	64	58.9 (56.9 to 60.8)	67	57.0 (54.9 to 59.2)	66	57.8 (55.6 to 60.0)	66	58.8 (56.2 to 61.4)	65	57.4 (55.2 to 59.6)	328	58.0 (57.0 to 58.9)
Unadj EOI-BL diff	61	−4.3 (−6.2 to −2.4)	62	−0.4 (−2.6 to 1.9)	63	−3.2 (−5.0 to −1.4)	55	−2.8 (−5.3 to −0.3)	59	−4.2 (−6.1 to −2.2)	300	−3.0 (−3.9 to −2.0)
PROMIS distress (raw)^b^												
Baseline	64	17.7 (16.1 to 19.2)	67	16.7 (15.1 to 18.2)	66	17.3 (15.6 to 18.9)	66	18.4 (16.6 to 20.2)	65	16.4 (14.8 to 18.0)	328	17.3 (16.6 to 18.0)
Unadj EOI-BL diff	61	−2.5 (−3.6 to −1.4)	62	−0.4 (−1.8 to 1.0)	63	−1.7 (−2.8 to −0.5)	55	−2.4 (−4.0 to −0.8)	59	−2.7 (−3.9 to −1.6)	300	−1.9 (−2.5 to −1.3)
**Subjective global assessment**												
Do you think the study intervention helped your cognitive functioning?												
EOI: yes, No./No. (%)	41/61 (67.2)		49/62 (79.0)		47/63 (74.6)		43/55 (78.2)		42/58 (72.4)		222/299 (74.2)	
Compared with when you started the study intervention, do you feel that your overall functioning is now												
Much worse, No./No. (%)	0/61 (0)		0/62 (0)		2/63 (3.2)		1/55 (1.8)		1/59 (1.7)		4/300 (1.3)	
Somewhat worse, No./No. (%)	4/61 (6.6)		3/62 (4.8)		5/63 (7.9)		0/55 (0)		4/59 (6.8)		16/300 (5.3)	
Same, No./No. (%)	22/61 (36.1)		13/62 (21.0)		14/63 (22.2)		16/55 (29.1)		16/59 (27.1)		81/300 (27.0)	
Somewhat better, No./No. (%)	30/61 (49.2)		40/62 (64.5)		36/63 (57.1)		34/55 (61.8)		34/59 (57.6)		174/300 (58.0)	
Much better, No./No. (%)	5/61 (8.2)		6/62 (9.7)		6/63 (9.5)		4/55 (7.3)		4/59 (6.8)		25/300 (8.3)	
**Cognitive measures**												
AVLT Trials 1-5^c^												
Baseline	64	46.3 (43.8 to 48.9)	67	47.7 (45.7 to 49.7)	66	47.2 (44.8 to 49.6)	66	47.2 (44.6 to 49.7)	65	47.6 (45.0 to 50.1)	328	47.2 (46.1 to 48.3)
Unadj EOI-BL diff	61	0.4 (−1.6 to 2.5)	62	1.2 (−0.5 to 2.8)	63	1.0 (−0.6 to 2.5)	55	0.5 (−1.3 to 2.4)	59	1.0 (−1.2 to 3.2)	300	0.8 (0.0 to 1.6)
AVLT delayed recall^c^												
Baseline	64	9.6 (8.8 to 10.3)	67	10.1 (9.5 to 10.8)	66	9.8 (9.0 to 10.6)	66	9.7 (9.0 to 10.5)	65	9.7 (8.9 to 10.5)	328	9.8 (9.4 to 10.1)
Unadj EOI-BL diff	61	−0.2 (−1.0 to 0.5)	62	−0.5 (−1.2 to 0.1)	63	0.0 (−0.6 to 0.6)	55	−0.3 (−1.2 to 0.6)	59	−0.1 (−0.9 to 0.7)	300	−0.2 (−0.6 to 0.1)
AVLT correct recognition minus false positives^c^												
Baseline	64	12.5 (11.8 to 13.2)	67	13.0 (12.4 to 13.5)	66	12.8 (12.2 to 13.4)	66	12.8 (12.2 to 13.4)	65	12.3 (11.6 to 13.0)	328	12.7 (12.4 to 12.9)
Unadj EOI-BL diff	58	0.1 (−0.6 to 0.8)	58	0.1 (−0.4 to 0.6)	61	−0.4 (−1.0 to 0.2)	53	0.0 (−0.5 to 0.5)	55	0.5 (−0.5 to 1.4)	285	0.1 (−0.2 to 0.4)
Symbol digit modalities^c^												
Baseline	64	47.5 (45.3 to 49.7)	67	49.2 (46.7 to 51.7)	66	47.4 (45.0 to 49.8)	66	47.9 (45.2 to 50.5)	65	46.9 (44.1 to 49.6)	328	47.8 (46.7 to 48.9)
Unadj EOI-BL diff	61	4.2 (2.3 to 6.2)	62	3.2 (1.3 to 5.1)	63	3.4 (1.6 to 5.2)	55	5.2 (2.8 to 7.6)	59	5.4 (3.1 to 7.8)	300	4.2 (3.3 to 5.2)
Lexical fluency^c^												
Baseline	64	13.5 (12.3 to 14.6)	67	13.8 (12.5 to 15.0)	66	14.2 (13.0 to 15.5)	66	13.3 (12.3 to 14.4)	65	13.2 (12.3 to 14.1)	328	13.6 (13.1 to 14.1)
Unadj EOI-BL diff	61	0.2 (−0.6 to 1.1)	62	1.0 (0.1 to 1.9)	63	0.3 (−0.7 to 1.2)	55	0.6 (−0.3 to 1.6)	59	1.7 (0.8 to 2.5)	300	0.8 (0.4 to 1.2)
Semantic fluency^c^												
Baseline	64	21.0 (19.8 to 22.2)	67	22.0 (20.6 to 23.4)	66	21.4 (19.8 to 22.9)	66	20.3 (19.1 to 21.6)	65	20.3 (18.9 to 21.6)	328	21.0 (20.4 to 21.6)
Unadj EOI-BL diff	61	0.5 (−0.5 to 1.5)	62	0.9 (−0.1 to 2.0)	63	0.9 (−0.3 to 2.1)	55	1.7 (0.5 to 2.9)	59	1.6 (0.5 to 2.7)	300	1.1 (0.6 to 1.6)
Digit vigilance (time)^b^												
Baseline	64	402.3 (380.3 to 424.2)	67	418.1 (396.7 to 439.5)	66	413.5 (390.1 to 437.0)	66	411.2 (384.2 to 438.2)	65	405.6 (383.2 to 428.0)	328	410.2 (400.0 to 420.5)
Unadj EOI-BL diff	61	−16.1 (−32.8 to 0.7)	62	−31.7 (−51.8 to −11.6)	63	−19.2 (−37.5 to −0.9)	54	−37.1 (−56.6 to −17.6)	59	−19.2 (−37.9 to −0.6)	299	−24.4 (−32.6 to −16.2)
Digit vigilance (errors)^b^												
Baseline	64	8.2 (5.8 to 10.6)	67	8.8 (6.0 to 11.5)	66	7.1 (5.4 to 8.8)	66	8.3 (5.6 to 10.9)	65	8.4 (6.0 to 10.7)	328	8.1 (7.1 to 9.2)
Unadj EOI-BL diff	61	−2.0 (−4.1 to 0.2)	62	−1.6 (−4.0 to 0.8)	63	−0.6 (−1.8 to 0.7)	55	−1.5 (−3.5 to 0.6)	59	0.1 (−2.6 to 2.7)	300	−1.1 (−2.0 to −0.2)
NIH Toolbox flanker^c^												
Baseline	61	7.7 (7.4 to 7.9)	66	7.8 (7.6 to 8.1)	66	7.6 (7.4 to 7.9)	61	7.8 (7.6 to 8.1)	60	7.6 (7.3 to 7.9)	314	7.7 (7.6 to 7.8)
Unadj EOI-BL diff	58	0.4 (0.2 to 0.6)	59	0.5 (0.3 to 0.7)	61	0.4 (0.2 to 0.6)	51	0.4 (0.1 to 0.7)	55	0.6 (0.4 to 0.8)	284	0.5 (0.4 to 0.6)

Abbreviations: adj, adjusted; AVLT, Auditory Verbal Learning Test; BL, baseline; Cog8a, Cognitive Function Short Form 8a; diff, difference; EOI, end of intervention; NIH, National Institutes of Health; PASC, postacute sequelae of SARS-CoV-2 infection; PASC-CoRE, PASC-Cognitive Recovery; PROMIS, Patient-Reported Outcomes Measurement Information System; tDCS, transcranial direct current stimulation; unadj, unadjusted.

^a^ No. and mean (95% CI) are presented unless otherwise noted. BrainHQ is manufactured by Posit Science.

^b^ On this test, a decrease in score indicates improvement.

^c^ On this test, an increase in score indicates improvement.

Adherence was excellent in all study arms. Over 90% of participants, with negligible variation between treatment arms, completed more than 80% of intervention activities. Blindedness was generally maintained for active comparator vs BrainHQ and for tDCS active vs sham stimulation (eTable 5 in Supplement 2).

### Primary Outcome

At the baseline visit, the mean (SD) value on the modified ECog2 across all arms was 2.8 (0.70), indicating some difficulty in daily affairs. There were no differences on the modified ECog2 for any of the prespecified primary treatment comparisons in the ITT population (N = 328) (Table 2). The adjusted differences in mean change for the 4 primary contrasts were as follows: BrainHQ vs active comparator, 0.0 (95% CI, −0.2 to 0.2); PASC-CoRE + BrainHQ vs active comparator, 0.1 (95% CI, −0.1 to 0.3); tDCS-active + BrainHQ vs tDCS-sham + BrainHQ, 0.0 (95% CI, −0.2 to 0.2); and PASC-CoRE + BrainHQ vs BrainHQ alone, 0.1 (95% CI, −0.1 to 0.3). The null results were robust to the effects of missing data, as verified by a tipping point analysis. At EOI, the modified ECog2 (relative to baseline) improved in the range of approximately half an SD in the ITT population in all study arms. The modified ECog2 scores at baseline and end of intervention appeared normally distributed and are shown in eFigure 1 in Supplement 2.

Results in the modified ITT population were numerically identical to the ITT analysis. Modified ECog2 outcomes in the per-protocol population (eTable 6 in Supplement 2) and 3 months later at EOS in the ITT population (eTable 7 in Supplement 2) were also similar and failed to show any treatment benefits for any 1 treatment arm but showed less impaired scores for all arms over time. There were no consistent patterns of benefit (or harm) in subgroup analyses for the modified ECog2 (eFigure 2 in Supplement 2).

There was no significant treatment interaction between time from initial COVID infection until randomization. However, we found that improvement across the entire study cohort was inversely related to longer duration of long COVID (modified ECog2 improvement per year = .08; 95% CI, 0.01-0.15; *P* = .02).

### Secondary Outcomes

Neither the participant-reported outcomes nor the neuropsychological tests demonstrated differential benefits for any treatment arm. Groupwise change scores from baseline to EOI for the ITT population are presented in Table 3. Many of the participant-reported secondary outcomes—sleep, fatigue, cognition, depression, and anxiety—showed improved scores by the EOI in all treatment arms, as did some of the change scores from neuropsychological testing such as word list learning, symbol digit test, and fluency tasks. On several subjective global questions administered at the EOI (Table 3), a substantial fraction of participants in all treatment arms reported that the intervention helped their cognitive functioning (222 [74%]) or improved their overall functioning (74 [58%] somewhat improved, 25 [8%] much improved), whereas 20 participants (7%) reported they were somewhat or much worse.

### Safety

A total of 214 participants (76%) reported postexertional malaise at the baseline evaluation. At EOI, 143 participants (56%) reported postexertional malaise, with some variation among treatment arms (eTable 8 in Supplement 2). No participants discontinued prematurely due to postexertional malaise.

In the modified ITT population (n = 320), 11 participants experienced treatment-emergent serious adverse events (eTable 9 in Supplement 2). One participant receiving active tDCS reported a minor thermal injury under 1 of the electrodes 1 month after randomization.

## Discussion

To test the hypothesis that persons with cognitive long COVID would be responsive to rehabilitative strategies, the RECOVER-NEURO trial rapidly recruited 328 adults from 22 sites in the US into a randomized clinical trial of 10 weeks’ duration of 3 remotely administered interventions intended to improve cognitive functioning. Neither BrainHQ, PASC-CoRE + BrainHQ, nor tDCS + BrainHQ exhibited differential benefits on the modified ECog2 primary outcome measure as compared with a program of unstructured mentally challenging puzzles and games or sham tDCS. No support for any of the interventions was found in the secondary outcomes of participant-reported symptoms or neuropsychological tests, nor did a subgroup analysis identify any groups with notable benefits from any intervention. All treatment arms including the active comparator showed improvement of approximately one-half a rating point on the modified ECog2 ordinal 5-point scale, and many secondary outcomes also showed some improvement across treatment arms. All interventions were safe and did not worsen postexertional malaise.

Although our efforts to prove the value of evidence-based therapies were not successful, our experience is mirrored in previous randomized clinical trials. We are unaware of any successful pharmacological interventions for cognitive long COVID, anecdotal claims notwithstanding.^25^ Trials with vortioxetine,^26^ lithium aspartate,^27^ and nirmatrelvir/ritonavir^28^ have failed to show conclusive benefits. One nonpharmacological trial used a cognitive-behavioral approach vs care as usual in 314 persons with long COVID and showed benefits in treated participants.^29^ However, in a trial^30^ involving 98 persons with cognitive long COVID testing, a video game interface targeting attention and executive control administered remotely over 6 weeks, there was no benefit of the intervention compared with a wait-list control, although both treatment groups improved over time. Previous studies using tDCS have also had mixed results,^31,32,33^ and none focused solely on cognitive outcomes.

### Strengths and Limitations

Strengths of the RECOVER-NEURO trial included a rigorous study design with randomized assignment into multiple treatment arms, the use of interventions with prior evidence of benefit for related neurological conditions, an adequate sample size for the clinical benefit desired, an extensive assessment battery, a national sample of participants, and excellent retention (>90%) and protocol adherence (>80%). These outcomes were enabled by a centralized, contactless intervention framework in which participants received individualized study kits or completed all treatment sessions via telehealth video visits. This novel approach to rapid, multisite trials helped reduce participant burden, improve accessibility, and may have contributed to the high completion rates.

However, this study also has some limitations. The uncertainties about the pathobiology of cognitive long COVID made it challenging to select therapies and deliver optimal doses of the interventions. Unexpected therapeutic benefits from the active comparator, nonspecific effects of trial participation, or spontaneous improvement would have made detection of a benefit from 1 of our active interventions very difficult. To maximize generalizability, trial entry was based on subjective cognitive symptoms. Over one-half of our participants did not have performance-based impairment, and thus, results should be interpreted within this context. Future work is warranted to examine any differential impact of intervention on patients with objective cognitive deficits, and future trials may consider stratifying by objective cognitive function.

## Conclusions

In this 10-week, centrally administered trial of 3 remotely delivered interventions for persons with symptoms of cognitive long COVID, no benefits from any active intervention were observed. All treatment arms showed some improvements in self-reported symptoms.

## Notes

Supplement 1.
Trial Protocol.

Supplement 2. **eTable 1.** Inclusion and Exclusion Criteria **eTable 2.** Modified ECog2 (mECog2) Instrument **eTable 3.** Exclusions and Nonrandomizations **eTable 4.** Concomitant Medications **eTable 5.** Maintenance of Blindedness **eTable 6.** Additional mECog2 Outcome for Per-Protocol Analysis at End of Intervention (EOI) **eTable 7.** Additional mECog2 Outcome at End of Study (EOS) **eTable 8.** Postexertional Malaise Across Treatment Groups **eTable 9.** Treatment-Emergent Serious Adverse Events **eFigure 1.** Distribution of mECog2 Scores: ITT Population **eFigure 2.** Subgroup Analysis Forest Plots: ITT Population

Supplement 3. **Nonauthor Collaborators.** RECOVER-NEURO Clinical Trial Group

Supplement 4.
Data Sharing Statement.
