# Supplementary material for: Evaluation of Interventions for Cognitive Symptoms in Long COVID: A Randomized Clinical Trial
Source: JAMA Neurol. 2025 Nov 10;83(1):49–59. doi: 10.1001/jamaneurol.2025.4415 (PMC12603944; doi:10.1001/jamaneurol.2025.4415)
Supplement: Supplement 1. — Trial Protocol. [file jamaneurol-e254415-s001.pdf]

RECOVER-NEURO: A Platform Protocol for Evaluation of Interventions for Cognitive Dysfunction in Post-Acute Sequelae of SARS-CoV-2 Infection (PASC)

National Clinical Trial (NCT) Identified Number
NCT05965752

Co-Principal Investigators

David Knopman, MD
knopman@mayo.edu

Daniel Laskowitz, MD, MHS
daniel.laskowitz@duke.edu

Deborah Koltai, PhD
attix001@duke.edu

Leigh Elkins Charvet, PhD
leigh.charvet@nyulangone.org

Juan Wisnivesky, MD, DrPh
juan.wisnivesky@mountsinai.org

Alex Federman, MD
alex.federman@mountsinai.org

Jacqueline H. Becker, PhD
jacqueline.becker@mountsinai.org

Protocol IND/IDE Sponsor
Kanecia Zimmerman, MD, PhD, MPH

Funded by: National Institutes of Health

Version Number: v3.0

Version Date: 10JAN2024

Table of Contents

List of Tables iv
List of Figures v
STATEMENT OF COMPLIANCE 1
1.1 Synopsis 1
1.2 Schema 2
1.3 Key Roles 3
2 INTRODUCTION 4
2.1 Study Rationale 4
2.2 Background 4
2.3 Risk/Benefit Assessment 5
2.3.1 Known Potential Risks 5
2.3.2 Known Potential Benefits 6
2.3.3 Assessment of Potential Risks and Benefits 6
3 OBJECTIVES AND ENDPOINTS 7
4 STUDY DESIGN 8
4.1 Overall Design 8
4.2 Scientific Rationale for Study Design 8
4.3 Justification for Dose 9
4.4 End of Study Definition 9
5 STUDY POPULATION 9
5.1 Inclusion Criteria 9
5.2 Exclusion Criteria 10
5.3 Lifestyle Considerations 11
5.4 Screen Failures 12
5.5 Study Definition of Enrollment 12
5.6 Strategies for Recruitment and Retention 12
6 STUDY INTERVENTION 13
6.1 Study Intervention(s) Administration 13
6.1.1 Study Intervention Description 13
6.1.2 Dosing and Administration 13
6.2 Measures to Minimize Bias: Randomization and Blinding 13
6.2.1 Unblinding 14
6.3 Study Intervention Adherence 14
6.4 Concomitant Therapy 14
6.4.1 Prohibited Medications 14
6.4.2 Alternative Therapy 16
6.4.3 Rescue Medicine 16
7 PARTICIPANT DISCONTINUATION/WITHDRAWAL 16
7.1 Participant Discontinuation from Intervention 16
7.2 Participant Withdrawal from the Study 17
7.3 Lost to Follow-Up 17
7.4 Study Halting Rules 17
8 STUDY ASSESSMENTS AND PROCEDURES 18

8.1 Schedule of Procedures 18
8.2 Screening (Day -14 to 0) 19
8.3 Baseline (Day -4 to 0) 19
8.4 Start of Intervention (Day 0 + 21 days) 20
8.5 Middle of Intervention (MOI ± 3 days) 20
8.6 End of Intervention (EOI ± 3 days) 20
8.7 End of Study (EOI + 90 days (±3 days)) 21
8.8 Clinical Laboratory Assessments 21
8.9 Study Assessments 21
8.9.1 Everyday Cognition 2 (ECog2) 22
8.9.2 PROMIS-Cog 22
8.9.3 Exploratory PROs 22
8.9.4 Neurocognitive Battery 23
8.10 Biorepository For Future Research 25
9 SAFETY ASSESSMENTS AND REPORTING 25
9.1 Safety Events 25
9.1.1 Definition of Safety Events 25
9.1.2 Collection Period of AE and SAE Information 27
9.1.3 Time Period and Frequency for Event Assessment and Follow-Up 28
9.1.4 Reporting and Monitoring of SAEs and UADEs 29
9.1.5 Events of Special Interest 30
9.1.6 Reporting of Pregnancy 30
10 STATISTICAL CONSIDERATIONS 30
10.1 General Considerations 30
10.2 Statistical Hypotheses 31
10.3 Sample Size Determination 31
10.4 Populations for Analyses 31
10.5 Statistical Analyses 31
10.5.1 Analysis of the Primary Endpoint 31
10.5.2 Analyses of the Secondary Endpoint(s) 32
10.5.3 Analysis of the Exploratory Endpoint(s) 32
10.5.4 Missing Data 32
10.5.5 Planned Interim Analyses 32
11 SUPPORTING DOCUMENTATION AND OPERATIONAL CONSIDERATIONS 33
11.1 Regulatory, Ethical, and Study Oversight Considerations 33
11.1.1 Informed Consent Process 33
11.1.2 Study Discontinuation and Closure 34
11.1.3 Confidentiality and Privacy 34
11.1.4 Key Roles and Study Governance 34
11.1.5 Data and Safety Monitoring Board 35
11.1.6 Clinical Monitoring 35
11.1.7 Quality Assurance and Quality Control 35
11.1.8 Data Handling and Record Keeping 36
11.1.9 Protocol Deviations 36

11.1.10 Publication and Data Sharing Policy 36
11.1.11 Conflict of Interest Policy 37
11.2 Abbreviations 37
11.3 Protocol Amendment History 38
12 REFERENCES 40
13 APPENDIX A 46
13.1 Intervention Rationale 46
13.1.1 BrainHQ 46
13.1.2 PASC-Cognitive Recovery 46
13.1.3 Transcranial Direct Current Stimulation 47
13.2 Exclusion Criteria (Appendix Level) 47
13.3 Study Design 47
13.3.1 Design Rationale 48
13.3.2 Randomization 48
13.3.3 Blinding 49
13.3.4 Schema 49
13.3.5 Control/Comparator 49
13.4 Intervention Procedures 49
13.4.1 BrainHQ 49
13.4.2 PASC-Cognitive Recovery 50
13.4.3 Transcranial Direct Current Stimulation 50
13.4.4 Dosing 51
13.4.5 Schedule of Procedures (Appendix-Level Differences) 51
13.4.6 Adherence 52
13.5 Safety 52
13.6 Events of Special Interest 52
13.7 Pregnancy and Lifestyle Considerations 52
13.8 Statistical Considerations (Appendix Level) 53
13.8.1 Sample Size Determination 53
13.8.2 Primary and Secondary Comparisons 53

List of Tables

Table 1. Summary of study objectives, outcome measures, and endpoints 7
Table 2. Prohibited medications 15
Table 3. Schedule of study procedures 18
Table 4. Comparison studies for Appendix A 48
Table 5. PASC-CoRE session content 50
Table 6. Effect sizes that can be detected with 80% and 90% power under various scenarios for enrollment and loss to follow-up 53

List of Figures

Figure 1. Overall protocol schema 2
Figure 2. Schematic of Appendix A intervention 49
Figure 3. Equipment for tDCS intervention 51

STATEMENT OF COMPLIANCE

The trial will be carried out in accordance with International Conference on Harmonisation Good Clinical Practice (ICH GCP) and the following:

- United States (US) Code of Federal Regulations (CFR) applicable to clinical studies (45 CFR Part 46, 21 CFR Part 50, 21 CFR Part 56, 21 CFR Part 312, and/or 21 CFR Part 812)

National Institutes of Health (NIH)-funded investigators and clinical trial site staff who are responsible for the conduct, management, or oversight of NIH-funded clinical trials have completed Human Subjects Protection and ICH GCP Training.

The protocol, informed consent form(s), recruitment materials, and all participant materials will be submitted to the central Institutional Review Board (IRB) for review and approval. Approval of both the protocol and the consent form(s) must be obtained before any participant is enrolled. Any amendment to the protocol will require review and approval by the IRB before the changes are implemented to the study. In addition, all changes to the consent form will be IRB-approved; a determination will be made regarding whether a new consent needs to be obtained from participants who provided consent, using a previously approved consent form.

1.1 SYNOPSIS

**Title:**	RECOVER-NEURO: A Platform Protocol for Evaluation of Interventions for Cognitive Dysfunction in Post-Acute Sequelae of SARS-CoV-2 Infection (PASC)
**Study Description:**	This platform protocol is designed to be flexible so that it is suitable for a wide range of settings within health care systems, for remote settings, and in community settings where it can be integrated into COVID-19 programs and subsequent treatment plans.<br><br>This protocol is a prospective, multi-center, multi-arm, randomized, controlled platform trial evaluating potential interventions for symptoms of cognitive dysfunction that emerge in patients with PASC. The hypothesis is that PASC-associated dysfunction in cognitive domains, such as executive function and attention, may be improved by interventions that selectively focus on enhancing those domains.
**Objectives:**	Primary:<br>1. Evaluate the intervention's effect on self-reported cognitive function versus comparator<br>Secondary:<br>1. Assess the intervention's effect on cognitive patient-reported outcomes (PROs) versus comparator<br>2. Compare the intervention's effect on an objective neurocognitive battery versus comparator<br>3. Evaluate the intervention's durable effect on self-reported cognitive function versus comparator

	4. Characterize the intervention's safety<br>Exploratory:<br>1. Assess the intervention's effect on exploratory PROs versus comparator
**Study Population:**	Adults self-reporting cognitive dysfunction at least 12 weeks after a SARS-Cov-2 infection.<br><br>The goal is to have a diverse population, including underserved communities and racial/ethnic populations frequently underrepresented in clinical research.
**Phase:**	Phase 2b
**Description of Sites/Facilities Enrolling Participants:**	Participants will be recruited from various acute COVID-19 trials and existing RECOVER initiatives including, but not limited to, the longitudinal cohort as well as other sites and research communities. Up to 45 sites in the US may participate. Though, the number and location of sites may differ among intervention appendices.
**Description of Study Intervention:**	Each intervention (fully described in each appendix) represents a different conceptual approach and control. In general, interventions will target enhancement of cognitive domains that present dysfunction following SARS-CoV-2 infection.
**Participant Duration:**	Participant duration will depend on the specific intervention appendix. After the intervention, participants will have one follow-up visit at 90 days.

1.2 SCHEMA

Figure 1. Overall protocol schema

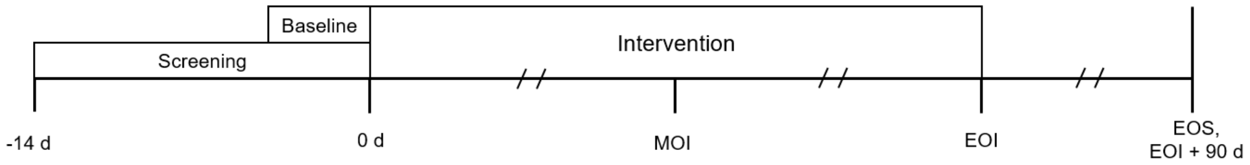

Abbreviations: MOI, Middle of Intervention; EOI, End of Intervention; EOS, End of Study

1.3 KEY ROLES

**Platform Protocol IND Sponsor:**<br>Kanecia Zimmerman, MD, PhD<br>Duke Clinical Research Institute<br>E-mail: kanecia.zimmerman@duke.edu<br>Phone: 919-668-8651	
**Platform Protocol Co-Chairs and Co-Principal Investigators:**<br>David Knopman, MD<br>Mayo Clinic<br>Email: knopman@mayo.edu<br>Phone: 507-266-4106<br><br>Daniel Laskowitz, MD, MHS<br>Duke Clinical Research Institute<br>E-mail: daniel.laskowitz@duke.edu<br>Phone: 919-684-0056	 Deborah Koltai, PhD<br>Duke University Medical Center<br>Email: attix001@duke.edu<br>Phone: 919-668-2846
**Appendix A Co-Principal Investigators:**<br>Leigh Elkins Charvet, PhD<br>NYU Langone Health<br>leigh.charvet@nyulangone.org<br><br>Juan Wisnivesky, MD, DrPh<br>Icahn School of Medicine at Mount Sinai<br>juan.wisnivesky@mountsinai.org<br><br>Jacqueline H. Becker, PhD<br>Icahn School of Medicine at Mount Sinai<br>jacqueline.becker@mountsinai.org<br><br>Alex Federman, MD<br>Icahn School of Medicine at Mount Sinai<br>alex.federman@mountsinai.org	**Appendix A Collaborator:**<br>Henry Mahncke, PhD<br>Posit Science<br>henry.mahncke@positscience.com

2 INTRODUCTION

2.1 STUDY RATIONALE

Post-Acute Sequelae of SARS-CoV-2 infection (PASC), also known as Long COVID, is a chronic condition present in up to 80% of SARS-CoV-2-infected, hospitalized patients and 40% to 70% of non-hospitalized patients with COVID-19.[1-4] The number of PASC patients is escalating, and the personal and global impact of these long-term symptoms from SARS-CoV-2 infection can be debilitating. A prominent PASC symptom is cognitive dysfunction,[5] which can prevent patients' return to work.[6] Therefore, with the increasing number of people infected with SARS-CoV-2, an urgent and unmet clinical need exists to better understand the pathophysiology of PASC and to develop targeted interventions that restore patients' cognitive function. This platform protocol aims to investigate interventions with prior evidence of improving cognitive function. If successful, results will enable providers to treat PASC-related cognitive dysfunction.

2.2 BACKGROUND

In 2019, a novel coronavirus-disease (COVID-19) emerged in Wuhan, China. A month later, the Chinese Center for Disease Control and Prevention identified a new beta-coronavirus (severe acute respiratory syndrome coronavirus 2, or SARS-CoV-2) as the etiological agent.[7] The clinical manifestations of COVID-19 range from asymptomatic infection or mild, transient symptoms to severe viral pneumonia with respiratory failure, and other serious, life-threatening complications.

COVID-19 has led to the death of more than 6 million people worldwide; however, this disease has affected even more lives through often-debilitating symptoms lingering long after acute SARS-CoV-2 infection. Post-Acute Sequelae of SARS-CoV-2 infection affects nearly every organ system, with more than 200 individual symptoms, ranging from new-onset anxiety, depression, and cognitive difficulties to shortness of breath, dizziness, and arrhythmias.[6] Moreover, PASC can occur regardless of severity of acute COVID-19 disease, and it impacts across socioeconomic, racial and ethnic, and age strata. These prolonged symptoms open the door for substantial short- and long-term individual and societal costs, including healthcare costs and inability to work. Prolonged symptoms have kept individuals out of work, which has exacerbated poverty in the underserved, historically minoritized populations and worsened a decades-long mental health crisis. Considering these costs, identification of safe and effective methods to treat and prevent the occurrence of PASC represents an urgent, unmet public health need.

To address this need, the NIH has launched the RECOVER initiative across the nation (RECOVER: Researching COVID to Enhance Recovery) to better understand the disease. The RECOVER Initiative brings together patients, caregivers, clinicians, community leaders, and scientists from across the nation to understand, prevent, and treat PASC. The RECOVER Consortium represents and supports researchers who are leading studies on PASC at more than 200 sites around the country. These studies have a diverse group of participants, including adults, pregnant women, and children. Data from the RECOVER initiative, as well as existing literature, highlight cognitive dysfunction as a frequently reported symptom that is substantially important to patients. Additionally, patients with PASC-related cognitive dysfunction express marked heterogeneity in symptomology and presentation.[8,9]

How COVID-19 induces cognitive dysfunction is not known. Postulated underlying mechanisms include neuroinflammation,[10,11] loss of hippocampal neurons,[10] microglial dysfunction,[11] and neuronal mitochondrial dysfunction.[11] What is known is that, in otherwise healthy adults, symptoms in the neuropsychiatric system cluster include persisting central fatigue,[12-14] central sensitization [15](myalgia,[16] headache[17]), emotional dysregulation[18,19] (depression,[20-22] anxiety[23,24]), and cognitive dysfunction ("brain fog").[5,25] Moreover, this cognitive dysfunction appears to persist for at least 7 to 12 months after acute SARS-CoV-2 infection.[26,27] This long-lasting impact contributes to functional disability, poor quality of life, and psychological morbidity.[28-31] This platform protocol focuses on treating PASC-mediated cognitive dysfunction.

No trials studying interventions directly addressing PASC-related cognitive dysfunction have reported results. One study intervening with olfactory training for olfactory dysfunction in PASC patients did observe memory improvements,[32] supporting the hypothesis that elements of cognitive dysfunction are reversible.

Furthermore, objective and subjective measures evaluating cognition in a PASC population are not well characterized. Objective measures are preferred for analyzing treatment effects on cognition; however, per the RECOVER initiative, only a portion of patients reporting cognitive problems truly have objective dysfunction through assessment. Therefore, to capture patients with objective deficits and those who do not but are still suffering in daily life with cognitive dysfunction, this platform protocol will use a subjective measure, the Everyday Cognition 2 (ECog2), to evaluate the interventions' effects on cognitive dysfunction (see Section 4.2 for detail).

The absence of data, heterogeneity and novelty of PASC, discordance between reported symptoms and objective findings,[8,9,33] and lack of validated measures and endpoints specific to this disease process highlight the importance of studying interventions in the context of well-controlled clinical trials to ensure that optimal and appropriate therapies are made rapidly available for patients. Additionally, clinical trials performed in the PASC population will test the utility of endpoints and outcome measures previously well-established in other disease processes.

2.3 RISK/BENEFIT ASSESSMENT

2.3.1 KNOWN POTENTIAL RISKS

Potential risks of this trial include those associated with the specific intervention (refer to appendices for details), blood draws, nasal swabs, and loss of confidentiality.

Risks associated with blood draws include momentary discomfort and/or bruising. Infection, excess bleeding, clotting, or fainting are also possible, although unlikely.

Risks associated with nasal swabs include mild irritation, insignificant local pain, and minor bleeding.

Loss of confidentiality is a risk. However, coding all participant data with a unique identification number will minimize this risk.

Participation in this study may induce post exertional malaise (PEM) in those prone to PEM or worsen symptoms in those who already have PEM. PEM may be triggered by travel to appointments, extended neurocognitive testing, or active participation in the interventions.

2.3.2 KNOWN POTENTIAL BENEFITS

Participants may benefit directly from improved cognitive function.

Future individuals who acquire PASC-mediated cognitive dysfunction will benefit from knowing effective treatment options.

2.3.3 ASSESSMENT OF POTENTIAL RISKS AND BENEFITS

Potential benefits may outweigh potential risks. Post-acute sequelae of SARS-CoV-2 infection (PASC) is a significant health issue and can have consequences that impact quality of life. These consequences are extremely important for the individual and the society at large.

PEM will be assessed at multiple time points of the interventions.

3 OBJECTIVES AND ENDPOINTS

The objectives, outcome measures, and endpoints for the trial are listed in Table 1. Further details on outcome measures and endpoints are provided in Section 10 and the Statistical Analysis Plan (SAP).

Table 1. Summary of study objectives, outcome measures, and endpoints

OBJECTIVES	OUTCOME MEASURES	ENDPOINTS
Primary		
Evaluate the intervention's effect on self-reported cognitive function versus comparator	Everyday Cognition 2 (ECog2)	Change in average score from baseline to End of Intervention (EOI)
Secondary		
Assess the intervention's effect on cognitive patient-reported outcomes (PROs) versus comparator	PROMIS-cognitive function – short form 8a (PROMIS-Cog)	Change in total score from baseline to EOI and End of Study (EOS), defined as 90 days post-intervention
Compare the intervention's effect on an objective neurocognitive battery versus comparator	• Auditory Verbal Learning Tests<br>• Symbol Digit Modalities Test<br>• Verbal Fluency (lexical + semantic)<br>• Digit Vigilance Test<br>• Cogstate tests: Detection, Identification, One Back<br>• NIH Toolbox Flanker Inhibitory Control and Attention Test	Change from baseline to EOI and EOS
Evaluate the intervention's durable effect on self-reported cognitive function versus comparator	ECog2	Change in average score from baseline to EOS
Characterize the intervention's safety	Serious Adverse Events (SAEs), Unanticipated Adverse Device Effects (UADEs), and/or Events of Special Interest (ESIs)	Proportion of SAEs, UADEs, and/or ESIs
Exploratory		
Assess the intervention's effect on exploratory PROs versus comparator	• PASC Symptom Questionnaire<br>• Patient-reported Outcomes Measurement Information System (PROMIS)-29+2<br>• PROMIS-fatigue – short form 10 a (PROMIS-Fatigue)<br>• PROMIS-8a sleep related impairment (PROMIS-SRI)<br>• PROMIS-8b sleep disturbance (PROMIS-SD)<br>• Modified DePaul Symptom Questionnaire Post Exertional Malaise (DSQ-PEM)	Change in total score from baseline to EOI and EOS

4 STUDY DESIGN

4.1 OVERALL DESIGN

The overall design is a platform protocol designed to be flexible so that it is suitable for a wide range of settings within healthcare systems, for remote settings, and in community settings, where it can be integrated into COVID-19 testing programs and subsequent treatment plans. This platform protocol is a prospective, multi-center, multi-arm, randomized, controlled trial evaluating treatment of PASC-mediated cognitive dysfunction in outpatients previously infected with SARS-CoV-2.

This protocol will recruit adults experiencing PASC symptoms for at least 12 weeks and reporting reduced cognitive function following acute COVID-19 infection. This protocol will enroll participants meeting patient reported outcome criteria for cognitive dysfunction. Participants will consent before being randomly assigned to one of the actively enrolling interventions.

Each appendix will describe an intervention that is sized to meet the platform protocol objectives. Participants will be randomized to one of the intervention appendices that are actively enrolling at the time of randomization. See Section 6.2 for randomization details. Intervention appendices may be added or removed according to adaptive design and/or emerging evidence. Various interventions will be studied; refer to the protocol appendices for further information on each intervention.

This protocol will leverage common data elements (CDEs) already collected as part of the RECOVER initiative as well as assess overall global health status and symptomatology. Organ-specific assessments, including PROs of symptoms and functional status, and objective assessments will also be done. Intervention duration will be specific to an intervention appendix, but all will include a final study assessment 90 days after the intervention. Follow-up will be a combination of in-person visits when necessary to obtain objective clinical assessments and phone calls to be mindful of the participant burden.

4.2 SCIENTIFIC RATIONALE FOR STUDY DESIGN

Each intervention appendix under this platform protocol will follow a randomized trial design to compare the intervention(s) to a comparator. Intervention appendices will target affected cognitive domains, such as executive functioning and attention. Additional interventions can be studied as new interventions are available or as knowledge of PASC-mediated cognitive dysfunction evolves.

Inclusion will be based on patient-reported complaints (PROMIS-Cog) to maximize inclusiveness, because over half of individuals in the RECOVER observational study who had cognitive complaints do not have objective evidence of cognitive dysfunction. The RECOVER-NEURO Working Group recognizes the concern that those participants without objective evidence of cognitive dysfunction may be phenotypically different from those with objective cognitive dysfunction. However, while this is an issue for the FDA, FDA approval is not a goal here.

The primary outcome for this study uses a subjective assessment for cognitive function, the ECog2, instead of or without the addition of an objective assessment for multiple reasons: (a) the inclusion criteria do not require objective criteria; (b) the sensitivity of standard cognitive tests for PASC-related cognitive dysfunction is unknown; and (c) the cognitive deficits of the population under study are still undefined.

Moreover, the original ECog was designed to measure specific aspects of everyday cognition related to memory, language, visuospatial functions, and executive functioning. The ECog2 is clinically relevant and has been validated in the setting of mild cognitive dysfunction, which may increase its sensitivity to the PASC population and to tracking functional outcomes longitudinally. Furthermore, the ECog2 is a robust measure of perceived cognition, spanning across cognitive domains, unlike the other PROs being used. Finally, it has been translated into several languages and validated in a wide range of ethnic/racial minority populations and across educational levels, which increased the likelihood of generalizability.

4.3 JUSTIFICATION FOR DOSE

The dose and duration of the intervention will be based on the specific intervention. Refer to the appendices for details.

4.4 END OF STUDY DEFINITION

The End of Study will occur when all participants have completed their End of Study Visit.

5 STUDY POPULATION

5.1 INCLUSION CRITERIA

In order to be eligible to participate in this study, an individual must meet all of the following criteria:

1. ≥ 18 years of age at the time of enrollment
2. PROMIS-Cog T-score < 40
3. Previous suspected, probable, or confirmed SARS-CoV-2 infection, as defined by the Pan American Health Organization[34]

   Suspected case of SARS-CoV-2 infection - three options, A through C:

   A. Met clinical OR epidemiological criteria:

      a. Clinical criteria: Acute onset of fever AND cough (influenza-like illness) OR Acute onset of ANY THREE OR MORE of the following signs or symptoms: fever, cough, general, weakness/fatigue, headache, myalgia, sore throat, coryza, dyspnea, nausea, diarrhea, anorexia;

      b. Epidemiological criteria: Contact of a probable or confirmed case or linked to a COVID-19 cluster;

B. *Presented acute respiratory infection with history of fever or measured fever of ≥ 38°C and cough, with onset within the last 10 days, and who requires hospitalization; or*

C. *Presented with no clinical signs or symptoms, NOR meeting epidemiologic criteria with a positive professional use or self-test SARS-CoV-2 Antigen-Rapid Diagnostic Test.*

Probable case of SARS-CoV-2 infection, defined as having met clinical criteria above AND was a contact of a probable or confirmed case or was linked to a COVID-19 cluster.

Confirmed case of SARS-CoV-2 infection - two options, A through B:

A. *Presented with a positive nucleic acid amplification test, regardless of clinical criteria OR epidemiological criteria; or*

B. *Met clinical AND/OR epidemiological criteria (See suspected case A), with a positive professional use or self-test SARS-CoV-2 Antigen-Rapid Diagnostic Test.*

** Suspected and probable cases will only be allowed if they occurred before May 1, 2021, and will be limited to 10% of the study population. Otherwise, confirmed cases are required.*

4. Cognitive dysfunction symptoms following a SARS-CoV-2 infection that have persisted for at least 12 weeks and are still present at the time of consent[35,◊]
5. Fluent in English or Spanish language
6. Willing and able to provide informed consent, complete the intervention, complete the intervention assessments, and return for all of the necessary follow-up visits

5.2 EXCLUSION CRITERIA

An individual who meets any of the following criteria will be excluded from participation in this study:

1. Prior or active unstable or progressive major psychiatric or neurologic condition that would not show improvement and could hide treatment effect , at the investigator's discretion, including, but not limited to, the following examples:
   a. Progressive neurodegenerative disease, such as Alzheimer's disease, Parkinson's disease, etc.
   b. Past traumatic brain injury occurrence still associated with active post-concussive symptoms
   c. Uncontrolled seizure disorder, such as having at least one seizure in the last year that is adjudicated by clinical judgment
   d. Post-stroke deficits that may interfere with assessment, such as language or communication difficulties, aphasia, etc.
   e. Formal thought disorders, such as schizophrenia, etc.
   f. Any neuropsychiatric or neurologic disorder uncontrolled for the previous six months or that may interfere with assessment, at discretion of the investigator
2. Known prior diagnosis of myalgic encephalomyelitis/chronic fatigue syndrome, not related to SARS-CoV-2 infection
3. Known active acute SARS-CoV-2 infection ≤ 4 weeks from consent
4. Current use of medications listed in Table 2*
5. History of electroconvulsive therapy
6. Current use of any medication for treating PASC-related symptoms
7. Attention-deficit/hyperactivity disorder (ADHD) diagnosis following the onset of PASC**

8. Current diagnosis of alcohol and substance use disorders
    a. Prior use disorders acceptable if abstinence achieved and maintained for at least 12 months before study enrollment
9. Insufficient visual, auditory, and motor function to participate in intervention and assessments
10. Known pregnancy
11. Current or recent use (within the last 2 months) of the intervention***
12. Known allergy/sensitivity/hypersensitivity to components of the intervention or comparator***
13. Currently receiving/using intervention from another clinical trial, such as another RECOVER trial****
14. Any condition that would make the participant, in the opinion of the investigator, unsuitable for the study
    a. The site investigator has the discretion to determine whether a participant is too cognitively impaired to participate and should instead be referred for clinical evaluation.

** Participants who are currently taking prohibited medications (Table 2) or alternative therapies (Table 2, Section 6.4.2) may undergo a 30-day washout period, which will be determined by the site principal investigator based on standard of care practices. However, if the site principal investigator determines that the participants must continue taking their current medication regimens or alternative therapies, then the participants will be ineligible for enrollment.*

***Participants diagnosed with ADHD prior to PASC-related symptoms and who are compliant on stable doses of medication may participate per the investigator's discretion. Participants should agree to maintain a stable dose of their medication while in the trial.*

**** Relevant if only one intervention appendix is open at the time of enrollment, though exclusion may be qualified in the appendix. If multiple intervention appendices are open, a participant may be excluded from any intervention appendix based on contraindications listed in the intervention appendix, current use of intervention, or known allergy/sensitivity/hypersensitivity and still remain eligible for the remaining intervention appendices.*

*****Following another interventional trial's end-of-study visit, participants must wait 90 days before enrolling into NEURO.*

Exclusions specific to intervention appendices are listed in each appendix.

5.3 LIFESTYLE CONSIDERATIONS

Participants must agree not to begin, resume, or increase the dose of any form of cognitive training or cognitive-enhancing supplements until the end of the active intervention phase of the trial. Participants who stop taking a cognitive-enhancing supplement before being enrolled must complete 30 days of washout before beginning study procedures. Cognitive training is any non-pharmacological intervention that participants started intending to enhance their cognition. A cognitive-enhancing supplement is any non-prescription compound being taken by participants with the goal of enhancing their cognition. Beginning, resuming, or increasing the dose of cognitive training or cognitive-enhancing supplements will result in a protocol deviation.

Participants must agree not to change their current dose of stimulant, atypical stimulant, non-stimulant medication used to treat ADHD, or antidepressant medication while on-study, unless directed by their prescribing clinician, in which case the dose change will be recorded on the eCRF.

Participants capable of becoming pregnant are encouraged to use an effective method of contraception during study intervention administration and for at least 7 days after the final administration of study intervention. If this consideration is not required for a specific appendix, the appendix will expressly state that contraception is not required. Additionally, the appendix will state whether participants can remain in the study if they becomes pregnant.

5.4 SCREEN FAILURES

Screen failures are defined as participants who consent to participate in the clinical trial but are not subsequently randomly assigned to the study intervention or entered in the study. A minimal set of screen failure information is required to ensure transparent reporting of screen failure participants, to meet the Consolidated Standards of Reporting Trials (CONSORT) publishing requirements, and to respond to queries from regulatory authorities. Minimal information includes demography, screen failure details, and eligibility criteria.

5.5 STUDY DEFINITION OF ENROLLMENT

For this study, enrollment is defined as signing consent and completing randomization.

5.6 STRATEGIES FOR RECRUITMENT AND RETENTION

The RECOVER Clinical Trial Data Coordinating Center (CT-DCC) will use an integrated strategy of coordinating with community organizations, the public, and clinical trial sites to identify and retain study participants. To ensure a diverse population is enrolled, strategies from prior successful initiatives will be refined and utilized. The study team will develop a comprehensive communication strategy involving print and social media, as well as leveraging existing organizational structures where possible, to educate the public on concerns about PASC and opportunities for clinical trial participation. Interested members of the public will be provided with information to contact a local site for potential participation.

Participants can be recruited and identified through outreach by participating sites. Site investigators, or their designees, may contact eligible participants to introduce the study and discuss study participation.

Participants may be recruited from other ongoing COVID-19 trials if they opted-in to be contacted about future research opportunities. However, after completing other trials' interventions, including other RECOVER trials, 90 days must lapse before screening for this trial.

Finally, to support participant referral to actively enrolling trials, a series of invitation algorithms based on appendix-specific inclusion/exclusion criteria and participant-entered data may be used. Automatic invitations will be generated for participants who appear eligible based on trial interest, demographics, and medical history. Once participants accept the invitation and adequate consent is obtained, their information will be shared with the applicable study team.

Patient advocates who represent a diverse PASC community will be engaged in the study at every step. Patient advocates will serve as consultants to inform study design, protocol development, and recruitment and retention strategies.

During the active study period, study sites will maintain close connections with study participants.

6 STUDY INTERVENTION

6.1 STUDY INTERVENTION(S) ADMINISTRATION

6.1.1 STUDY INTERVENTION DESCRIPTION

See appendices for full descriptions.

6.1.2 DOSING AND ADMINISTRATION

See appendices for detailed description.

6.2 MEASURES TO MINIMIZE BIAS: RANDOMIZATION AND BLINDING

Since the form of each intervention may differ, the comparators also may differ. In general, the comparator will be a reference intervention against which the effectiveness or safety of an experimental intervention is assessed. To achieve blinding and an equitable randomization probability, a two-step randomization process will be used. The study will employ a simple (unstratified) randomization scheme.

At the first stage, each participant will be assigned with equal probability to one of the intervention appendices for which the participant is eligible, after applying any intervention-specific safety exclusions. At the second stage, each participant will be assigned according to the specific appendix's randomization procedure.

If open intervention appendices have the ability to pool comparators, randomization to either an intervention or comparator will occur at an m:1 ratio, where m equals the number of interventions currently active in the platform protocol and which the participant is eligible to receive. For example, if 3 interventions are active simultaneously with comparators that can be pooled, and if the participant meets the criteria to receive all 3 interventions, the allocation ratio at the first step will be 1:1:1 and the second step 3:1 (intervention vs comparator) for an overall randomization ratio of 1:1:1:1. If 3 interventions are available, but a participant is only eligible to receive 2 of them, the allocation ratio at the first step will be 1:1, and at the second step will be 2:1 (intervention vs comparator) for an overall randomization of 1:1:1. Inclusion of a comparator for each intervention enables masking of study participants and clinical personnel to intervention assignment at the second stage. Participants randomized to the control comparator will only be included in the analyses of the appendices for which they were eligible.

Participants assigned to comparator will be considered part of pooled analyses if the intervention was active at the time of their enrollment and they were eligible to receive that intervention. This will result in approximately a 1:1 allocation ratio for any intervention to pooled comparator. If comparators differ substantially, the comparators will not be pooled.

Sites will be informed to which intervention appendix participants are randomized, but, when applicable, not whether they are allocated to the active intervention arm or comparator arm within that appendix. The participants and investigators will be blinded throughout the study, when possible.

If open intervention appendices do not have the ability to pool comparators but have independent comparators, at the second stage participants will be randomized in a 1:1 ratio to intervention vs comparator inside the specific intervention appendix they were randomized to at the first stage of the randomization procedure.

6.2.1 UNBLINDING

The participants, treating clinicians, and study personnel will remain blinded to intervention versus comparator assignment, when possible, until after the database is locked and final analysis is completed. Only the biostatistical team preparing closed interim reports will be unblinded. Unblinding will occur only if required for participant safety or treatment, at the request of the treating clinician. Refer to the Manual of Procedures (MOP) for further details.

6.3 STUDY INTERVENTION ADHERENCE

Participants will be notified of the importance of adhering to the entire protocol. Adherence definitions and aids may be detailed in each appendix.

6.4 CONCOMITANT THERAPY

For this protocol, a prescription medication is defined as a medication that can be prescribed only by a properly authorized/licensed clinician. Medications to be reported in the Case Report Form (CRF) are relevant concomitant prescription medications, over-the-counter medications, and supplements. Baseline concomitant medications will include all concomitant therapies taken by the participant within 14 days of informed consent.

Categorized as concomitant therapies for this protocol are forms of cognitive training and cognitive-enhancing supplements, both of which are defined in Section 5.3.

6.4.1 PROHIBITED MEDICATIONS

Any medication used for symptomatic treatment of PASC is not permitted during the study.

Table 2 displays medications and therapies and their conditions for being allowed or prohibited for participation. If participants are on prohibited medications and interested in participating, they should be encouraged to discuss discontinuing medications with their clinicians. Additionally, Table 2 lists commonly encountered medication examples. Questions about the status of other medications presenting in these drug classes should be directed to the sponsor.

Table 2. Prohibited medications and therapies

Drug class/Therapy	Generic name (examples)	Brand name (examples)	Allowed	Prohibited	Notes
**Stimulant**	methylphenidate	Adhansia, Aptensio, Concerta, Cotempla, Daytrana, Jornay, Focalin, Metadate, Methylin, Quillichew, Quillivant, Relexxii, Ritalin	Participants who were on stable doses of stimulants used for ADHD *before* their PASC symptoms will not need to washout, can maintain their current dose regimen<br><br>All other instances of stimulant use after a 30-day washout before randomization	All other instances of stimulant use	Dose should be stable ≥ 90 days before randomization and maintained for study duration. A necessary change in dose does not cause discontinuation, but the dose change must be documented in the EDC.
	amphetamine/ dextroamphetamine/ lisdexamphetamine	Adderall, Adzenys, Desoxyn, Dyanavel, Mydayis, Vyvanse			
**Atypical stimulant**	armodafinil	Nuvigil	Participants who were on stable doses of atypical stimulants used for ADHD *before* their PASC symptoms will not need to washout, can maintain their current dose regimen.<br><br>All other instances of atypical stimulant use after a 30-day washout before randomization	All other instances of atypical stimulant use	Dose should be stable ≥ 90 days before randomization and maintained for study duration.
	modafinil	Provigil			
	bupropion	Aplenzin, Budeprion, Buproban, Forfivo, Wellbutrin, Zyban			
	amantadine	Gocovri, Osmolex, Symmetrel			

**Non-stimulant medications used to treat ADHD**	atomoxetine	Strattera	Participants who were on stable doses of non-stimulant medications used for ADHD *before* their PASC symptoms will not need to washout, can maintain their current dose regimen.	Participants on non-stimulant medications used to treat ADHD if the dose is *not* stable or if the ADHD occurred *after* the onset of PASC	Dose should be stable ≥ 90 days before randomization and maintained for study duration.
	viloxazine	Qelbree			
	clonidine	Catapres, Kapvay, Nexiclon			
	guanfacine	Intuniv, Tenex			
**Therapies for cognitive dysfunction used for symptomatic treatment of PASC or premorbid cognitive symptoms**	donepezil	Aricept	After a 30-day washout	Continued treatment	
	memantine	Namenda			
	rivastigmine	Exelon			
	galantamine	Razadyne			
**Narcotics**	hydromorphone	Dilaudid	Occasional use (1-2 times/week) of low-dose narcotics according to local standards.<br><br>All other instances of narcotic use after a 30-day washout before randomization	All other instances of narcotic use are exclusionary, such as higher frequency and dose	use must be refrained within 48 hours prior to assessments; failure to refrain will require rescheduling of the assessments.
	methadone	Dolophine, Methadose			
	meperidine	Demerol, Meperitab			
	oxycodone	Dazidox, Oxaydo, OxyCONTIN, Oxydose, Oxyfast, Roxicodone, Roxybond, Xtampza, Endodan, Percodan, Combunox, Endocet, Magnacet, Narvox, Percocet, Perloxx, Primalev, Roxicet, Roxilox, Tylox, Xartemix, Xolox			
	fentanyl	Lazanda, Duragesic, Ionsys, Sublimaze			
	morphine				
	opium				
	codeine				
	hydrocodone				

Drug class/Therapy	Generic name (examples)	Brand name (examples)	Allowed	Prohibited	Notes
**Benzodiazepines**	diazepam	Valium	Occasional use (1-2 times/week) of low-dose benzodiazepines according to local standards.<br><br>All other instances of benzodiazepine use after a 30-day washout before randomization	All other instances of benzodiazepine use are exclusionary, such as higher frequency and dose	Use must be refrained within 48 hours prior to assessments; failure to refrain will require rescheduling of the assessments.
	alprazolam	Niravam, Xanax			
	triazolam	Halcion			
	lorazepam	Ativan, Loreev			
	clonazepam	KlonoPIN			
**Anticholinergics**	atropine	Atropen	Non-systemically administered (eg, inhaled, topical) anticholinergics, like the inhaled agents for bronchospasm, regardless of frequency and dose	Systematically administered (eg, oral, IV, IM, etc.) anticholinergics are exclusionary, unless it is washed out for 30 days prior to randomization.	
	scopolamine	Maldemar, Scopace, Transderm Scop			
	trihexyphenidyl	Artane, Trihexane, Tritane			
	benztropine	Cogentin			
**Antidepressants**	fluoxetine	PROzac, Rapiflux, Sarafem	Participants who were on stable doses of antidepressants *before* their PASC symptoms will not need to washout, can maintain their current dose regimen.	All other instances of antidepressant use *after* PASC diagnosis are exclusionary, unless it is washed out for 30 days prior to randomization.	Dose should be stable ≥ 90 days before randomization and maintained for study duration. A necessary change in dose does not cause discontinuation, but the dose change must be documented in the EDC.
	duloxetine	Cymbalta			
	citalopram	Celexa			
	sertraline	Zoloft			
	paroxetine	Paxil, Pexeva			
	escitalopram	Lexapro			
	mirtazapine	Remeron			
	bupropion	Wellbutrin			
**Electroconvulsive therapy**			None	Any history of use	
**Transcranial magnetic stimulation therapy**			After a 30-day washout	Continued treatment	

6.4.2 ALTERNATIVE THERAPY

Transcranial magnetic stimulation and electroconvulsive therapy are NOT permitted during the study period. Any lifetime use of electroconvulsive therapy is exclusionary, but previous use of transcranial magnetic stimulation is permitted as long as it has not been performed within 30 days of study initiation.

6.4.3 RESCUE MEDICINE

Participants who require a rescue medication to treat a non-study-related acute condition during the study period should proceed with treatment for the acute condition, as prescribed by their treating clinician. They may continue receiving the intervention regardless of the medication.

7 PARTICIPANT DISCONTINUATION/WITHDRAWAL

7.1 PARTICIPANT DISCONTINUATION FROM INTERVENTION

Discontinuation from an intervention does not mean discontinuation from the study, and remaining study procedures should be completed as indicated by the study protocol. If a clinically significant finding is identified (such as changes from baseline) after enrollment, the investigator or qualified designee will determine if any change in participant management is needed.

An investigator may use his/her discretion to discontinue a participant from the intervention for any reason, including, but not limited to, one of the following:

- Significant study intervention non-compliance
- If any clinical AE, laboratory abnormality, or other medical condition or situation occurs such that continued participation in the study would not be in the best interest of the participant
- Confirmed new case of acute SARS-CoV-2

The reason for participant discontinuation from an intervention will be recorded on the CRF. Participants who are discontinued from the intervention, but who are not withdrawn from the study, will continue to be followed for all study procedures. If participants discontinue an intervention, but do not withdraw consent, they will be followed for safety for at least 28 days.

7.2 PARTICIPANT WITHDRAWAL FROM THE STUDY

Participants are free to withdraw from participation in the study at any time upon request. The study team will attempt to determine a reason for withdrawal; however, participants are not obligated to provide a reason for withdrawal. If obtained, the reason for withdrawal will be recorded on the CRF. No further study procedures will be performed and no further data will be collected from the participant following study withdrawal. All of the data collected up until the time of withdrawal will be maintained in the study database and will be used as the participant's data are evaluable for analysis.

7.3 LOST TO FOLLOW-UP

Participants will be considered lost to follow-up if they fail to return for any scheduled visit *and* if they are unable to be contacted after multiple attempts and methods by the study site staff.

The following actions must be taken if a participant fails to return for a required study visit:

- The site will attempt to contact the participant, reschedule the missed visit, counsel the participant on the importance of maintaining the assigned visit schedule, and ascertain if the participant wishes to and/or should continue in the study.
- Before a participant is deemed lost to follow-up, the investigator or designee will make every effort to regain contact with the participant or next of kin (where possible, telephone calls and, if necessary, a certified letter to the participant's last known mailing address or local equivalent methods). These contact attempts should be documented in the participant's medical record or study file.
- Should the participant continue to be unreachable, after exhausting all methods, he or she will be considered to have withdrawn from the study with a primary reason of lost to follow-up.

7.4 STUDY HALTING RULES

The Data and Safety Monitoring Board (DSMB) will review study data at a regular frequency. During DSMB review, study enrollment and intervention-specific activities will continue. However, if the DSMB recommends to discontinue study activities, study enrollment and intervention-specific activities will be temporarily suspended while the NIH and the study Co-Principal Investigators consider the DSMB recommendations prior to making decisions on study continuation or discontinuation.

8 STUDY ASSESSMENTS AND PROCEDURES

8.1 SCHEDULE OF PROCEDURES

This table displays the general activities schedule for all interventions; however, intervention-specific activities that differ will be listed in the intervention appendix.

Table 3. Schedule of study procedures

	Screening	Baseline[1]	Start of Intervention	Middle of Intervention	End of Intervention	End of Study
**PROCEDURE**	**Day -14 to 0**	**Day -4 to 0**	**Day 0 + 21 days**	**MOI ± 3 days**	**EOI ± 3 days**	**EOI + 90 days (±3 days)**
Informed consent	X					
Demographics	X					
Medical history	X					
COVID treatment		X				
Blood pressure, heart rate, weight		X			X	X
Height	X					
Concomitant medication/therapy	X	X		X	X	X
Appendix-level eligibility criteria	X					
PROMIS-Cog	X			X	X	X
ECog2		X		X	X	X
Neurocognitive battery		X			X	X
Exploratory PROs		X[2]		X[2]	X[2]	X[2]
Randomization		X				
Safety assessment		X	X	X	X	X[3]
DSQ-PEM, twice weekly			X	X	X	
Nasal swab		X				
Pregnancy test		X				
Biorepository collection		X[4]			X[4]	X[5]

[1] Baseline assessments may occur on the same day as Screening

[2] The DSQ-PEM will be performed the immediate next day after completing the neurocognitive battery (Baseline, EOI, EOS) or study visit (MOI).

[3] As needed per intervention

[4] Blood and stool collection

[5] Blood collection only, no stool

8.2 SCREENING (DAY -14 TO 0)

The trial's activities, benefits and risks, and other treatment options will be thoroughly explained to potential participants. After obtaining informed consent, the following assessments will be performed at screening to determine eligibility:

- Demographics
- Medical history
- Height
- Collection of concomitant medications/therapies taken/received within 14 days of informed consent
- PROMIS-Cog*
- Review appendix-level eligibility criteria, refer to appendices

*If Baseline activities do not begin within 7 days of the participant completing the PROMIS-Cog, the PROMIS-Cog must be re-completed, with an inclusive score, before Baseline activities begin.

Participants who fail screening may be re-screened one time. Re-screening more than once is at the site investigator's discretion.

8.3 BASELINE (DAY -4 TO 0)

Baseline assessments may occur on the same day as Screening. The following will occur at the Baseline visit:

- Blood pressure, heart rate, and weight
    - Blood pressure and heart rate measurements should be obtained in the supine and standing positions: Supine measurements will be taken after the participant lies supine for 5 minutes, and then standing measurements after the participant stands for 1 minute.
- COVID treatment, including infection dates, treatment history, and vaccine use
- Concomitant medication/therapy review
- ECog2
- Neurocognitive battery, including the World Health Organization-University of California Los Angeles Auditory Verbal Learning Test (WHO-UCLA AVLT), Symbol Digit Modalities Test, Verbal Fluency (lexical + semantic), Digit Vigilance Test, Cogstate tests (Detection, Identification, One Back), and NIH Toolbox Flanker Inhibitory Control and Attention Test
- Exploratory PROs, including PROMIS-29+2, PROMIS-Fatigue, PROMIS-8a SRI and 8b SD, PASC Symptom Questionnaire, and DSQ-PEM
    - The DSQ-PEM will be performed the immediate next day after completing the neurocognitive battery.
- Biorepository, blood and stool collection (frozen for retrospective analysis)
- Urine or blood pregnancy test

- Nasal Swab, for SARS-CoV-2 rapid antigen test
- Safety assessment, including Serious Adverse Events (SAEs), Suspected Unexpected Serious Adverse Reaction (SUSARs), and Events of Special Interest (ESIs)
- Randomization

Participants should take their medication relative to assessments at consistent intervals across all timepoints. Similarly, the time of day assessments are scheduled should be as consistent as possible across all time points for each participant.

8.4 START OF INTERVENTION (DAY 0 + 21 DAYS)

See appendices for details. Safety assessments, including SAEs, Unanticipated Adverse Device Effects (UADEs), SUSARs, and ESIs, will occur at the start of intervention and continue until EOI.

The DSQ-PEM will be given twice weekly during the intervention, from the Start of Intervention to EOI. The 2 weekly surveys must be separated by at least 48 hours. A DSQ-PEM administered 24 hours after the Baseline, MOI, and EOI visits will not count as one of the two weekly surveys, ie, 3 surveys will be administered in those weeks.

8.5 MIDDLE OF INTERVENTION (MOI ± 3 DAYS)

The Middle of Intervention visit is designed to be fully remote for all interventions. The following will occur:

- Concomitant medication/therapy review
- PROMIS-Cog
- ECog2
- Exploratory PROs, including PROMIS-29+2, PROMIS-Fatigue, PROMIS-8a SRI and 8b SD, PASC Symptom Questionnaire, and DSQ-PEM
    - The DSQ-PEM will be performed the immediate next day after completing the study visit. This does not count as one of the two weekly administrations.
- Safety assessment, including SAEs, UADEs, SUSARs, and ESIs
- DSQ-PEM, twice weekly, separated by at least 48 hours

8.6 END OF INTERVENTION (EOI ± 3 DAYS)

The End of Intervention visit will occur ± 3 days after completing the intervention requirements and duration (see Appendices). The following will occur at the End of Intervention visit:

- Blood pressure, heart rate, and weight
    - Perform blood pressure and heart rate per the procedure explained in the Baseline visit.
- Concomitant medication/therapy review
- PROMIS-Cog
- ECog2

- Neurocognitive battery, including the modified-Rey Auditory Verbal Learning Test (RAVLT), Symbol Digit Modalities Test, Verbal Fluency (lexical + semantic), Digit Vigilance Test, Cogstate tests (Detection, Identification, One Back), and NIH Toolbox Flanker Inhibitory Control and Attention Test
- Exploratory PROs, including PROMIS-29+2, PROMIS-Fatigue, PROMIS-8a SRI and 8b SD, PASC Symptom Questionnaire, and DSQ-PEM
    - The DSQ-PEM will be performed the immediate next day after completing the neurocognitive battery.
- Biorepository, blood and stool collection (frozen for retrospective analysis)
- Subjective Global Assessment Questionnaire
- Safety assessment, including SAEs, UADEs, SUSARs, and ESIs
- DSQ-PEM, twice weekly, separated by at least 48 hours

8.7 END OF STUDY (EOI + 90 DAYS (±3 DAYS))

The following will occur at the End of Study visit:

- Blood pressure, heart rate, and weight
    - Perform blood pressure and heart rate per the procedure explained in the Baseline visit.
- Concomitant medication/therapy review
- PROMIS-Cog
- ECog2
- Neurocognitive battery, including the WHO-UCLA AVLT, Symbol Digit Modalities Test, Verbal Fluency (lexical + semantic), Digit Vigilance Test, Cogstate tests (Detection, Identification, One Back), and NIH Toolbox Flanker Inhibitory Control and Attention Test
- Exploratory PROs, including PROMIS-29+2, PROMIS-Fatigue, PROMIS-8a SRI and 8b SD, PASC Symptom Questionnaire, and DSQ-PEM
    - The DSQ-PEM will be performed the immediate next day after completing the neurocognitive battery.
- Biorepository, blood collection (frozen for retrospective analysis)
- Subjective Global Assessment Questionnaire
- Safety assessment, including SAEs, SUSARs, and ESIs, as needed per appendix

8.8 CLINICAL LABORATORY ASSESSMENTS

Clinical laboratory assessments, such as Complete Blood Count and Comprehensive Metabolic Panel, may be required as part of Baseline assessments. If a participant has these laboratory assessments available within 3 months of study enrollment, they do not need to be repeated as part of the study. See the relevant appendix for requirements.

8.9 STUDY ASSESSMENTS

The following assessments will occur according to the Schedule of Procedures (Table 2). The objective assessments in the secondary outcome will be administered at each study site by at least one person, who will be required to receive training in measurement tool administration (see MOP for details).

8.9.1 EVERYDAY COGNITION 2 (ECOG2)

The ECog2 is a self-report, 41-item questionnaire used to measure the participant's perceived capacity to perform activities related to cognitive function, which could impact major activities of daily living and independence.[36] It has been used for patients with mild cognitive impairment,[37] Alzheimer's Disease,[38] and dementia.[39] It takes 5 minutes to complete.

8.9.2 PROMIS-COG

The PROMIS-Cog is the PROMIS short form for the cognitive function domain and is a self-report, 8-item questionnaire targeting cognitive function in the past seven days.[40] It is a reliable measure with normative data,[41] and takes 2 minutes to complete.

8.9.3 EXPLORATORY PROS

8.9.3.1 PROMIS-29+2

Patient-Reported Outcomes Measurement Information System (PROMIS-29) global health scale: The PROMIS was developed out of the "Roadmap for Medical Research" created by the NIH in 2002 as valid, generalizable items to standardize clinical research across NIH-funded research dealing with PROs. Multiple PROMIS scales have been validated across many clinical populations.[42] The PROMIS-29 consists of 29 items that assess general domains of health and functioning, including overall physical health, mental health, social health, pain, fatigue, and overall perceived quality of life. The PROMIS global health scales has been correlated against the EuroQol EQ-5D.[43] Additionally, PROMIS scales have been used with PASC patients.[44]

The PROMIS-29+2 is used to calculate a preference score (PROPr) by the addition of two Cognitive Function Ability items. Preference-based scores provide an overall summary of HRQOL on a common metric. Preference-based scores summarize multiple domains on a metric ranging from 0 (as bad as dead) to 1 (perfect or ideal health). Scores can be used in comparisons across groups and for cost-utility analyses. It takes 8 minutes to complete.

8.9.3.2 PROMIS-FATIGUE

The PROMIS-Fatigue is the PROMIS short form for the fatigue domain and is a self-report, 10-item questionnaire that assesses a participant's fatigue on a scale of 1 (not at all fatigued) to 5 (very much). It targets fatigue and its impacts on daily living in the past seven days.[40] It is a reliable and valid measure of fatigue across diverse clinical populations.[45] It takes 2 minutes to complete.

8.9.3.3 PROMIS-8A SRI AND 8B SD

The PROMIS 8a SRI and 8b SD were developed as short forms from the PROMIS SD and SRI. The 8-item short forms are strongly correlated with the long forms and have greater precision than other commonly used sleep assessments such as the Pittsburgh Sleep Quality Index and the Epworth Sleepiness Scale. The PROMIS 8b SD form includes a total of 8-items that ask participants to reflect on their sleep over the past 7 days with one question rated very poor to very good and the remaining questions rated not at all to very much. The PROMIS 8a SRI form includes a total of 8-items that ask participants to reflect on their sleep over the past 7 days with questions rated not at all to very much. They take 5 minutes to complete.

8.9.3.4 PASC SYMPTOM QUESTIONNAIRE

The PASC Symptom Questionnaire is a self-report measure for reporting multiple PASC-related symptoms across multiple systems. For this platform protocol, two additional sleep-focused questions will be included. It takes 3 minutes to complete.

8.9.3.5 MODIFIED DEPAUL SYMPTOM QUESTIONNAIRE POST EXERTIONAL MALAISE

The DSQ PEM is a subset of the DSQ that assesses PEM.[46] This scale assesses symptom frequency and severity over the previous 6-month period; however, for the purposes of this trial, the look-back period will be modified to "since the previous visit" when administered at Baseline, MOI, EOI, and EOS. When administered twice weekly, the look-back period will be "the past 7 days." This form was previously validated in patients with myalgic encephalomyelitis/chronic fatigue syndrome. It takes less than 5 minutes to complete.

8.9.3.6 SUBJECTIVE GLOBAL ASSESSMENT QUESTIONNAIRE

The Subjective Global Assessment Questionnaire asks participants to self-report whether the intervention helped their symptoms and whether they think they received an active intervention or active comparator. It takes 2 minutes to complete.

8.9.4 NEUROCOGNITIVE BATTERY

The neurocognitive battery includes measures of objective cognitive function utilizing well-validated, psychometrically robust tests of attentional capacity, executive skill, and memory function. These measures objectively characterize the following:

Learning and Memory:

- Verbal list learning over repeated trials, free recall, and recognition memory

Executive Attentional and Processing Speed Skills:

- Timed sequencing of two sets of stimuli according to a key
- Vigilance tasks: Stimuli identification among simple and complex distractors
- Verbal fluency
- Measures of attention:
    - Simple reaction time; respond when X happens

- Choice reaction time; respond only if X happens

Concentration/working memory; yes/no does new stimuli match previous stimuli

The neurocognitive battery includes the objective measures in the following subsections.

8.9.4.1 AUDITORY VERBAL LEARNING TESTS

The WHO-UCLA AVLT will be used at Baseline and EOS, while a modified-RAVLT will be used at EOI.

The RAVLT is a robust test for clinical assessment of learning and memory and has been validated in English and Spanish.[47,48] The recognition list will be modified to match the number of words in the WHO-UCLA AVLT word list.

Both the RAVLT and WHO-UCLA AVLTare auditory list-learning tests that assess learning and memory and is sensitive to cognitive dysfunction. The tests involve five free recall trials of 15 words, a short delayed recall, a long delayed recall, and a recognition trial.[49] The WHO-AVLT has also been validated in Spanish-speaking populations.[50]

For both AVLTs, the learning trials take 8 minutes to complete, and recall and recognition trials take 5 minutes. Other nonverbal tasks or subjective scales can be administered during the delay period.

8.9.4.2 SYMBOL DIGIT MODALITIES TEST

The Symbol Digit Modalities Test is a neuropsychological executive task measuring sequencing/coding and processing speed.[51] It takes 5 minutes to complete.

8.9.4.3 VERBAL FLUENCY

Verbal Fluency includes neuropsychological measures of lexical and semantic fluency.[52] Each take 3 minutes to complete.

8.9.4.4 DIGIT VIGILANCE

Digit Vigilance is a neuropsychological executive task measuring sustained attention, vigilance, and processing speed.[53] It takes 10 minutes to complete.

8.9.4.5 COGSTATE

Cogstate is a commercially available platform of objective neuropsychological tests for measuring distinct cognitive functions: the Detection Test targeting psychomotor function, the Identification Test targeting attention, and the One Back Test targeting working memory.[54] The Cogstate is validated for telehealth assessment[55] and cognitive impairment.[56] It takes 3-4 minutes to complete each test, about 12 minutes total.

8.9.4.6 NIH TOOLBOX FLANKER INHIBITORY CONTROL AND ATTENTION TEST

The Flanker Test measures both a participant's attention and inhibitory control.[57] The test requires the participant to focus on a stimulus while inhibiting attention to stimuli flanking the stimulus. Scoring is based on a combination of accuracy and reaction time. It takes 3 minutes to complete.

8.10 BIOREPOSITORY FOR FUTURE RESEARCH

The RECOVER Biorepository is designed to collect and store biospecimens, such as blood plasma and serum samples, for future research related to the various studies of the RECOVER Program. Such research might include developing diagnostic and/or prognostic tests, improving our understanding of the underlying pathophysiology of PASC, and developing new therapeutic targets. Samples from biorepositories have proven to be enormously important in the last 20 years, as information on the components of blood has expanded rapidly. Important insights have been gained from biorepository samples from clinical trials and the stored samples from the RECOVER Program will prove equally productive and important. This Biorepository will be conducted under the coordination of the Duke Clinical Research Institute (DCRI) which serves as the CT-DCC for all RECOVER clinical trials.

The existence of the RECOVER Biorepository will provide the opportunity to devise new hypotheses, since blood collection techniques are standardized across all approved protocols, thereby allowing cross-protocol sample comparisons if scientifically justified.

Within this framework, the design of the Biorepository is to collect stool, plasma, and serum for storage in the Biorepository for future, as yet unspecified, analyses, and studies. At each time point including blood collection, eighty (80) mL of blood will be collected to prepare the aliquots for storage at -80 °C. For this protocol, blood will be collected at Baseline, EOI, and EOS. These samples will be stored at the Biorepository in a lab for up to 7 years. See the MOP for details of blood and stool collection.

9 SAFETY ASSESSMENTS AND REPORTING

9.1 SAFETY EVENTS

9.1.1 DEFINITION OF SAFETY EVENTS

An Adverse Event (AE) is any untoward medical occurrence in humans, whether or not considered drug- or intervention-related, which occurs during the conduct of a clinical trial. An AE can therefore be any change in clinical status, routine labs, x-rays, physical examinations, etc., that is considered clinically significant by the study investigator.

A Serious Adverse Event (SAE) or serious suspected adverse reaction (SAR) or serious adverse reaction, as determined by the investigator or the sponsor, is an AE that results in any of the following serious outcomes:

- Death

- Life-threatening AE ("Life-threatening" means that the study participant was, in the opinion of the investigator or sponsor, at immediate risk of death from the reaction as it occurred and required immediate intervention.)
- Persistent or significant incapacity or substantial disruption of the ability to conduct normal life functions
- Inpatient hospitalization or prolongation of existing hospitalization
- Congenital abnormality or birth defect
- Important medical event that may not result in one of the above outcomes, but may jeopardize the health of the study participant or require medical or surgical intervention to prevent one of the above outcomes from occurring

An Adverse Device Effect (ADE) is an event related to the use of an investigational medical device. This includes any AE resulting from insufficiencies or inadequacies in the Instructions for Use, deployment, implantation, installation, or operation, or any malfunction of the investigational medical device. An ADE also includes any event resulting from a use error or intentional misuse.

- Device malfunction – the failure of a device to perform in accordance with the Instructions for Use or clinical investigative plan.
- User error or intentional misuse – a device is used in a manner that is an act or omission of an act that results in a different medical device response than intended by the manufacturer or expected by the user.

An Unanticipated Adverse Device Effect (UADE) is any serious adverse effect, problem, or death caused by or associated with a device if that effect was not previously identified in nature, severity, or degree of incidence in the investigational plan or application (including a supplementary plan or application), or any other unanticipated serious problem associated with a device that relates to the rights, safety, or welfare of subjects. Unanticipated Adverse Device Effects will include events meeting either A or B as stated below:

A. Events meeting ALL of the following criteria:
    - Not included in the relevant appendices, product label, or Instruction for Use
    - Related to the investigational device per the site principal investigator and/or IND sponsor
    - Serious (meets any of the following criteria):
        - Is life-threatening illness or injury
        - Results in permanent* impairment of a body function or a body structure
        - Necessitates medical or surgical intervention to prevent permanent* impairment of a body function or a body structure
        - Results in hospitalization
        - Led to fetal distress, fetal death or a congenital abnormality or birth defect
        - Led to death

    **Permanent* means irreversible impairment or damage to a body structure or function, excluding trivial impairment or damage.

B. Any other unanticipated serious problem associated with the investigational device that relates to the rights, safety, or welfare of subjects.

Hospitalization for elective treatment of a preexisting condition that did not worsen from baseline does not meet the definition of an SAE. Hospitalization is defined as a stay in the hospital exceeding 24 hours.

An unexpected AE is defined as any AE or ADE, the specificity or severity of which is not consistent with the study drugs' package insert or devices' Instruction for Use.

An unanticipated problem involving risk to human subjects or others, including an environmental exposure and exposure to a breastfeeding infant, will be reported to the sponsor and the site IRB and central IRB, as appropriate. The event will not be entered into the electronic data capture (EDC) system. Refer to the MOP and/or Safety Management Plan (SMP) for details.

If an intervention includes study drug(s) and medication errors result in an SAE, the errors are reportable. The medication error will be captured as a protocol deviation and the SAE captured on the SAE electronic Case Report Form (eCRF).

9.1.2 COLLECTION PERIOD OF AE AND SAE INFORMATION

Safety event collection will occur at the pre-specified study visits, but all participants will be instructed to self-report concerns by calling the site.

Serious adverse events (SAEs), UADEs, or ESIs will be extracted by site personnel from the participant's medical record if the participant seeks medical care or if hospitalization occurs, each of which notifies the site to conduct follow-up.

Medical occurrences that begin before intervention procedures, but after obtaining informed consent, will not be considered an AE. The medical occurrence or condition will be captured on the Medical History eCRF.

Non-serious AEs may be reported by the participant, but will not be collected to the study database or further assessed by the site or study personnel. However, any non-serious AEs or ADEs that result in intervention discontinuation will be reported as an AE or ADE in the study database and identified as the reason for discontinuation in the study database, and these non-serious AEs and ADEs will be collected from the start of intervention administration through the end of intervention procedures.

Any AEs that are also classified as symptoms associated with PASC and collected during the study will not be collected as a safety event to the study database or further assessed by the site or study personnel because they will be collected as part of the PASC symptom dataset.

Serious adverse events (SAEs) will be collected from the first study procedure through the End of Study visit [(EOI + 90 days) ± 7 days].

Adverse events (AEs) that qualify as an ESI, even if a non-serious AE, will be collected from the start intervention procedures through the End of Study visit [(EOI + 90 days) ± 7 days]. A UADE observed from

the time of start of intervention through the end of the intervention will be collected in the clinical database.

9.1.2.1 SEVERITY OF EVENT

For reportable events, the following guidelines will be used to describe severity.

- **Mild** – Events require minimal or no treatment and do not interfere with the participant's daily activities.
- **Moderate** – Events result in a low level of inconvenience or concern with the therapeutic measures. Moderate events may cause some interference with functioning.
- **Severe** – Events interrupt a participant's usual daily activity and may require systemic drug therapy or other treatment. Severe events are usually potentially life-threatening or incapacitating. Of note, the term "severe" does not necessarily equate to "serious" for regulatory reporting.

9.1.2.2 RELATIONSHIP TO STUDY INTERVENTION

All reportable events must have their relationship to the study intervention assessed by the clinician, who examines and evaluates the participant based on temporal relationship and his or her clinical judgment. The degree of certainty about causality will be graded using the categories below. In a clinical trial, the study product must always be suspect.

- **Related** – The AE or ADE is known to occur with the study intervention, a reasonable possibility exists that the study intervention caused the AE or ADE, or a temporal relationship subsists between the study intervention and event. Reasonable possibility means that evidence suggests a causal relationship between the study intervention and the AE or ADE.
- **Not Related** – No reasonable possibility exists that the administration of the study intervention caused the event, no temporal relationship subsists between the study intervention and event onset, or an alternate etiology has been established.

9.1.3 TIME PERIOD AND FREQUENCY FOR EVENT ASSESSMENT AND FOLLOW-UP

The occurrence of an AE, ADE, ESI, or SAE may come to the attention of study personnel during study visits and interviews of a study participant presenting for medical care, or upon review by a study monitor.

All reportable events will be captured on the appropriate CRF. Information to be collected includes event description, date/time of onset, clinician's assessment of severity, relationship to intervention product (assessed only by those with the training and authority to make a diagnosis), action taken with intervention product (e.g. discontinuation), and date/time of resolution/stabilization of the event. All events occurring within the pre-specified reportable time windows must be documented appropriately regardless of relationship.

Any medical condition that is present at the time the participant is screened will be considered as baseline and not reported as a safety event. However, if the study participant's condition deteriorates at any time during the study, it will be recorded as a safety event.

Changes in the severity of a safety event will be documented to allow an assessment of the duration of the event at each level of severity to be performed. Safety events characterized as intermittent require documentation of onset and duration of each episode.

The investigator will follow all SAEs or UADEs until resolution, stabilization, or the event is otherwise explained. The DCRI Safety Surveillance Team will follow all SAEs or UADEs until resolution, stabilization, or until otherwise explained.

9.1.4 REPORTING AND MONITORING OF SAES AND UADES

When an intervention appendix comprises a study drug(s), the study drug(s) may be under an IND and subject to IND regulations in 21 CFR 312, especially if their investigational use for treatment of PASC is not an approved indication. In such a case, the IND sponsor, DSMB, or Study Medical Monitor will review aggregate safety data. The IND sponsor or designee will be responsible for determining if the safety reporting criteria are met per 21 CFR 312.32(c)(1)(i)(C) and 21 CFR 312.32(c)(1)(iv) and will notify the CT-DCC to prepare an aggregate report for submission to the US Food and Drug Administration (FDA). An aggregate safety report will be submitted to FDA as soon as possible, but in no case later than 15 calendar days after the IND sponsor determination. If the IND sponsor determines that an unexpected fatal or life-threatening SAR occurs markedly more frequently in a study drug arm than in the comparator arm, an aggregate safety report will be submitted to the FDA as soon as possible, but in no case later than 7 calendar days after the IND sponsor determination. Information on individual SAEs will be available upon request from the FDA following the submission of any aggregate reports.

Any UADE that the IDE sponsor determines is/are reportable will be submitted to the FDA, manufacturer, all reviewing IRBs, and all participating investigators within 10 working days of when the sponsor makes that determination. Any fatal or life-threatening UADE the IDE sponsor determines is reportable will be submitted to the FDA, manufacturer, all reviewing IRBs and all participating investigators within 5 working days of notice of the effect.

If the IDE sponsor determines the UADE presents an unreasonable risk to participants, all investigations or parts of investigations presenting that risk shall be terminated as soon as possible. Termination shall occur not later than 5 working days after the IDE sponsor makes this determination and no later than 15 working days after the sponsor first received notice of the effect.

Individual SAEs or UADEs must be entered into the data system within 24 hours of site awareness. The DCRI Safety Surveillance team will notify pharmaceutical partners of SAEs and device manufacturer of UADEs within 1 to 2 business days of their receipt that occur involving the specific appendix of the supplied study intervention/comparator, as required. Serious Adverse Events that are related and confirmed unlisted by the DCRI Safety Medical Monitor and IND sponsor will be reported to the FDA as SUSARs; as 7-day reports for unexpected fatal or life-threatening adverse reactions and 15-day reports for serious and unexpected adverse reactions. The SUSARs will be shared with the pharmaceutical partner of the supplied study drug according to the same timelines. If the IND sponsor, DSMB, or FDA

note a clinically important increase in the rate of a SUSAR, the IND sponsor or designee will notify investigators no later than 15 calendar days after determining that the information qualifies for reporting. The investigators will notify their local IRB according to local guidelines if applicable. The CT-DCC will notify the central IRB. Refer to the SMP for details regarding specific reporting timelines.

Investigators are not obligated to actively seek information on AEs or SAEs after the participant has concluded study participation. However, if the investigator learns of any SAE, including a death, at any time after a participant has completed the study (but before the study itself has ended), and they consider the event to be reasonably related to the study intervention, the investigator must promptly report the SAE to the sponsor via EDC entry.

9.1.5 EVENTS OF SPECIAL INTEREST

Each intervention may have a unique list of ESIs. Refer to the relevant appendix.

9.1.6 REPORTING OF PREGNANCY

Pregnancies occurring after starting intervention activities and while on-study will be documented in the database. Pregnant participants will be advised to discontinue the intervention. Upon discovery of a pregnancy, the study team will ask pregnant participants to complete a pregnancy-specific consent form in order to follow the pregnancy to its outcome in the case the outcome is not reached while the participant is on-study (i.e., occurs after EOS). Male participants on-study who conceive a child should notify the study team, which will request consent of the mother to follow the pregnancy to its end result. Within 1 to 2 business days after learning of the pregnancy, the DCRI Safety Surveillance team will notify study-drug or device supplying partners of the pregnancy, as required. Lastly, any pregnancy-associated ESI or SAE should be reported if information can be collected and entered into the CRF. Any appendix-specific changes to these reporting requirements are expressly stated in the specific appendix.

10 STATISTICAL CONSIDERATIONS

10.1 GENERAL CONSIDERATIONS

All statistical analyses will be performed using SAS (SAS Institute, Inc. Cary, NC, USA) version 9.4 or higher. Baseline demographic and clinical data will be summarized by intervention arm. Descriptive statistics will include mean, standard deviation, median, 25th and 75th percentiles for continuous variables and frequency and percentage for categorical variables. Statistical comparisons will be performed using two-sided tests at 0.05 significance level. The primary analyses will not be adjusted for multiplicity of comparisons since each intervention appendix can be justified as a separate experiment, as opposed to a part of a family of related experiments (see Proschan and Waclawiw, 2000).[58] Analyses of the secondary endpoints will not be adjusted for multiplicity of comparisons and the results of these analyses will be considered exploratory. Additional details regarding statistical analyses will be provided in the SAP which will be finalized prior to the database lock.

10.2 STATISTICAL HYPOTHESES

Primary Endpoint:

1. Less dysfunction on ECog2 at the EOI compared to baseline in intervention in comparison to comparator condition

Secondary Endpoints:

1. Less dysfunction on the PROMIS-Cog at EOI and at EOS compared to baseline in intervention in comparison to comparator condition
2. Less dysfunction on neurocognitive battery assessments (AVLT, Symbol Digit, Verbal Fluency, Digit Vigilance, Cogstate, Flanker) at EOI and at EOS compared to baseline in intervention in comparison to comparator condition
3. Less dysfunction on ECog2 at EOS compared to baseline in intervention in comparison to comparator condition
4. Study interventions are safe in the PASC population

10.3 SAMPLE SIZE DETERMINATION

This study uses an adaptive platform trial design that will allow interventions to be added or dropped from consideration based on accruing evidence of futility or efficacy, whichever is appropriate to the intervention. In such a design, the required sample size depends on both the number of interventions tested and the ability to pool their comparator arms for analysis. Initial sample size estimates are based on a study with a single intervention and 1:1 allocation to active intervention or comparator. If additional interventions are added later that can contribute to pooled comparator, the sample size will be adjusted accordingly.

See the appendices for intervention-specific details of sample size determination.

10.4 POPULATIONS FOR ANALYSES

Population for effectiveness analyses: ITT. The primary efficacy analysis will be based on an intention-to-treat (ITT) population. All randomized participants will be included and will be analyzed according to their assigned intervention group.

Safety population. Safety analyses will be performed among participants in the ITT population who report completing at least one intervention activity, which is aimed at addressing a study outcome, in the intervention or comparator. Participants will be analyzed according to their assigned intervention groups.

10.5 STATISTICAL ANALYSES

This section describes analysis methods for the primary, secondary, and safety outcomes. Full details will be provided in the SAP.

10.5.1 ANALYSIS OF THE PRIMARY ENDPOINT

The primary endpoint analysis will be performed using a linear regression model with change of ECog2 from baseline to EOI as an outcome. The analysis model will include intervention arm indicator variables and will be adjusted for baseline ECog2, age, sex, education level, and primary language (English vs. Spanish). The normality assumption for the outcome distribution will be evaluated. If this assumption is not met, outcome transformations or regression models with different distributional assumptions may be utilized.

10.5.2 ANALYSES OF THE SECONDARY ENDPOINT(S)

PROMIS-Cog. The PROMIS-Cog is a continuous measure. Changes in total score from baseline to EOI and to EOS will be analyzed using similar methods as those outlined for the primary endpoint.

Neurocognitive dysfunction: Neurocognitive battery (AVLT, Symbol Digit, Verbal Fluency, Digit Vigilance, Cogstate, Flanker). Each of the neurocognitive assessments results in a continuous score. Changes from baseline to EOI and to EOS for each neurocognitive outcome will be analyzed using similar methods as those outlined for the primary endpoint.

ECog2 at EOS. Change in ECog2 from baseline to EOS will be analyzed using similar methods as those outlined for the primary endpoint.

Safety endpoints. Safety endpoints include the proportion of participants who experience individual SAEs/UADEs and the proportion who experience any one or more SAEs/UADEs. These will be analyzed in the safety population. Events of Special Interest (ESIs) will be summarized by intervention appendix. Incidence of AEs/SAEs/UADEs leading to discontinuation will also be summarized.

10.5.3 ANALYSIS OF THE EXPLORATORY ENDPOINT(S)

PROMIS-29+2, PROMIS-Fatigue, PROMIS-SRI, PROMIS-SD, DSQ-PEM, and PASC Symptom Questionnaire. These PROs are continuous measures. Changes from baseline to EOI and to EOS will be analyzed using similar methods as those outlined for the primary endpoint.

10.5.4 MISSING DATA

The primary and secondary analyses will be performed without missing data imputation. Additional sensitivity and supplementary analyses with adjustments for missing data will be described in the SAP.

10.5.5 PLANNED INTERIM ANALYSES

Interim examination of clinical endpoints and key safety events will be performed at regular intervals during the course of the trial. An independent, NIH-appointed, DSMB will monitor participant safety and review performance of the trial. The primary objective of these interim analyses will be to ensure the safety of the participants enrolled in the trial. In addition, interim monitoring will involve a review of participant recruitment, compliance with the study protocol, status of data collection, and other factors which reflect the overall progress and integrity of the study.

This protocol does not have planned early stopping rules for efficacy. Because PASC presentations and outcomes are highly varied, an important study objective is to estimate the effect of treatment on a

wide range of participant-relevant outcomes. If the study were to be stopped early with less than the full sample size, it would decrease precision and reduce the study's ability to characterize intervention risks and benefits based on important secondary effectiveness and safety outcomes. Stopping early would also limit the collection of data that are critical for planning future trials in similar patient populations.

11 SUPPORTING DOCUMENTATION AND OPERATIONAL CONSIDERATIONS

11.1 REGULATORY, ETHICAL, AND STUDY OVERSIGHT CONSIDERATIONS

11.1.1 INFORMED CONSENT PROCESS

11.1.1.1 INSTITUTIONAL REVIEW BOARD (IRB)

The protocol, informed consent form(s) [ICF(s)], recruitment materials, and all participant materials will be submitted to the Institutional Review Board(s) [IRB(s)] of record for review and approval. This approval must be obtained before any participant is enrolled. Any amendment to the protocol will require review and approval by the IRB(s) before being implemented in the study. All changes to the consent form will also be IRB-approved and a determination will be made regarding whether previously consented participants need to be re-consented.

11.1.1.2 CONSENT PROCEDURES AND DOCUMENTATION

All consenting will occur either via an electronic consent process or a paper process. Consent forms describing in detail the intervention/comparator, study procedures, and risks will be given to the participant, and documentation of informed consent is required prior to starting study procedures. Informed consent is a process that is initiated prior to the individual's agreement to participate in the study and continues throughout the individual's study participation. A description of risks and possible benefits of participation will be provided to the participants. Consent forms will be IRB-approved and the participant will be asked to read and review the document. The participant will be provided contact information in the event they have questions about study participation. This will allow them to communicate with the investigators (or their delegate), for further explanation of the research study and to answer any questions that may arise, as necessary. Participants will have the opportunity to carefully review the consent form and ask questions prior to signing.

Participants should have the opportunity to discuss the study and think about it before agreeing to participate. Participants will sign the informed consent document before performing any study procedures. Participants may withdraw consent at any time throughout the course of the study. A copy of the informed consent document will be provided to participants for their records. The rights and welfare of participants will be protected by emphasizing to them that the quality of their medical care will not be adversely affected if they decline to participate in this study.

The study team will distinguish between the desire to discontinue the intervention and the desire to withdraw consent for study follow-up.

11.1.2 STUDY DISCONTINUATION AND CLOSURE

This study may be temporarily suspended or prematurely terminated if there is sufficient reasonable cause. Written notification, documenting the reason for study suspension or termination, will be provided by the sponsor to study participants, site investigators, the central IRB, and the US FDA, as applicable. Study participants will be contacted, as applicable, and be informed of changes to study visit schedule.

Circumstances that may warrant termination or suspension include, but are not limited to, the following:

- Determination of unexpected, significant, or unacceptable risk to participants
- Insufficient compliance to protocol requirements
- Data that are not sufficiently complete and/or evaluable
- Determination that the primary endpoint has been met
- Determination of futility

The study may resume once concerns about safety, protocol compliance, and data quality are addressed, and satisfy the sponsor, IRB, and/or FDA.

11.1.3 CONFIDENTIALITY AND PRIVACY

Participant confidentiality is strictly held in trust by the participating investigators, their staff, and the sponsor(s) and their agents. This confidentiality is extended to cover testing of biological samples in addition to the clinical and private information relating to participants. Therefore, the study protocol, documentation, data, and all other information generated will be held in strict confidence. No information concerning the study or the data will be released to any unauthorized third party without prior written approval of the sponsor. The study participant's contact information will be securely stored in the clinical study database.

Study participant research data, which is for purposes of statistical analysis and scientific reporting, will be transmitted to and stored at the CT-DCC. The study data entry and study management systems used by clinical sites and by research staff will be secured and password protected. At the end of the study, all study-related data storage systems will be archived according to local processes.

11.1.4 KEY ROLES AND STUDY GOVERNANCE

The RECOVER program is overseen by the RECOVER Executive Committee. The RECOVER program also includes a Clinical Trial Steering Committee, which is a multi-stakeholder committee that oversees the study and includes patients, the CT-DCC, the NIH, the FDA, and academic and subject matter experts.

The CT-DCC is overseen by a Principal Investigator. The CT-DCC is responsible for study coordination, site management, communication, financial administration, treatment allocations, receipt and processing of data, quality control programs, and statistical analysis and reporting.

The DSMB will oversee the safety and welfare of trial participants as well as provide recommendations for continuation, discontinuation, or revision of the trial.

11.1.5 DATA AND SAFETY MONITORING BOARD

Safety oversight will be under the direction of the RECOVER DSMB composed of individuals with the appropriate expertise. Members of the DSMB should be independent from the study conduct and free of conflict of interest, or measures should be in place to minimize perceived conflict of interest. The DSMB will meet at least semi-annually to approve protocols, assess safety and efficacy data, and at appropriate intervals to meet requirements for the Interim Analyses on each arm of the study. The DMSB will operate under the rules of an approved charter that will be written and reviewed at the organizational meeting of the DSMB. At this time, each data element that the DSMB needs to assess will be clearly defined. The DSMB will provide its input to the NIH.

11.1.6 CLINICAL MONITORING

This study will employ a centralized, remote, risk-based approach to monitoring with routine and periodic review of site-submitted data to review the informed consent process, select eligibility criteria, medical history, identify and follow-up on missing data, inconsistent data, data outliers, etc. and ensure completion of administrative and regulatory processes. The study team will facilitate regular communication through training sessions, teleconferences, videoconferencing, email, etc. Using quality-by-design principles, steps will be taken at the study design stage to foresee and limit significant problems that might occur during the study conduct.

11.1.7 QUALITY ASSURANCE AND QUALITY CONTROL

The study team will work in tandem to ensure that the data collected in this study are as complete and correct as possible. A four-step, multi-functional approach to quality control will be implemented:

1. **Training.** Prior to the start of enrollment, the clinician investigators and key study personnel at each site will be trained with the clinical protocol and data collection procedures, including how to use the EDC system. Follow-up training and training for new study personnel or new versions of the protocol will be conducted as needed.

2. **Monitoring.** The RECOVER CT-DCC will ensure that data collection is handled properly, will provide in-service training, and will address questions from site investigators and coordinators. Electronic review of data quality and completeness will occur on a regular and ongoing basis. Any issues will be addressed.

3. **Managing data.** After the data have been transferred for statistical summarization, data description, and data analysis, further crosschecking of the data will be performed with discrepant observations being flagged and appropriately resolved through a data query system.

4. **Reviewing data.** Data regarding events of interest will be reviewed to ensure appropriate documents are collected for DSMB review. The CT-DCC will monitor study data and contact site study teams when events comprising the primary endpoint are not complete.

11.1.8 DATA HANDLING AND RECORD KEEPING

11.1.8.1 DATA COLLECTION AND MANAGEMENT RESPONSIBILITIES

Streamlining research activities and conducting the trial in a pragmatic manner will increase the ability to complete the trial in the face of strained clinical and research resources. Data may be collected by electronic methods, supplemented by telephone or videophone follow-up, and from the electronic health record.

Data will be collected directly from participants using REDCap through text messaging or email with a survey link, or a site-based computer, or phone call as back up. The process for using text messaging and email is Health Insurance Portability and Accountability Act (HIPAA) compliant.

Site personnel or participants will enter study data into a secure online database. Data will be maintained in a secure online database until the time of study publication. At the time of publication, the CT-DCC will generate a de-identified version of the database for archiving (see Section 11.1.10). All source documents at the sites should be completed in a neat, legible manner to ensure accurate interpretation of data.

11.1.8.2 STUDY RECORDS RETENTION

Study documents should be retained for a minimum of six years after the study has ended. However, if required by local regulations or the US FDA, these documents should be retained for a longer period. No records will be destroyed without the written consent of the sponsor. The sponsor is responsible for informing the investigator when these documents no longer need to be retained.

11.1.9 PROTOCOL DEVIATIONS

A protocol deviation is defined as non-compliance with the clinical study protocol or GCP requirements. The non-compliance may be on the part of the participant, site investigator, or the site staff.

A major protocol deviation is a significant divergence from the protocol that may have significant effect(s) on the participant's safety, rights, or welfare and/or on the integrity of the study data. Major protocol deviations must be sent to the study IRB and local IRB per their guidelines, recorded in source documents, and reported to the coordinating center. Major protocol deviations will be tracked. For this study, any missed or delayed survey completion will not be considered a major protocol deviation, unless the survey is required for the primary endpoint.

11.1.10 PUBLICATION AND DATA SHARING POLICY

This study will comply with the NIH Public Access Policy, which ensures that the public has access to the published results of NIH funded research. The Policy requires scientists to submit final peer-reviewed

journal manuscripts, which arise from NIH funds, to the digital archive PubMed Central upon acceptance for publication.

11.1.11 CONFLICT OF INTEREST POLICY

The independence of this study from any actual or perceived influence, such as by the pharmaceutical industry, is critical. Therefore, any actual conflict of interest of persons who have a role in the design, conduct, analysis, publication, or any aspect of this trial must be disclosed and managed. Furthermore, persons who have a perceived conflict of interest will be required to have such conflicts managed in a way that is appropriate to their participation in the design and conduct of this trial. The study leadership in conjunction with the NIH has established policies and procedures for all study group members to disclose all conflicts of interest and will establish a mechanism for the management of all reported dualities of interest.

11.2 ABBREVIATIONS

AE	Adverse Event
ADE	Adverse Device Effect
ADHD	Attention-deficit/hyperactivity disorder
CDE	Common Data Element
CFR	Code of Federal Regulations
CONSORT	Consolidated Standards of Reporting Trials
CoRE	Cognitive Recovery
COVID-19	Coronavirus Disease of 2019
CRF	Case Report Form
CT-DCC	Clinical Trial – Data Coordinating Center
DCRI	Duke Clinical Research Institute
DSMB	Data Safety Monitoring Board
ECog2	Everyday Cognition 2
eCRF	Electronic Case Report Forms
EDC	Electronic Data Capture
EOI	End of Intervention
EOS	End of Study
ESI	Events of Special Interest
FDA	Food and Drug Administration
GCP	Good Clinical Practice
HIPAA	Health Insurance Portability and Accountability Act
ICF	Informed Consent Form
ICH	International Conference on Harmonisation
IDE	Investigational Device Exemption
IND	Investigational New Drug
IRB	Institutional Review Board

ITT	Intention-To-Treat
MOP	Manual of Procedures
NCT	National Clinical Trial
NIH	National Institutes of Health
PASC	Post-acute Sequelae of SARS-CoV-2 Infection
PI	Principal Investigator
PRO	Patient Reported Outcome
PROMIS	Patient-reported Outcomes Measurement Information System
PROMIS-Cog	PROMIS-Cognitive Function – short form 8a
PROMIS-Fatigue	PROMIS-Fatigue – short form 10a
PROMIS-SD	PROMIS-8a Sleep Disturbance
PROMIS-SRI	PROMIS-8a Sleep Related Impairment
RAVLT	Rey Auditory Verbal Learning Test
RECOVER	Researching COVID to Enhance Recovery
SAE	Serious Adverse Event
SAP	Statistical Analysis Plan
SAR	Suspected Adverse Reaction
SARS-CoV-2	Severe Acute Respiratory Syndrome Coronavirus 2
SMP	Safety Management Plan
SUSAR	Suspected Unexpected Serious Adverse Reaction
tDCS	Transcranial Direct Current Stimulation
UADE	Unanticipated Adverse Device Effect
US	United States
WHO-UCLA AVLT	World Health Organization-University of California Los Angeles Auditory Verbal Learning Test

11.3 PROTOCOL AMENDMENT HISTORY

Version	Date	Description of Change
1.0	01MAY2023	None, original protocol
2.0	09JUN2023	• Added risk posed to participants with post-exertional malaise (Section 2.3.1)<br>• Replaced the Rey Auditory Verbal Learning Test with the WHO-UCLA Verbal Learning Test (Section 8.9.4.1)<br>• Added sub-exclusion to exclusion 12 to convey an investigator has discretion to determine whether a participant is too cognitively impaired to participate and should be referred for clinical evaluation (Section 5.2)<br>• Added rationale for the neurocognitive battery (Section 8.9.4)<br>• Changed use of PROMIS 8a SD to 8b SD

		• Added time burden to PASC Symptom Questionnaire (Section 8.9.3.4)<br>• Added pregnancy requirement for female partners of male participants to platform protocol (Section 9.1.6)<br>• Added pregnancy and contraception considerations to Appendix A that are different than those in the platform (Appendix A)<br>• Added stool to blood collection (Section 8.10)<br>• Added Subjective Global Assessment Questionnaire at End of Intervention and End of Study (Section 8.9.3)<br>• Defined cognitive training, cognitive-enhancing supplement (Section 5.3)<br>• Added anticholinergics as allowable except within 48 hours of assessments (Section 5.3)<br>• Added that "Failure to refrain from [benzodiazepine, anticholinergic, narcotic] their use in this time frame will require rescheduling of the assessments." (Section 5.3)<br>• Added concomitant medication/therapy review at MOI (Section 8.1)<br>• Changed “control” to “comparator” throughout platform<br>• Added “±3 Days” to EOS visit<br>• Added unanticipated adverse device effects to a secondary outcome<br>• Clarified that Clinical laboratory assessments will not be required as part of Baseline assessments. (Section 13.4.5)<br>• Added twice weekly DSQ-PEM assessments throughout the intervention (Section 8.1)<br>• Other administrative changes, such as grammar and punctuation
3.0	10JAN2024	• Added NCT numbers to the cover page for the platform protocol and to Appendix A<br>• Edited the primary endpoint from change in total score to change in average score (Section 3)<br>• Added the exclusion of ADHD diagnosis treated with stimulants that cannot be discontinued (Section 5.2)<br>• Added the exclusion of lifetime use of electroconvulsive therapy (Section 5.2)<br>• Edited exclusion #4 to direct the reader to prohibited medications in Table 2 (Section 5.2)<br>• Added a footnote to exclusion #4 that allows for a washout period of prohibited medications (Section 5.2)<br>• Edited exclusion #6 from any stimulant treating PASC to any medication treating PASC (Section 5.2)<br>• Added Table 2 for prohibited medications and therapies<br>• Edited section 6.4.1 to be for prohibited medications

		• Added a 30-day washout requirement for participants who stop taking a cognitive-enhancing supplement before being enrolled (Section 5.3)<br>• Moved and edited language from Section 5.3 about occasional use of drugs to Section 6.4.1 with Table 2<br>• Added 21-day buffer to Start of Intervention due the potential time required for forming PASC-CoRE groups (Section 8.1)<br>• Added blood pressure and heart rate collection to the Baseline, EOI, and EOS visits (Section 8.1)Added that participants cannot change their stimulant dose while on-study (Section 5.3)<br>• Added that 90 days must lapse after completing another trial before screening for this one (Section 5.6)<br>• Added statements to clarify randomization (Section 6.2)<br>• Added alternative therapy instructions (Section 6.4.2)<br>• Removed that participants may be replaced (Section 7.1)<br>• Added that “Participants who fail screening may be re-screened one time.” (Section 8.2)<br>• Separated COVID treatment from medical history and moved COVID treatment to Baseline (Section 8.3)<br>• Made the term “cognitive dysfunction” consistent throughout<br>• Edited the look-back periods for the DSQ-PEM (Section 8.9.3.5)<br>• Rey Auditory Verbal Learning Test added as an AVLT with the WHO-UCLA AVLT, with corresponding changes made throughout (Section 8.9.4.1)<br>• Added that pregnancy testing is not required for female participants who self-report menopause, have no menstrual cycle for at least 12 months, or are surgically sterile (Section 13.7)<br>• Added pregnancy considerations to Appendix A (Section 13.7)<br>• Updated the primary comparisons in Appendix A (Section 13.8.2)<br>• Made punctuation changes throughout

12 REFERENCES

1. Cabrera Martimbianco AL, Pacheco RL, Bagattini Â M, Riera R. Frequency, signs and symptoms, and criteria adopted for long COVID-19: A systematic review. *Int J Clin Pract*. Oct 2021;75(10):e14357. doi:10.1111/ijcp.14357

2. Huang C, Huang L, Wang Y, et al. 6-month consequences of COVID-19 in patients discharged from hospital: a cohort study. *Lancet*. Jan 16 2021;397(10270):220-232. doi:10.1016/s0140-6736(20)32656-8

3. Hirschtick JL, Titus AR, Slocum E, et al. Population-Based Estimates of Post-acute Sequelae of Severe Acute Respiratory Syndrome Coronavirus 2 (SARS-CoV-2) Infection (PASC) Prevalence and Characteristics. *Clin Infect Dis*. Dec 6 2021;73(11):2055-2064. doi:10.1093/cid/ciab408

4. Bell ML, Catalfamo CJ, Farland LV, et al. Post-acute sequelae of COVID-19 in a non-hospitalized cohort: Results from the Arizona CoVHORT. *PLoS One*. 2021;16(8):e0254347. doi:10.1371/journal.pone.0254347

5. Woo MS, Malsy J, Pottgen J, et al. Frequent neurocognitive deficits after recovery from mild COVID-19. *Brain Commun*. 2020;2(2):fcaa205. doi:10.1093/braincomms/fcaa205

6. Davis HE, Assaf GS, McCorkell L, et al. Characterizing long COVID in an international cohort: 7 months of symptoms and their impact. *EClinicalMedicine*. Aug 2021;38:101019. doi:10.1016/j.eclinm.2021.101019

7. Zhu N ZD, Wang W. A Novel Coronavirus from Patients with Pneumonia in China. *N Engl J Med* 2020;382:727-733.

8. Goodman BP, Khoury JA, Blair JE, Grill MF. COVID-19 Dysautonomia. *Front Neurol*. 2021;12:624968. doi:10.3389/fneur.2021.624968

9. Barizien N, Le Guen M, Russel S, Touche P, Huang F, Vallée A. Clinical characterization of dysautonomia in long COVID-19 patients. *Sci Rep*. Jul 7 2021;11(1):14042. doi:10.1038/s41598-021-93546-5

10. Klein R, Soung A, Sissoko C, et al. COVID-19 induces neuroinflammation and loss of hippocampal neurogenesis. *Res Sq*. Oct 29 2021;doi:10.21203/rs.3.rs-1031824/v1

11. Stefano GB, Buttiker P, Weissenberger S, Martin A, Ptacek R, Kream RM. Editorial: The Pathogenesis of Long-Term Neuropsychiatric COVID-19 and the Role of Microglia, Mitochondria, and Persistent Neuroinflammation: A Hypothesis. *Med Sci Monit*. May 10 2021;27:e933015. doi:10.12659/MSM.933015

12. Nalbandian A, Sehgal K, Gupta A, et al. Post-acute COVID-19 syndrome. *Nature Medicine*. 2021/04/01 2021;27(4):601-615. doi:10.1038/s41591-021-01283-z

13. Carfi A, Bernabei R, Landi F, Gemelli Against C-P-ACSG. Persistent Symptoms in Patients After Acute COVID-19. *JAMA*. Aug 11 2020;324(6):603-605. doi:10.1001/jama.2020.12603

14. Mantovani E, Mariotto S, Gabbiani D, et al. Chronic fatigue syndrome: an emerging sequela in COVID-19 survivors? *J Neurovirol*. Aug 2021;27(4):631-637. doi:10.1007/s13365-021-01002-x

15. Bierle DM, Aakre CA, Grach SL, Salonen BR, Croghan IT, Hurt RT, Ganesh R. Central Sensitization Phenotypes in Post Acute Sequelae of SARS-CoV-2 Infection (PASC): Defining the Post COVID Syndrome. *J Prim Care Community Health*. Jan-Dec 2021;12:21501327211030826. doi:10.1177/21501327211030826

16. Valle A, Roizenblatt S, Botte S, et al. Efficacy of anodal transcranial direct current stimulation (tDCS) for the treatment of fibromyalgia: results of a randomized, sham-controlled longitudinal clinical trial. *J Pain Manag*. 2009;2(3):353-361.

17. Przeklasa-Muszynska A, Kocot-Kepska M, Dobrogowski J, Wiatr M, Mika J. Transcranial direct current stimulation (tDCS) and its influence on analgesics effectiveness in patients suffering from migraine headache. *Pharmacol Rep*. Aug 2017;69(4):714-721. doi:10.1016/j.pharep.2017.02.019

18. Renaud-Charest O, Lui LMW, Eskander S, et al. Onset and frequency of depression in post-COVID-19 syndrome: A systematic review. *Journal of Psychiatric Research*. 2021/12/01/ 2021;144:129-137. doi:*https://doi.org/10.1016/j.jpsychires.2021.09.054*

19. Lorkiewicz P, Waszkiewicz N. Biomarkers of Post-COVID Depression. *J Clin Med*. Sep 14 2021;10(18)doi:10.3390/jcm10184142

20. Alonzo A, Fong J, Ball N, Martin D, Chand N, Loo C. Pilot trial of home-administered transcranial direct current stimulation for the treatment of depression. *J Affect Disord*. Jun 1 2019;252:475-483. doi:10.1016/j.jad.2019.04.041

21. Brunoni AR, Boggio PS, De Raedt R, et al. Cognitive control therapy and transcranial direct current stimulation for depression: a randomized, double-blinded, controlled trial. *J Affect Disord*. Jun 2014;162:43-9. doi:10.1016/j.jad.2014.03.026

22. D'Urso G, Dell'Osso B, Rossi R, et al. Clinical predictors of acute response to transcranial direct current stimulation (tDCS) in major depression. *J Affect Disord*. Sep 2017;219:25-30. doi:10.1016/j.jad.2017.05.019

23. D'Urso G, Mantovani A, Patti S, Toscano E, de Bartolomeis A. Transcranial Direct Current Stimulation in Obsessive-Compulsive Disorder, Posttraumatic Stress Disorder, and Anxiety Disorders. *J ECT*. Sep 2018;34(3):172-181. doi:10.1097/YCT.0000000000000538

24. de Lima AL, Braga FMA, da Costa RMM, Gomes EP, Brunoni AR, Pegado R. Transcranial direct current stimulation for the treatment of generalized anxiety disorder: A randomized clinical trial. *J Affect Disord*. Dec 1 2019;259:31-37. doi:10.1016/j.jad.2019.08.020

25. Even Mild Cases Can Cause “COVID-19 Fog”. Accessed December 8, 2022. *https://www.cuimc.columbia.edu/news/even-mild-cases-can-cause-covid-19-fog*

26. Liu YH, Chen Y, Wang QH, et al. One-Year Trajectory of Cognitive Changes in Older Survivors of COVID-19 in Wuhan, China: A Longitudinal Cohort Study. *JAMA Neurol*. May 1 2022;79(5):509-517. doi:10.1001/jamaneurol.2022.0461

27. Becker JH, Lin JJ, Doernberg M, Stone K, Navis A, Festa JR, Wisnivesky JP. Assessment of Cognitive Function in Patients After COVID-19 Infection. *JAMA Netw Open*. Oct 1 2021;4(10):e2130645. doi:10.1001/jamanetworkopen.2021.30645

28. Barrios H, Narciso S, Guerreiro M, Maroco J, Logsdon R, de Mendonca A. Quality of life in patients with mild cognitive impairment. *Aging Ment Health*. 2013;17(3):287-92. doi:10.1080/13607863.2012.747083

29. Iwashyna TJ, Ely EW, Smith DM, Langa KM. Long-term cognitive impairment and functional disability among survivors of severe sepsis. *JAMA*. Oct 27 2010;304(16):1787-94. doi:10.1001/jama.2010.1553

30. Donovan NJ, Wu Q, Rentz DM, Sperling RA, Marshall GA, Glymour MM. Loneliness, depression and cognitive function in older U.S. adults. *Int J Geriatr Psychiatry*. May 2017;32(5):564-573. doi:10.1002/gps.4495

31. Mazza MG, Palladini M, De Lorenzo R, et al. Persistent psychopathology and neurocognitive impairment in COVID-19 survivors: Effect of inflammatory biomarkers at three-month follow-up. *Brain Behav Immun*. May 2021;94:138-147. doi:10.1016/j.bbi.2021.02.021

32. De Luca P, Camaioni A, Marra P, et al. Effect of Ultra-Micronized Palmitoylethanolamide and Luteolin on Olfaction and Memory in Patients with Long COVID: Results of a Longitudinal Study. *Cells*. Aug 17 2022;11(16)doi:10.3390/cells11162552

33. Cheshire WP, Freeman R, Gibbons CH, et al. Electrodiagnostic assessment of the autonomic nervous system: A consensus statement endorsed by the American Autonomic Society, American Academy of Neurology, and the International Federation of Clinical Neurophysiology. *Clin Neurophysiol*. Feb 2021;132(2):666-682. doi:10.1016/j.clinph.2020.11.024

34. Case definitions for COVID-19 surveillance. Pan American Health Organization. Accessed October 15, 2022. *https://www.paho.org/en/case-definitions-covid-19-surveillance*

35. Soriano JB, Murthy S, Marshall JC, Relan P, Diaz JV. A clinical case definition of post-COVID-19 condition by a Delphi consensus. *Lancet Infect Dis*. Apr 2022;22(4):e102-e107. doi:10.1016/s1473-3099(21)00703-9

36. Farias SET, Mungas D, Reed BR, Cahn-Weiner DA, Jagust WJ, Baynes K, DeCarli C. The measurement of everyday cognition (ECog): scale development and psychometric properties. *Neuropsychology*. 2008;22 4:531-44.

37. Rodakowski J, Golias KW, Reynolds CF, 3rd, Butters MA, Lopez OL, Dew MA, Skidmore ER. Preventing disability in older adults with mild cognitive impairment: A Strategy Training intervention study. *Contemp Clin Trials Commun*. Sep 2019;15:100368. doi:10.1016/j.conctc.2019.100368

38. McCombe N, Ding X, Prasad G, et al. Alzheimer's Disease Assessments Optimized for Diagnostic Accuracy and Administration Time. *IEEE J Transl Eng Health Med*. 2022;10:4900809. doi:10.1109/jtehm.2022.3164806

39. Mace RA, Hopkins SW, Reynolds GO, Vranceanu AM. My Healthy Brain: Rationale and Case Report of a Virtual Group Lifestyle Program Targeting Modifiable Risk Factors for Dementia. *J Clin Psychol Med Settings*. Dec 2022;29(4):818-830. doi:10.1007/s10880-022-09843-2

40. Cella D, Yount S, Rothrock N, et al. The Patient-Reported Outcomes Measurement Information System (PROMIS): progress of an NIH Roadmap cooperative group during its first two years. *Med Care*. May 2007;45(5 Suppl 1):S3-S11. doi:10.1097/01.mlr.0000258615.42478.55

41. Iverson GL, Marsh JM, Connors EJ, Terry DP. Normative Reference Values, Reliability, and Item-Level Symptom Endorsement for the PROMIS® v2.0 Cognitive Function-Short Forms 4a, 6a and 8a. *Arch Clin Neuropsychol*. Oct 13 2021;36(7):1341-1349. doi:10.1093/arclin/acaa128

42. Cella D, Yount S, Rothrock N, et al. The Patient-Reported Outcomes Measurement Information System (PROMIS): progress of an NIH Roadmap cooperative group during its first two years. *Med Care*. May 2007;45(5 Suppl 1):S3-s11. doi:10.1097/01.mlr.0000258615.42478.55

43. Hays RD, Bjorner JB, Revicki DA, Spritzer KL, Cella D. Development of physical and mental health summary scores from the patient-reported outcomes measurement information system (PROMIS) global items. *Qual Life Res*. Sep 2009;18(7):873-80. doi:10.1007/s11136-009-9496-9

44. Ganesh R, Ghosh AK, Nyman MA, et al. PROMIS Scales for Assessment of Persistent Post-COVID Symptoms: A Cross Sectional Study. *J Prim Care Community Health*. Jan-Dec 2021;12:21501327211030413. doi:10.1177/21501327211030413

45. Ameringer S, Elswick RK, Jr., Menzies V, et al. Psychometric Evaluation of the Patient-Reported Outcomes Measurement Information System Fatigue-Short Form Across Diverse Populations. *Nurs Res*. Jul-Aug 2016;65(4):279-89. doi:10.1097/nnr.0000000000000162

46. Cotler J, Holtzman C, Dudun C, Jason LA. A Brief Questionnaire to Assess Post-Exertional Malaise. *Diagnostics (Basel)*. Sep 11 2018;8(3)doi:10.3390/diagnostics8030066

47. Geffen GM, Butterworth P, Geffen LB. Test-retest reliability of a new form of the auditory verbal learning test (AVLT). *Arch Clin Neuropsychol*. Jul 1994;9(4):303-16.

48. Miranda JP, Valencia RR. English and Spanish versions of a memory test: Word-length effects versus spoken-duration effects. *Hispanic Journal of Behavioral Sciences*. May 1997;19(2):171-181. doi:Doi 10.1177/07399863970192005

49. Maj M, D'Elia L, Satz P, et al. Evaluation of two new neuropsychological tests designed to minimize cultural bias in the assessment of HIV-1 seropositive persons: a WHO study. *Arch Clin Neuropsychol*. Mar 1993;8(2):123-35.

50. Alviarez-Schulze V, Cattaneo G, Pachon-Garcia C, Solana-Sanchez J, Tormos JM, Pascual-Leone A, Bartres-Faz D. Validation and Normative Data of the Spanish Version of the Rey Auditory Verbal Learning Test and Associated Long-Term Forgetting Measures in Middle-Aged Adults. *Front Aging Neurosci*. 2022;14:809019. doi:10.3389/fnagi.2022.809019

51. Smith A. *Symbol digit modalities test*. Western psychological services Los Angeles; 1973.

52. Besser L, Kukull W, Knopman DS, et al. Version 3 of the National Alzheimer's Coordinating Center's Uniform Data Set. *Alzheimer Dis Assoc Disord*. Oct-Dec 2018;32(4):351-358. doi:10.1097/wad.0000000000000279

53. Kelland DZ, Lewis RF. The digit vigilance test: reliability, validity, and sensitivity to diazepam. *Archives of Clinical Neuropsychology*. 1996/01/01/ 1996;11(4):339-344. doi:*https://doi.org/10.1016/0887-6177(95)00032-1*

54. Cogstate. Digital Cognitive Assessment. Accessed November 28, 2022. *https://www.cogstate.com/clinical-trials/digital-cognitive-assessment/*

55. Edgar CJ, Siemers E, Maruff P, Petersen RC, Aisen PS, Weiner MW, Albala B. Pilot Evaluation of the Unsupervised, At-Home Cogstate Brief Battery in ADNI-2. *J Alzheimers Dis*. 2021;83(2):915-925. doi:10.3233/jad-210201

56. Maruff P, Thomas E, Cysique L, Brew B, Collie A, Snyder P, Pietrzak RH. Validity of the CogState brief battery: relationship to standardized tests and sensitivity to cognitive impairment in mild traumatic brain injury, schizophrenia, and AIDS dementia complex. *Arch Clin Neuropsychol*. Mar 2009;24(2):165-78. doi:10.1093/arclin/acp010

57. Zelazo PD, Anderson JE, Richler J, et al. NIH Toolbox Cognition Battery (CB): validation of executive function measures in adults. *J Int Neuropsychol Soc*. Jul 2014;20(6):620-9. doi:10.1017/S1355617714000472

58. Proschan MA, Waclawiw MA. Practical guidelines for multiplicity adjustment in clinical trials. *Control Clin Trials*. Dec 2000;21(6):527-39. doi:10.1016/s0197-2456(00)00106-9

59. Turner GR, Novakovic-Agopian T, Kornblith E, Adnan A, Madore M, Chen AJW, D'Esposito M. Goal-Oriented Attention Self-Regulation (GOALS) training in older adults. *Aging Ment Health*. Mar 2020;24(3):464-473. doi:10.1080/13607863.2018.1534080

60. Novakovic-Agopian T, Posecion L, Kornblith E, et al. Goal-Oriented Attention Self-Regulation Training Improves Executive Functioning in Veterans with Post-Traumatic Stress Disorder and Mild Traumatic Brain Injury. *J Neurotrauma*. Mar 2021;38(5):582-592. doi:10.1089/neu.2019.6806

61. Loya F, Novakovic-Agopian T, Binder D, Rossi A, Rome S, Murphy M, Chen AJ. Long-Term Use and Perceived Benefits of Goal-Oriented Attentional Self-Regulation Training in Chronic Brain Injury. *Rehabil Res Pract*. 2017;2017:8379347. doi:10.1155/2017/8379347

62. Adnan A, Chen AJW, Novakovic-Agopian T, D'Esposito M, Turner GR. Brain Changes Following Executive Control Training in Older Adults. *Neurorehabil Neural Repair*. Oct-Nov 2017;31(10-11):910-922. doi:10.1177/1545968317728580

63. Pilloni G, Vogel-Eyny A, Lustberg M, et al. Tolerability and feasibility of at-home remotely supervised transcranial direct current stimulation (RS-tDCS): Single-center evidence from 6,779 sessions. *Brain Stimul*. May-Jun 2022;15(3):707-716. doi:10.1016/j.brs.2022.04.014

64. Pilloni G, Woods AJ, Charvet L. No risk of skin lesion or burn with transcranial direct current stimulation (tDCS) using standardized protocols. *Brain Stimul*. May-Jun 2021;14(3):511-512. doi:10.1016/j.brs.2021.03.006

65. Muccio M, Walton Masters L, Pilloni G, et al. Cerebral metabolic rate of oxygen (CMRO(2)) changes measured with simultaneous tDCS-MRI in healthy adults. *Brain Res*. Dec 1 2022;1796:148097. doi:10.1016/j.brainres.2022.148097

66. Choi C. Transcranial Direct Current Stimulation (TDCS) Induces Acute Changes in Brain Metabolism. *AAN Enterprises*. 2019;(S33.004)

67. Choe J, Coffman BA, Bergstedt DT, Ziegler MD, Phillips ME. Transcranial Direct Current Stimulation Modulates Neuronal Activity and Learning in Pilot Training. *Front Hum Neurosci*. 2016;10:34. doi:10.3389/fnhum.2016.00034

68. Brunoni AR, Vanderhasselt MA. Working memory improvement with non-invasive brain stimulation of the dorsolateral prefrontal cortex: a systematic review and meta-analysis. *Brain Cogn*. Apr 2014;86:1-9. doi:10.1016/j.bandc.2014.01.008

69. McIntire LK, McKinley RA, Goodyear C, Nelson J. A comparison of the effects of transcranial direct current stimulation and caffeine on vigilance and cognitive performance during extended wakefulness. *Brain Stimul*. Jul-Aug 2014;7(4):499-507. doi:10.1016/j.brs.2014.04.008

70. Elmasry J, Loo C, Martin D. A systematic review of transcranial electrical stimulation combined with cognitive training. *Restor Neurol Neurosci*. 2015;33(3):263-78. doi:10.3233/RNN-140473

71. Gill J, Shah-Basak PP, Hamilton R. It's the thought that counts: examining the task-dependent effects of transcranial direct current stimulation on executive function. *Brain Stimul*. Mar-Apr 2015;8(2):253-9. doi:10.1016/j.brs.2014.10.018

72. Charvet L. Cognitive Functioning in MS Improves with At -Home Online Training Paired with Transcranial Direct Current Stimulation (tDCS): Results f rom a Sham-Controlled Randomized Clinical Trial. ACTRIMS. *https://www.abstractsonline.com/pp8/#!/10495/presentation/67*

73. Smith GE, Housen P, Yaffe K, Ruff R, Kennison RF, Mahncke HW, Zelinski EM. A cognitive training program based on principles of brain plasticity: results from the Improvement in Memory with Plasticity-based Adaptive Cognitive Training (IMPACT) study. *J Am Geriatr Soc*. Apr 2009;57(4):594-603. doi:10.1111/j.1532-5415.2008.02167.x

74. Bikson M, Grossman P, Thomas C, et al. Safety of Transcranial Direct Current Stimulation: Evidence Based Update 2016. *Brain Stimul*. Sep-Oct 2016;9(5):641-661. doi:10.1016/j.brs.2016.06.004

75. Novakovic-Agopian T, Kornblith E, Abrams G, Burciaga-Rosales J, Loya F, D'Esposito M, Chen AJW. Training in Goal-Oriented Attention Self-Regulation Improves Executive Functioning in Veterans with Chronic Traumatic Brain Injury. *J Neurotrauma*. Dec 1 2018;35(23):2784-2795. doi:10.1089/neu.2017.5529

13 APPENDIX A

This intervention is registered on clinicaltrials.gov as NCT05965739.

13.1 INTERVENTION RATIONALE

The interventions below comprise this appendix.

13.1.1 BRAINHQ

BrainHQ is an online cognitive training program developed by Posit Science Corporation, and has been used to improve cognitive function among persons with cognitive impairment based on principles of neuroplasticity. It targets memory, attention, and brain speed. BrainHQ has 29 training exercises and a total of 800 training levels. Exercises focus on speed and accuracy of neural information processing with a reward system when exercises are performed correctly. BrainHQ is a fully remote training system delivered through an application on an electronic device, such as a tablet or computer.

BrainHQ is established for research use and has been used in brain-injured populations. It provides a portal that research teams can use to specialize the challenges that are presented to participants. BrainHQ continuously challenges participants by using algorithms that adapt to each participant's initial cognitive impairment and learning rate. Moreover, BrainHQ exercises are visually appealing and responsive in order to maintain user engagement.

BrainHQ platform provides a set of cognitive activities, like puzzles and games, that are cognitively stimulating and actively engage participants but do not continuously and adaptively challenge them. These activities are designed to be a face-valid, active comparison approach to cognitive therapy, thus participants are blinded, attention time is matched, and overall user experience is identical to the active arms.

13.1.2 PASC-COGNITIVE RECOVERY

PASC-Cognitive Recovery (PASC-CoRE) is a manualized cognitive rehabilitation intervention with demonstrated efficacy in improving attention and executive functions, among other cognitive domains.[59-61] PASC-CoRE comprises 3 essential training components: 1) attention regulation using a mindfulness-based approach; 2) training in goal management; and 3) managing cognitive fatigue.

Attention regulation training uses mindfulness to emphasize redirection of cognitive processes towards tasks that are salient. Participants are taught to use these principles in a range of personally relevant situations. Training takes place virtually through one-on-one or group sessions. Additionally, homework is assigned for more practice in applying these attention-regulation principles. Goal management training encourages participants to identify, select, and execute self-generated, personally relevant, and functional goals.

Fatigue management training teaches participants strategies for managing cognitive fatigue, including "brain breaks," planning ahead, and avoiding overstimulation.

PASC-CoRE is cognitive rehabilitation targeting improvements in executive function and attention. It is a therapy based on an understanding of brain-behavior relationships and may even be capable of inducing neuroplasticity-related brain changes.[62]

13.1.3 TRANSCRANIAL DIRECT CURRENT STIMULATION

Transcranial direct current stimulation (tDCS) will use a device developed by Soterix Medical, Inc. specifically for home-based use (Soterix 1x1 mini-CT). This device delivers a weak electrical current of 2.0 mA passed through two electrodes placed on the scalp to target the dorsolateral prefrontal cortex region of the brain. The electrodes are single-use for each session and can be attached to a headset by snapping into place.

The device has a user-friendly interface and a large-button keypad, making it is easy to use at home. It has many built-in safety features, and the user can easily abort any session if needed. tDCS is FDA-approved for investigational use in the US and has an excellent safety and tolerability profile (i.e., no serious AE reported in human clinical trials to date).[63,64]

tDCS has demonstrated efficacy for many symptoms in the neuropsychiatric cluster, including cognitive dysfunction, central fatigue, central sensitization, and emotional dysregulation. Possible mechanisms of tDCS include increases in neuronal activity and blood flow.[65,66] When paired with a cognitive training activity, it may potentiate and strengthen the learning process.[67] tDCS targeted to brain regions engaged in the cognitive training activity increases brain activity and the corresponding rate of learning during training, thus enhancing its therapeutic outcomes. Pairing with cognitive training may increase learning and performance outcomes, and targeting the dorsolateral prefrontal cortex may lead specifically to improvement in measures of processing speed.[68-71] Repeated application of tDCS has been reported to improve cognitive function.[72]

13.2 EXCLUSION CRITERIA (APPENDIX LEVEL)

1. Presence of metal objects in the head or neck
2. Skin disorders or skin-sensitive areas near tDCS stimulation locations that would interfere with electrode placement or increase the risk of stimulation-induced damage, at the investigator’s discretion

13.3 STUDY DESIGN

These interventions will comprise a five-arm design implemented over 10 weeks:

1. Active Comparator (video games)
2. BrainHQ
3. BrainHQ + PASC-CoRE
4. BrainHQ + tDCS-active
5. BrainHQ + tDCS-sham

13.3.1 DESIGN RATIONALE

This design seeks to evaluate each intervention relative to the Active Comparator. The BrainHQ (alone) arm is important because the intervention is commercially available, accessible, relatively inexpensive, and does not require trained personnel to administer. The BrainHQ + PASC-CoRE arm and the BrainHQ + tDCS arms are suspected to provide cognitive improvements beyond BrainHQ alone through different mechanisms. Both PASC-CoRE and tDCS have extensive prior use. This design does not control for PASC-CoRE; doing so would require a second control group, which would reduce power for observing differences among the primary comparisons. Further, this design positively compromises study-design purity for reduced patient burden and affords analysis of incremental value of intervention combinations most likely to be implemented clinically.

This study design is unique compared to other ongoing or forthcoming clinical trials: It is the only trial investigating BrainHQ alone or BrainHQ paired with PASC-CoRE in a PASC population. Conversely, this study uniquely complements three trials that are or will be recruiting to investigate tDCS with or without cognitive training in patients with PASC. Table 3 summarizes these studies primary outcomes and interventions.

Table 4. Comparison studies for Appendix A

NCT#	Status	Primary Outcome	Intervention Arms	tDCS Dose	Estimated Enrollment
5092516	Recruiting	Change in inhibitory control and processing speed	tDCS-active<br>tDCS-sham	2.0 mA, 4 wk, 7 d/wk, 30 min/d	40
5589272	Not yet recruiting	Change in working memory	tDCS-active + CT (4 wk)<br>tDCS-sham + CT (4 wk)<br>tDCS-active + CT (5d)<br>tDCS-sham + CT (5d)	4 wk: 2.0 mA, 4 wk, 4 d/wk, 20 min/d<br>5 d: 2.0 mA, 5 d consecutive, 13 min/d	60
5389592	Not yet recruiting	Change in neuropsychological assessments of memory, attention, executive functions, and mood	tDCS-active + CT (BrainHQ)<br>tDCS-sham + CT (BrainHQ)	2.0 mA, 4 wk, 5 d/wk, 20 min/d	60

Abbreviations: tDCS, transcranial direct current stimulation; CT, cognitive training

This study complements those in Table 3 because it is investigating BrainHQ alone and is dosing tDCS for a longer duration and session time, while using the same intensity. Further, this study is evaluating neuropsychological changes, though in a larger population. Together, these studies will offer observations on the efficacy of tDCS, particularly tDCS combined with cognitive training, on enhancing cognitive domains in the PASC population.

13.3.2 RANDOMIZATION

Participants will be randomized equally across the five arms, i.e., the randomization probability will be 0.2 for each arm.

13.3.3 BLINDING

Participants, investigators, study personnel, and analysts will be blinded to whether participants are in (a) the BrainHQ or Active Comparator arms and (b) the BrainHQ + tDCS-active and BrainHQ + tDCS-sham arms.

13.3.4 SCHEMA

Figure 2. Schematic of Appendix A intervention

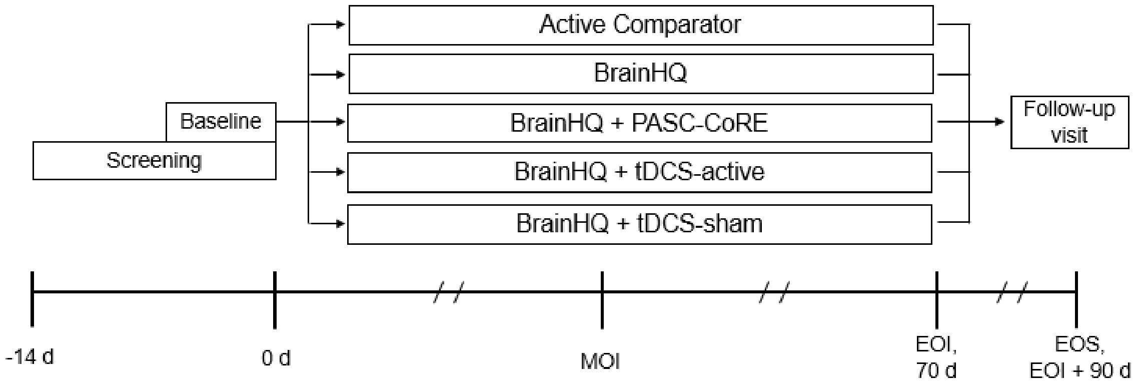

13.3.5 CONTROL/COMPARATOR

The control group is an active comparison group, i.e., not inactive like a waitlist control. The Active Comparator group will receive non-adaptive training or video games, such as word puzzles and visual search games, delivered through the same BrainHQ research portal.

13.4 INTERVENTION PROCEDURES

All intervention procedures will be performed remotely, which requires accessing a device with internet connection. However, limited technology access will not preclude participation. All participants will receive an electronic device preloaded with the relevant BrainHQ configuration and videoconferencing software. Participants without internet access will be provided a hotspot. Additionally, participants in the tDCS groups will be mailed the necessary hardware (see Section 13.4.3). After the intervention, participants will return all materials.

13.4.1 BRAINHQ

BrainHQ and Active Comparator sessions will number 50 over the 10-week period at 5 sessions/week and 30 min/session. In each of the four active intervention arms using BrainHQ, participants will receive active cognitive training. The BrainHQ research portal will be configured for the active cognitive training condition (BrainHQ arms), or the control cognitive training condition (Active Comparator arm), in advance of delivering the device to participants.

Before and after each BrainHQ or Active Comparator session, participants will be prompted to complete a questionnaire, which will document session completion and highlight session interruptions.

Participants will be monitored by a study technician during the entirety of the first 3 sessions. Afterward, participants will be briefly monitored, at startup and the initial few minutes.

13.4.2 PASC-COGNITIVE RECOVERY

PASC-CoRE components will be delivered by trained interventionists during weekly virtual sessions, totaling nine 1.5-hour group sessions and three 30-minute individual sessions (see Table 4 for session content). Individual sessions will occur at the beginning, middle, and end of training series to allow for group session review and one-on-one participant feedback. Group sessions will include two to five participants and one interventionist. The small groups will empower participants to be more active and afford easier scheduling. All sessions will be scheduled to maximize convenience for participants, and will include evenings and weekends. Further, participants will only be required to move through the sessions by scheduled sequence, but they will not be restricted to the same group of participants.

A training manual divided by session will be mailed to participants, who will be instructed to open each session-specific manual at the beginning of the relevant session by the session interventionist. Participants will receive an overview of materials and training in Session #1.

Table 5. PASC-CoRE session content

Session	Content
Group #1	Introduction and overview
Group #2	Progressive information maintenance and mindfulness exercises
Individual #1	*Individualized assessment and strategy building*
Group #3	Goal selection: options for group and individual projects
Group #4	Breaking down projects into sub-tasks, creating timeline; apply to group and individual projects
Group #5	Execution and dealing with procrastination
Individual #2	*Individualized progress monitoring*
Group #6	Staying on tasks, error correction, and adjustments
Group #7	Project progress review and adjustments
Group #8	Individual project presentation
Group #9	Group project presentation, discussion of maintenance, and graduation celebration
Individual #3	*Individual wrap-up*

13.4.3 TRANSCRANIAL DIRECT CURRENT STIMULATION

tDCS devices, headgear, and electrodes displayed in Figure 3 will be mailed directly to participants. tDCS devices used in the active arm will be pre-programmed to deliver the direct electrical current at 2.0 mA for 30 minutes. tDCS devices used in the sham arm will be pre-programmed to deliver the same ramp up/down at the beginning/end of the 30-minute period as the active arm, except with no current

otherwise delivered during the session. For both arms, time of day and consistency of electrode placement will be held as constant as possible; however, given the real-world nature of this study, flexibility will be allowed.

To begin each live online session, a study technician will check for safety and visually confirm correct headset placement and adequate contact quality. Then, the study technician will confirm that the participant is ready to start the session and guide the participant to unlock the device using the one-time use unlock code. At the end of each session, participants will dispose of the single-use electrodes. The study technician will monitor the entire tDCS session for compliance at each daily session during the first week; thereafter, only one session per week will be entirely monitored, while the other four will be briefly monitored for headset placement and contact quality.

tDCS use will be paired with BrainHQ engagement. Participants will engage BrainHQ and then position and power on the headgear. After 30 minutes, participants will disengage from BrainHQ and remove the headgear.

After the intervention, participants will return the devices and headgear in a prepaid container that came with the original package.

Figure 3. Equipment for tDCS intervention

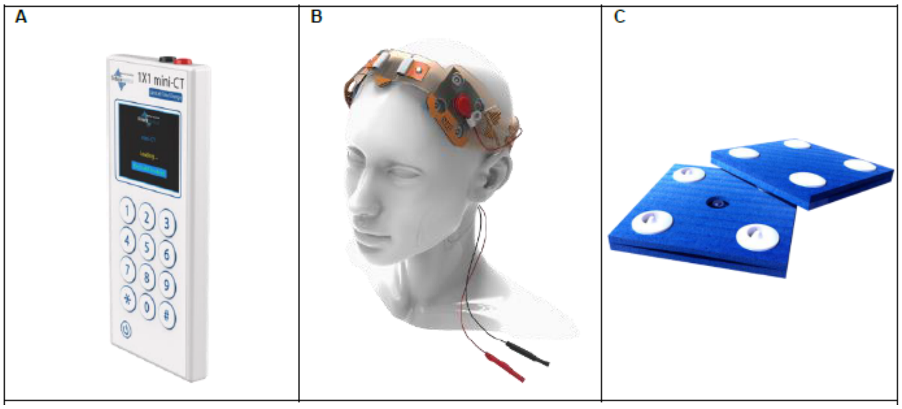

(A) 1x1 Mini-CT tDCS device; (B) SNAPstrap Headgear; (C) EASYpad Electrodes.

13.4.4 DOSING

In summary, the dosing of these intervention arms are as follows:

- Active Comparator: 5 sessions/week at 30 min/session
- BrainHQ: 5 sessions/week at 30 min/session
- BrainHQ + PASC-CoRE: BrainHQ plus 9 group sessions at 1.5 hr/session and 3 individual sessions at 30 min/session
- BrainHQ + tDCS-active: 2.0 mA stimulation delivered for 30 min during each BrainHQ session
- BrainHQ + tDCS-sham: inactive stimulation delivered for 30 min during each BrainHQ session

13.4.5 SCHEDULE OF PROCEDURES (APPENDIX-LEVEL DIFFERENCES)

Clinical laboratory assessments will not be required as part of Baseline assessments, in contrast to Section 8.8.

13.4.6 ADHERENCE

High adherence is expected given the remote nature of the intervention and the study team-participant engagement. Further, tDCS sessions will be monitored regularly; adherence will be captured by the device per session completion. Moreover, the study's cloud database will alert participants when they are to complete their daily session. Finally, the study team will provide remote computer support and technical assistance to ease any technical barriers participants may encounter. Although, participants are encouraged to complete every BrainHQ and tDCS session, one 3-day pause is allowed and will not trigger non-adherence.

13.5 SAFETY

BrainHQ and PASC-CoRE have no obvious risks and have been designated non-significant risk devices by the FDA. Minor AEs associated with BrainHQ are wrist soreness and headache.[73]

tDCS has also been designated non-significant risk with no known risk of SAEs, though common side effects include warmth, itching, or tingling under the electrodes. tDCS will be remotely supervised and within safety limits established by previous research. The tDCS device affords dose and usage control by employing a one-time use-code provided by the study team to unlock the device for each stimulation session (i.e., the device cannot be used outside this window). Additional built-in safety features include automatic shutoff and a manual abort button. Furthermore, the study team trains participants on headgear placement and overall device use. Importantly, tDCS poses no risk of worsening headache in patients with migraines and is not associated with seizure activity in patients with epilepsy.[74]

13.6 EVENTS OF SPECIAL INTEREST

None.

13.7 PREGNANCY AND LIFESTYLE CONSIDERATIONS

Contraception is *not* required.

Relative to the reporting requirements in Section 9.1.6, the following are altered: (a) Pregnancies occurring after starting intervention activities and while on-study will be documented in the database, but pregnant participants will *not* be advised to discontinue the intervention; (b) mothers who conceived a child with a male participant on-study will *not* be required to consent to having their pregnancies followed, and their pregnancies will not be followed through completion; and (c) the DCRI Safety Surveillance team will *not* need to notify device supplying partners of the pregnancy.

Related to these alterations: Pregnancy testing is *not* required at each study visit; thus, participants' self-report is acceptable. Additionally, pregnancy testing is *not* required for female participants who self-

report menopause, have no menstrual cycle for at least 12 months, or are surgically sterile (such as hysterectomy, bilateral tubal ligation, bilateral oophorectomy).

13.8 STATISTICAL CONSIDERATIONS (APPENDIX LEVEL)

13.8.1 SAMPLE SIZE DETERMINATION

No existing published data are available to evaluate the minimal clinically meaningful effect size in the PASC population. As a reference, a study of 33 patients with traumatic brain injury who were randomized to 5 weeks of an intervention using similar approaches to PASC-CoRE versus 5 weeks Brain Health Education found improvements with effect sizes of 0.48 to 0.71 on the Mayo-Portland Adaptability Inventory-4.[75]

A sample size of 50 participants per group will provide 90% power to detect an effect size of 0.655 for a comparison of any two arms, using a two-sample t-test and assuming 1:1 randomization and a two-sided type I error rate of 0.05. Assuming 20% loss to follow-up, 63 participants per arm would need to be randomized. Table 6 provides effect sizes that can be detected with 80 and 90% power under various assumptions for sample size per arm and percent loss to follow-up.

Table 6. Effect sizes that can be detected with 80% and 90% power under various scenarios for enrollment and loss to follow-up

Power	Number of patients per arm	Number of patients needed per arm assuming 10% loss to follow up	Number of patients needed per arm assuming 20% loss to follow up	Effect size
80%	50	56	63	0.57
80%	75	83	94	0.46
80%	100	111	125	0.40
90%	50	56	63	0.66
90%	75	83	94	0.53
90%	100	111	125	0.46

13.8.2 PRIMARY AND SECONDARY COMPARISONS

The current interventions and design afford evaluation of the following primary comparisons:

A. BrainHQ versus Active Comparator
B. BrainHQ + PASC-CoRE versus Active Comparator
C. BrainHQ + tDCS-active versus BrainHQ + tDCS-sham
D. BrainHQ + PASC-CoRE versus BrainHQ

The following secondary comparisons will be evaluated:

E. BrainHQ + tDCS-active versus Active Comparator
F. BrainHQ + tDCS-active versus BrainHQ

A Platform Protocol for Evaluation of Interventions for Cognitive Dysfunction in Post-Acute Sequelae of SARS-CoV-2 Infection (PASC)

Protocol: RECOVER-NEURO (Appendix A)

STATISTICAL ANALYSIS PLAN (SAP)

Protocol Version 3.0

Protocol Version Date: 10 JAN 2024

SAP Version Number: 1.0

13 FEB 2025

Daniel Laskowitz, MD, MHS

Professor of Neurology

Duke Clinical Research Institute

227B Bryan Res Bldg

Durham, NC 27710

Tel: 919-684-0056

E-mail: Daniel.laskowitz@duke.edu

SAP Approval Page

Study Title: A Platform Protocol for Evaluation of Interventions for Cognitive Dysfunction in Post-Acute Sequelae of SARS-CoV-2 Infection (PASC)

Version: 1.0

Date of Issue: 13 FEB 2025

Study Sponsor: Daniel Laskowitz, MD, MHS

Professor of Neurology

Duke Clinical Research Institute

227B Bryan Res Bldg/ Durham, NC 27710

We, the undersigned, have read and approve of this SAP and agree on its content.

Role	Name	Date	Signature
Protocol Statistician	Yuliya Lokhnygina, PhD		
Co-Principal Investigator	David Knopman, MD		
Co-Principal Investigator	Deborah Koltai, PhD		
Co-Principal Investigator, Sponsor	Daniel Laskowitz, MD, MHS		

TABLE OF CONTENTS

1.0 Study and Document Overview 7

2.0 Study Objectives and Estimands 9

3.0 Power and Sample Size 10

3.1 Randomization Scheme 11

3.2 Interim Analyses and Data Monitoring 11

3.2.1 Interim Safety Analyses 11

3.2.2 Interim Analyses for Futility and Efficacy Review 11

3.3 Data Sources 12

3.4 Documentation Convention 12

3.5 Verification of Results 13

3.6 Participant Disposition 13

3.7 Populations for Analyses 13

4.0 Statistical Analyses 14

4.1 General Approach 14

4.2 Analysis of the Primary and Secondary Endpoints 14

4.2.1 Tipping Point Sensitivity Analyses for the Primary Endpoint 18

4.2.2 Inverse Probability Weighted (IPW) Sensitivity Analysis for the Primary Endpoint 21

4.2.3 Alternate Population Sensitivity Analyses for the Primary Endpoint 21

4.2.4 Assessments of Treatment-by-Site Interaction 21

4.3 Safety Analyses 21

4.3.1 Laboratory Data 21

4.3.2 Vital Signs 22

4.3.3 Prior and Comcomitant Medications and Therapies 22

4.3.4 Treatment Compliance 22

4.3.5 Pregnancy Tests 22

4.4 Baseline Descriptive Statistics 23

4.5 Subgroup Analyses 23

4.6 Subjective Global Assessment Questionnaire 24

4.7 Appendices 25

4.7.1 APPENDIX 1. RECOVER-NEURO Roster of Statisticians 25

List of Abbreviations

ADaM	Analysis Data Model
AE	Adverse Event
AVLT	Auditory Verbal Learning Test
CDISC	Clinical Data Interchange Standards Consortium
COVID-19	Coronavirus
DCRI	Duke Clinical Research Institute
DSMB	Data and Safety Monitoring Board
DSQ-PEM	DePaul Symptom Questionnaire – Post Exertional Malaise
ECog2	Everyday Cognition 2
eCRF	Electronic Case Report Form
EOI	End of Intervention
EOS	End of Study
ESI	Event of Special Interest
ET	Early Termination
FU	Follow-up
ITT	Intention-to-Treat
MAR	Missing At Random
MDMR	Master Data Management Repository
MedDRA	Medical Dictionary of Regulatory Activities
mITT	Modified Intention-to-Treat
MOI	Middle of Intervention
NIH	National Institutes of Health
PASC	Post-Acute Sequelae of SARS-CoV-2
PRO	Patient Reported Outcome
PROMIS	Patient-reported Outcomes Measurement Information System

PT	Preferred Term
Q1	25th Percentile (1st Quartile)
Q3	75th Percentile (3rd Quartile)
SAE	Serious Adverse Event
SAP	Statistical Analysis Plan
SARS-CoV-2	Severe Acute Respiratory Syndrome Coronavirus 2
SD	Standard Deviation
SDTM	Study Data Tabulation Model
SOP	Standard Operating Procedure
UADE	Unanticipated Adverse Device Effect
UCLA	University of California, Los Angeles
WHO	World Health Organization

1.0 STUDY AND DOCUMENT OVERVIEW

RECOVER-NEURO is a platform protocol evaluating treatment of Post-Acute Sequelae of SARS-CoV-2 Infection (PASC)-mediated cognitive decline in outpatients previously infected with Severe Acute Respiratory Syndrome Coronavirus 2 (SARS-CoV-2). This is a prospective, multi-center, multi-arm, randomized, partially blinded, controlled trial. This trial will recruit adults experiencing PASC symptoms for at least 12 weeks and reporting reduced cognitive function following acute Coronavirus (COVID-19) infection.

This document describes the planned statistical analyses that will be conducted at the completion of the RECOVER-NEURO study in Appendix A. Once the initial un-blinded data review has occurred, only the blinded statistics team members will be allowed to modify the SAP (see **Appendix 1** for the list of blinded and un-blinded statisticians).

The targeted study population will be eligible participants that are at least 18 years of age at the time of enrollment. These participants will have previous suspected, probable, or confirmed SARS-CoV-2 infection, as defined by the Pan American Health Organization. Eligible participants that have suspected or probable SARS-CoV-2 infection will only be enrolled if the infection occurred prior to 05/01/2021 and will be limited to 10% or less of the total sample size. Additional details regarding this inclusion and exclusion criteria are provided in the Appendix A of the RECOVER-NEURO protocol. Eligible participants must have a Patient-reported Outcomes Measurement Information System (PROMIS) Cog T-score < 40 and report cognitive dysfunction symptoms following a SARS-CoV-2 infection that persist for at least 12 weeks and are still present at the time of consent. The eligible participants must be willing, able, and agree to provide informed consent, complete questionnaires and outcome assessments, participate in the treatment protocol, and return for all study visits.

The intervention duration defined in Appendix A is approximately 10 weeks in duration, with 14 days for participant screening and baseline visits preceding intervention, and a 90-day follow-up period after intervention that concludes with an End of Study clinic visit (EOS). Participants may take up to 3 weeks (21 days) from the date of randomization to begin treatment, a delay justified by the logistic needs of forming groups in the "BrainHQ + PASC-CoRE" treatment arm. There will be clinic visits (1) at Screening, (2) at Baseline, (3) at the Start of Intervention (SOI), (4) at the Middle of Intervention (MOI) (± 3 days), (5) at the End of Intervention (EOI) (+ 3 days), and (6) at the End of Study (EOS) (90 days after End of Intervention ± 3 days). At screening, we will collect potential participants' demographics and medical histories. After determining eligibility (i.e., inclusion and exclusion criteria) and reviewing informed consent, participants who agree to participate will have baseline data collected before any treatment begins. These data will include: demographics, medical history, concomitant medications, PROMIS-Cog, Everyday Cognition 2 (ECog2), a neurocognitive battery (including WHO and Rey Auditory Verbal Learning Test, Symbol Digit Modalities Test, Verbal Fluency (lexical and semantic), Digit Vigilance Test, CogState tests (Detection, Identification, One Back), and National Institutes of Health (NIH) Toolbox Flanker Inhibitory Control and Attention Test), exploratory Patient Reported Outcomes (PROs: PROMIS-29+2, PROMIS-Fatigue, PROMIS-8a Sleep Related Impairment (SRI) and 8b Sleep Disturbance (SD), PASC Symptom Questionnaire, and DePaul Symptom Questionnaire-Post

Exertional Malaise (DSQ-PEM)), blood and stool collection for biorepository, urine or blood pregnancy test, nasal swab for SARS-CoV-2 rapid antigen test, and a safety assessment. The day after completing the baseline neurocognitive battery, participants will complete a DSQ-PEM with lookback period = “since last visit.” At the start of intervention there will be an additional safety assessment. The DSQ-PEM with 7-day lookback period will be given twice weekly during the intervention, from the Start of Intervention to EOI. The twice weekly DSQ-PEM surveys must be separated by at least 48 hours. At the middle of the intervention period, we will collect data on: concomitant medications, PROMIS-Cog, ECog2, Exploratory PROs, and a safety assessment. The day after MOI visit participants will complete a DSQ-PEM with lookback period = “since last visit.” At the End of Intervention visit, we will collect all of the data that will be collected at the midpoint of the intervention period (including DSQ-PEM with lookback period = “since last visit” the following day) as well as the neurocognitive battery, blood and stool collection for biorepository, and the Subjective Global Assessment Questionnaire. At the End of Study, we will collect all of the same data as the End of Intervention visit.

For participants who discontinue an intervention, but do not withdraw from the study, we will attempt to collect safety data on them for at least 28 days.

This trial has a fixed sample design. The study goal is to determine whether the interventions are effective in cognitive dysfunction in this Post-Acute Sequelae of SARS-CoV-2 Infection (PASC) population.

Safety monitoring will be performed throughout the trial and reviewed by the Data and Safety Monitoring Board (DSMB). The DSMB established for the RECOVER project will have oversight responsibility for the study. In the event that the DSMB recommends to discontinue study activities, enrollment and intervention-specific activities will be temporarily suspended while the NIH and the study Co-Principal Investigators consider the DSMB recommendations prior to making decisions on study continuation or discontinuation.

The effectiveness of the intervention programs (treatments) will be evaluated using the primary endpoint, ECog2. We will compare the change in ECog2 score from baseline to EOI between the different treatment groups. Specifically, the following comparisons will be evaluated:

Primary

A. BrainHQ versus Active Comparator

B. BrainHQ + PASC-CoRE versus Active Comparator

C. BrainHQ + tDCS-active versus BrainHQ + tDCS-sham

D. BrainHQ + PASC-CoRE versus BrainHQ

Secondary

E. BrainHQ + tDCS-active versus Active Comparator

F. BrainHQ + tDCS-active versus BrainHQ.

2.0 STUDY OBJECTIVES AND ESTIMANDS

Primary Objective:

- To evaluate the intervention's effect on cognitive function versus comparator, comparing ECog2 change from baseline to end of intervention in adults with previous suspected, probable, or confirmed SARS-CoV-2 infection and persistent neurologic dysfunction following a SARS-CoV-2 infection.

Key Secondary Objectives:

- This trial is largely exploratory and is anticipated to provide important information for future trials. As a result, none of the secondary objectives are specifically being pre-selected as "key."

Summaries of the questions of interest and descriptions of the endpoint attributes are shown in **Table 1**.

Table 1. Study Objectives and Endpoints.

OBJECTIVES	OUTCOME MEASURES	ENDPOINTS
Primary		
Evaluate the intervention's effect on self-reported cognitive function versus comparator	Everyday Cognition 2 (ECog2)	Change in average(mean) score from baseline to End of Intervention (EOI)
Secondary		
Assess the intervention's effect on cognitive patient-reported outcomes (PROs) versus comparator	PROMIS-cognitive function – short form 8a (PROMIS-Cog)	Change in total score from baseline to EOI and End of Study (EOS), defined as 90 days post-intervention
Compare the intervention's effect on an objective neurocognitive battery versus comparator	• World Health Organization and Rey Auditory Verbal Learning Tests<br>• Symbol Digit Modalities Test<br>• Verbal Fluency (lexical + semantic)<br>• Digit Vigilance Test<br>• Cogstate tests: Detection, Identification, One Back<br>• NIH Toolbox Flanker Inhibitory Control and Attention Test	Change from baseline to EOI and EOS
Evaluate the intervention's durable effect on cognitive function versus comparator	ECog2	Change in average score from baseline to EOS

OBJECTIVES	OUTCOME MEASURES	ENDPOINTS
Characterize the intervention's safety	Serious Adverse Events (SAEs), Unanticipated Adverse Device Effects (UADEs)	Proportion of SAEs and UADEs
Exploratory		
Assess the intervention's effect on exploratory PROs versus comparator	• PASC Symptom Questionnaire<br>• Patient-reported Outcomes Measurement Information System (PROMIS)-29+2<br>• PROMIS-fatigue – short form 10a (PROMIS-Fatigue)<br>• PROMIS-8a sleep related impairment (PROMIS-SRI)<br>• PROMIS-8b sleep disturbance (PROMIS-SD)<br>• Modified DePaul Symptom Questionnaire Post Exertional Malaise (DSQ-PEM)	Change in total score from baseline to EOI and EOS for all endpoints except DSQ-PEM. For DSQ-PEM, change in proportion of participants with PEM from baseline to EOI and EOS.

3.0 POWER AND SAMPLE SIZE

No existing published data are available to evaluate the minimal clinically meaningful effect size in the PASC population. As a reference, a study of 33 participants with traumatic brain injury who were randomized to 5 weeks of PASC-CoRE intervention versus 5 weeks Brain Health Education found improvements with effect sizes of 0.48 to 0.71 on the Mayo-Portland Adaptability Inventory-4.

A sample size of 50 participants per group will provide 90% power to detect an effect size of 0.655 for a comparison of any two arms, using a two-sample t-test and assuming 1:1 randomization and a two-sided type I error rate of 0.05. Assuming 20% loss to follow-up, 63 participants per arm would need to be randomized, for a total of 315 participants. **Table 2** provides effect sizes that can be detected with 80 and 90% power under various assumptions for sample size per arm and percent loss to follow-up.

Table 2. Effect sizes that can be detected with 80% and 90% power under various scenarios for enrollment and loss to follow-up

Power	Number of participants per arm	Number of participants needed per arm assuming 10% loss to follow up	Number of participants needed per arm assuming 20% loss to follow up	Effect size
80%	50	56	63	0.57
80%	75	83	94	0.46
80%	100	111	125	0.40

90%	50	56	63	0.66
90%	75	83	94	0.53
90%	100	111	125	0.46

3.1 RANDOMIZATION SCHEME

Randomization across the 5 arms will follow a uniform distribution, that is, the randomization probability equals 0.2 for a participant being assigned to each of the individual arms. The randomization is not stratified. Appendix A will not share any comparator arm participants with any future appendices.

3.2 INTERIM ANALYSES AND DATA MONITORING

A complete description of the planned interim analyses, including mock tables, listings, and figures, is included in a separate DSMB guidance document. Briefly, the DSMB will monitor participant safety as well as review performance of the trial. Participant recruitment, compliance with the study protocol, status of data collection, and other factors will all be monitored regularly.

3.2.1 INTERIM SAFETY ANALYSES

Safety analyses will evaluate SAEs and UADEs by treatment arm. Safety monitoring will be ongoing. Collection of SAEs begins at first treatment received and ends at EOS. Collection of UADEs begins at first treatment received and ends at EOI. BrainHQ, PASC-CoRE, and tDCS have all been designated non-significant risk devices by the FDA. As a result, any SAE which is suspected to be related to treatment will be considered a UADE. The un-blinded statistical team will prepare these interim reports for review by the DSMB.

Generally, AEs that are classified as symptoms associated with PASC and collected during the study will not be collected as a safety event in the study database or further assessed by the site or study personnel because they will be collected as part of the PASC symptom dataset. However, in the event that a non-serious AE is the reason for a participant discontinuing treatment or withdrawing from the trial, the AE will be collected as a safety event.

A more detailed description of interim safety analyses is specified in the DSMB Statistical Analysis Plan.

3.2.2 INTERIM ANALYSES FOR FUTILITY AND EFFICACY REVIEW

There are no planned early stopping rules for efficacy or futility. Because PASC presentations and outcomes are highly varied, an important study objective is to estimate the effect of treatment on a wide range of participant-relevant outcomes. If the study were to be stopped early with less than the full sample size, it would decrease precision and reduce the study's ability to characterize treatment risks and benefits based on

important secondary effectiveness and safety outcomes. It would also limit the collection of data that are critical for planning future trials in similar patient populations.

3.3 DATA SOURCES

At each study site, data will be entered on the electronic Case Report Forms (eCRFs) stored in Medidata Rave. Participants will complete PRO surveys remotely using a supplied tablet computer to access Medidata Rave. The eCRF compliance forms will be filled out by NYU (tDCS and BrainHQ compliance) and by Mount Sinai (PASC-CoRE compliance). First, NYU and Mount Sinai will complete the eCRF related to treatment compliance. Next, the sites will fill out separate treatment compliance forms using the NYU/Mount Sinai treatment compliance forms to inform them. As a result, any compliance analysis will use the NYU/Mount Sinai treatment compliance forms.

Participants in this trial will use the CogState system in order to take CogState neurocognitive tests. CogState will generate an 8-digit code which will be entered into the eCRF and used to link participant CogState records back to the individuals associated with them on the CogState system. Periodically DCRI Data Solutions will load the CogState data directly from CogState and update it on the MDMR.

For the purpose of subgroup analysis, normative data associated with Neurocognitive Battery testing will be used. The original data will come from eCRF data via DCRI Data Solutions. Next, Deborah Koltai will manually calculate z-scores associated with each Neurocognitive Battery test of interest. Finally, this data will then be sent to DCRI Biostatistics and DCRI Project Leader for verification. Once the data is checked (some tests will have 100% records manually checked, some tests will have a fraction manually spot-checked) this data will be manually placed into the necessary directory for subgroup analysis by DCRI Biostatistics.

Prior to database lock, programmed computer edit checks will be run against the database to identify discrepancies and verify reasonableness of the data. Queries to resolve discrepancies will be generated and resolved by the sites. As needed, DCRI Statistics will be able to download the eCRF database and CogState data from the MDMR. DCRI Biostatistics will map and convert raw data to Clinical Data Interchange Standards Consortium Study Data Tabulation Model (CDISC SDTM model version 1.7) datasets. CDISC Analysis Data Model (ADaM model version 2.1) datasets will be created by DCRI Biostatistics for production of final tables, figures, and listings. All planned reporting will be based off of CDISC datasets, but in the case of emergent safety data, some reporting may occur from the raw eCRF data. All programs written to create analysis datasets and perform analyses will be validated according to SOPs established by the DCRI Statistical Programming group.

3.4 DOCUMENTATION CONVENTION

The statistical analyses described in this SAP, as well as production of tables, listings, and figures will be performed using SAS®, version 9.4 or higher (SAS Institute, Cary, NC). Additional statistical software may be used as needed.

3.5 VERIFICATION OF RESULTS

All tables, listings, and figures will be verified and reviewed before considered final. The verification process will ensure that the numbers are produced by a statistically valid method and that the execution of the computations is correct. Qualified statisticians or statistical programmers employed by the DCRI who have not been previously involved in the production of the original programming will perform the verification procedures. Methods of verification include independent programming, prior to issuance of the draft statistical report, of all analysis datasets/ADaM and comparison to data listings. Tables, listings, and figures will be reviewed for accuracy, consistency with this analysis plan, consistency within tables/listings/figures, and consistency with corresponding output. Once verification is complete, all documentation of the verification process will be filed as required by the DCRI SOPs.

3.6 PARTICIPANT DISPOSITION

The disposition of participants (number randomized, number who received any amount of the randomly assigned treatment, number completing study treatment, number who withdrew consent or discontinued from study treatment early, number lost to follow-up, and number who completed the trial) will be summarized by treatment group. Participants will be classified as having completed the trial if the Study Completion form has the question "Did subject complete the study?" = "Yes". Participants will be classified as having completed treatment if they do not have any treatment compliance forms indicating treatment discontinuation and did not discontinue the trial before completing treatment. The number of participants screened for inclusion and a breakdown of reasons for exclusion will be summarized. The timing and reasons for early discontinuation of study treatment and/or withdrawal from the study will be summarized by treatment group and overall. A listing of all participants discontinued from the study after randomization, broken down by site and treatment group will be provided. The listing will include: reason for discontinuation, treatment group, duration of treatment, and whether or not the blind was broken. Also, for participants who discontinued from the study after randomization, a listing of related adverse events will be provided. Participant-specific protocol deviations will be summarized by the reason for the deviation, the deviation category, treatment group, and site for all participants. All major protocol deviations will be counted and summarized by treatment group and as overall.

3.7 POPULATIONS FOR ANALYSES

The primary efficacy analysis will be based on an intent-to-treat (ITT) population, including all participants randomized. The primary safety analyses will be based on a modified intention-to-treat (MITT) population consisting of all participants who received at least 1 intervention activity in the randomization arm to which they were randomized. In the primary efficacy and safety analyses, participants will be analyzed according to their assigned intervention groups.

Additionally, a per-protocol sensitivity analysis of the primary endpoint will be performed. This analysis will only include participants who have met a minimum of 75% treatment compliance among all individual

components of their randomly assigned treatment, do not have any major protocol deviations which could affect treatment efficacy, and will only include them while they remained blinded. If a participant were to become un-blinded prematurely then the participant will not be included in any analysis which uses data after the date of un-blinding.

4.0 STATISTICAL ANALYSES

4.1 GENERAL APPROACH

- Statistical significance: Statistical comparisons will be performed using two-sided significance tests. An alpha level of 0.05 will determine significance, unless otherwise noted.

- Descriptive statistics:
  - Continuous variables will be presented as n, median, Q1, Q3. For comparisons of treatment groups, we will use the t-test or non-parametric Wilcoxon-Mann-Whitney rank-sum test.
  - Categorical variables will be presented as count (percent). Group comparisons will use the conventional chi-square test or Fisher's Exact Test.

- Un-blinding for the final analysis:

  Un-blinding of study data for final analysis will occur after the trial has concluded, the study visits have been monitored, data edits have been completed, queries have been resolved, and the database has been locked. Any necessary modifications or updates to the statistical analysis plan should be made prior to the study un-blinding.

- Handling of Missing Data and Outliers:

  Efforts to minimize loss-to-follow-up will be considerable. However, small amounts of missing data may occur. All attempts will be made to collect all data required per protocol. Any data point that appears to be erroneous or inexplicable based on clinical judgment will be investigated as a possible outlier. If data points are identified as outliers, sensitivity analyses may be performed to examine the impact of including or excluding the outliers. Any substantive differences in these analyses will be reported. Unless stated otherwise stated (i.e. a specific imputation strategy is defined) all analyses are on the complete-case population, only including those participants that are not missing any data required in the analysis.

4.2 ANALYSIS OF THE PRIMARY AND SECONDARY ENDPOINTS

- **Primary Endpoint –** ITT population. Change from baseline to EOI (approximately 70 days later) in Ecog2 score. The Ecog2 score for every participant at each assessment will be calculated as the arithmetic mean of the responses across all of the non-missing and non-"N/A" questionnaire item responses. An Ecog2 score for a particular participant will only be included in analysis if at least 50% of the questions (≥ 21 out of 41) have evaluable responses.

  The primary null hypothesis to be tested is that the mean change from baseline in ECog2 score (Δ Ecog2 = $ECog2_{EOI}$ – $Ecog2_{Baseline}$) does not differ between the 2 treatment groups being compared. The alternative hypothesis is that there is a difference between the treatment groups being compared. This analysis will be adjusted for baseline ECog2 score, age, sex, years of education, and baseline psychological distress. Symbolically the primary hypotheses are:

  H_0: $E[\Delta\ Ecog2]_{Intervention} = E[\Delta\ Ecog2]_{Comparator}$ vs.

  H_A: $E[\Delta\ Ecog2]_{Intervention} \neq E[\Delta\ Ecog2]_{Comparator}$,

  where $E[\Delta\ Ecog2]_{Intervention}$ is the expected (mean) change from baseline Ecog2 for the intervention group, and $E[\Delta\ Ecog2]_{Comparator}$ is the mean change from baseline Ecog2 for the comparator group, conditional on the adjustment covariates. Δ Ecog2 is a negative value when the ECog2 decreases from baseline to end of intervention, which would indicate improvement.

  This analysis will be performed using a linear regression model of the form:

  $Y_i = \beta_0 + \beta_{11}\, x_{11i} + \beta_{12}x_{12i} + \beta_{13}x_{13i} + \beta_{14}x_{14i} + \beta_2 x_{2i} + \beta_3 x_{3i} + \beta_4 x_{4i} + \beta_5 x_{5i} + \beta_6 x_{6i} + \varepsilon_i$

  where Y = Δ Ecog2, and β_j represents the slope and x_{ji} - data values associated with: (1) randomized treatment group (with four indicator variables, x_{11i} - x_{14i}), (2) baseline ECog2 score, (3) age at enrollment, (4) sex, (5) years of education, and (6) baseline psychological distress score. β_0 represents the intercept of the model, in this case the average change in ECog2 from baseline to end of intervention when all other covariates in the model are set to zero. Treatment group, and sex will be entered as categorical variables, and baseline ECog2 score, age at enrollment, years of education, and baseline psychological distress score will be entered as continuous variables. The baseline psychological distress score will be calculated as the mean of the responses on the 8 questions related to Anxiety (#5-8) and Depression (#9-12) on the baseline PROMIS 29+2.

Primary treatment arm comparisons will be:

A. BrainHQ vs. Active Comparator

B. BrainHQ + PASC-CoRE vs. Active Comparator

C. BrainHQ + tDCS-active vs. BrainHQ + tDCS-sham

D. BrainHQ + PASC-CoRE vs. BrainHQ

Secondary comparisons will be:

E. BrainHQ + tDCS-active vs. Active Comparator

F. BrainHQ + tDCS-active vs. BrainHQ

- **Secondary Endpoint –** ITT population. Change in PROMIS-Cog 8a from Baseline to EOI and EOS. These two analyses (change from Baseline to EOI, change from Baseline to EOS) will use the same stated methods as the main primary endpoint analysis. This analysis will use PROMIS-Cog 8a T-Scores.
- **Secondary Endpoints –** ITT population. Change in Neurocognitive Battery from Baseline to EOI and EOS. Using the same stated methods as the main primary endpoint analysis, the analyses will be performed for each of the following in the Neurocognitive Battery. The WHO AVLT will be administered at Baseline and EOS, the Rey AVLT will be administered at EOI. These two versions of the AVLT are comparable, with the only difference being the specific word list. Changing the word list from Baseline (WHO) to EOI (Rey) will minimize learning effect.
  - AVLT (trials #1-5) will generate a score from the total number of correct responses across 5 tests combined.
  - AVLT (trials #8 "delayed recall") will generate a score from the number of correct responses.
  - AVLT (test #9) will generate a score from the number of recognition correct minus false positive.
  - The Symbol Digit Modalities Test score will be the number correct.
  - The Verbal Fluency Lexical score will be the number correct.
  - The Verbal Fluency Semantic score will be the number correct.
  - Digit Vigilance Test will generate 2 scores which will be analyzed separately. One score will be the total time to complete the test, and the other score will be the total errors.

- Cogstate Detection, Cogstate Identification, and Cogstate One Back will be individually analyzed. Each of these 3 tests are administered online, automatically scored, and will generate a total of 4 scores across the 3 tests. The Cogstate Detection score is the mean of $\log_{10}$ transformed reaction times for correct responses. The Cogstate Identification score is mean of $\log_{10}$ transformed reaction times for correct responses. The Cogstate One Back will have a primary score calculated from accuracy $\left(sin^{-1}(\sqrt{proportion\ correct})\right)$ as well as an alternate score generated from speed (mean of $\log_{10}$ transformed reaction times for correct responses).
- Flanker Inhibitory Control and Attention Test - The NIH toolbox will provide a single score for this test which will be entered into the eCRF.

- **Secondary Endpoint –** ITT population. Change in ECog2 from Baseline to EOS. Statistical methods will be the same as is stated for the main primary endpoint analysis (i.e. excluding sensitivity analysis).
- **Secondary Endpoints –** mITT population. Proportion of SAEs and UADEs.
  - The endpoints of SAE and UADE will be analyzed and presented as the number and proportion of participants who experience each specific adverse event, as well as the overall number and proportion of participants who experienced at least one event. The overall number of events will be presented. There are no ESI stated in this protocol appendix.
  - Additionally, the number and proportion of participants that experience an AE, SAE, or UADE that leads to treatment discontinuation will be presented.

- **Exploratory Endpoints** – Exploratory patient reported outcomes including PROMIS-29+2, PROMIS-Fatigue, PROMIS-8a SRI, PROMIS-8b SD, DSQ-PEM, and PASC Symptom Questionnaire will be evaluated at EOI as well as EOS.
  - All PROMIS questionnaires – ITT Population. Each questionnaire will generate individual scores and will be analyzed using the same approach as is stated for the main primary endpoint analysis. PROMIS-29+2 will use the PROPr score, and all other PROMIS questionnaires will use T-Scores.
  - DSQ-PEM – mITT population. The DSQ-PEM will be administered the day after completing the Neurocognitive Battery at Baseline, the day after completing the Middle of Intervention study visit, and the day after completing the Neurocognitive Battery at End of Intervention. Additionally, it will be administered twice weekly throughout the 10-week intervention period. Results of the DSQ-PEM will be tabulated and presented graphically by each intervention arm. The number (%) of participants in each treatment arm who have PEM at

each timepoint will be presented in table format, and the percent of participants with PEM for each treatment arm at the timepoints will be plotted on a line graph.

- PASC Symptom Questionnaire V2.0 – ITT population. Results for the global question, "How would you rate your health right now?" will be summarized with descriptive statistics.

4.2.1 TIPPING POINT SENSITIVITY ANALYSES FOR THE PRIMARY ENDPOINT

Tipping point sensitivity analyses that systematically and comprehensively vary assumptions about the missing outcomes for the five treatment arms will be conducted. These analyses will allow assumptions about the missing primary outcomes for the pair of arms being compared to vary independently. The goal of these analyses is to explore the plausibility of missing data assumptions under which the conclusions change, i.e., if the primary analysis concludes that one arm is superior to another, under which scenarios for missing data there is no longer evidence of efficacy.

We will first use multiple imputation (via SAS PROC MI, using data from all randomized participants, separately by treatment arm) to generate 25 datasets (1 record/participant) which will have a range of values imputed across the datasets for missing baseline ECog2 or missing end of intervention ECog2 scores, as well as any missing baseline age, sex, years of education, or baseline psychological distress scores. Records with imputed ECog2 values will be flagged, and change in ECog2 from baseline to end of intervention will be calculated. Next, a linear regression model will be run using the change from baseline in ECog2 as the dependent variable, and all of the same independent variables that are included in the main analysis primary endpoint model. The 25 datasets will produce 25 outputs of a linear regression model, with the results being combined using Rubin's rules. For the sake of organization, the 25 datasets described above will be referred to as "Version 5", because they will be the 5th set of datasets out of a total of 9 versions of 25 datasets with imputed data. Versions 1, 2, 3, 4 will have incrementally less favorable assumptions applied to the imputed records, as described below. Versions 6, 7, 8, 9 will have incrementally more favorable assumptions applied to the imputed records.

In order to explore a variety of assumptions concerning participants with missing data, the set of 25 datasets will be altered so that change from baseline within each treatment group varies in ¼ SD increments, where SD will be calculated standard deviation of non-missing changes from baseline Ecog2 scores. Below is a table describing how imputed data will be altered to include more and less favorable assumptions. Note that PROC MI is used once below to generate the 25 datasets in "Version 5" and then all other versions of datasets are modifications to these initially created imputed datasets. We will not produce newly imputed data for each "version" to avoid the possibility that datasets which were designed to have "better" results actually have "worse" results than a neighboring version, purely by random chance.

	Version	Description of imputed records*
Worse ↑	1	Imputed baseline records replaced with the maximum of (imputed baseline – 1 SD, 1). Imputed EOI records replaced with the minimum of (imputed EOI + 1 SD, 5).
	2	Imputed baseline records replaced with the maximum of (imputed baseline – ¾ SD, 1). Imputed EOI records replaced with the minimum of (imputed EOI + ¾ SD, 5).
	3	Imputed baseline records replaced with the maximum of (imputed baseline – ½ SD, 1). Imputed EOI records replaced with the minimum of (imputed EOI + ½ SD, 5).
	4	Imputed baseline records replaced with the maximum of (imputed baseline – ¼ SD, 1). Imputed EOI records replaced with the minimum of (imputed EOI + ¼ SD, 5).
	5	Initial non-altered imputation via PROC MI.
↓ Better	6	Imputed baseline records replaced with the minimum of (imputed baseline + ¼ SD, 5). Imputed EOI records replaced with the maximum of (imputed EOI - ¼ SD, 1).
	7	Imputed baseline records replaced with the minimum of (imputed baseline + ½ SD, 5). Imputed EOI records replaced with the maximum of (imputed EOI - ½ SD, 1).
	8	Imputed baseline records replaced with the minimum of (imputed baseline + ¾ SD, 5). Imputed EOI records replaced with the maximum of (imputed EOI - ¾ SD, 1).
	9	Imputed baseline records replaced with the minimum of (imputed baseline + 1 SD, 5). Imputed EOI records replaced with the maximum of (imputed EOI - 1 SD, 1).

* In the event that a participant has missing data at both timepoints (Baseline and EOI), the modifications in this table will be applied at a rate of 50% to each of the timepoints for that individual, for a sum total of 100% of the modification. For example, Version 4 will use maximum of (imputed baseline – 1/8 SD, 1), and the minimum of (imputed EOI + 1/8 SD, 5) on a participant who is missing both Baseline and EOI. As a result this participant would have the imputed change from baseline in ECog2 score changed by ¼ SD, the same change as for the participants that have only 1 timepoint value imputed.

Overall the process for producing the tipping point analysis will be:

1. Use multiple imputation to generate the 25 datasets as outlined above for “Version 5”. Multiple imputation will be done separately by treatment group, for all 5 treatment groups. Then generate 9 versions of the 25 datasets, as described above.
2. Perform a linear regression as described above for each “Version” and each of the 25 datasets, compiling the results across 25 datasets in each “Version” using Rubin’s Rules to calculate a point estimate of the change associated with treatment group, 95% confidence interval, and p-value for the treatment effect.
3. For each of the 6 primary and secondary pairwise comparisons, the results of the tipping point analyses will be summarized in a 9x9 table, corresponding to various combinations of “Versions” for each treatment group involved in the pairwise comparison (see example table below).

difference*<br>95% CI<br>p-value							BrainHQ				
			← BETTER				Neutral Case<br>MAR	WORSE →			
		+ 1 SD	+ ¾ SD	+ ½ SD	+ ¼ SD			- ¼ SD	- ½ SD	- ¾ SD	- 1 SD
	BETTER ↑	+ 1 SD	x.x<br>(x.x, x.x)<br>p = 0.xxx	x.x<br>(x.x, x.x)<br>p = 0.xxx	x.x<br>(x.x, x.x)<br>p = 0.xxx	x.x<br>(x.x, x.x)<br>p = 0.xxx	x.x<br>(x.x, x.x)<br>p = 0.xxx	x.x<br>(x.x, x.x)<br>p = 0.xxx	x.x<br>(x.x, x.x)<br>p = 0.xxx	x.x<br>(x.x, x.x)<br>p = 0.xxx	x.x<br>(x.x, x.x)<br>p = 0.xxx
		+ ¾ SD	x.x<br>(x.x, x.x)<br>p = 0.xxx	x.x<br>(x.x, x.x)<br>p = 0.xxx	x.x<br>(x.x, x.x)<br>p = 0.xxx	x.x<br>(x.x, x.x)<br>p = 0.xxx	x.x<br>(x.x, x.x)<br>p = 0.xxx	x.x<br>(x.x, x.x)<br>p = 0.xxx	x.x<br>(x.x, x.x)<br>p = 0.xxx	x.x<br>(x.x, x.x)<br>p = 0.xxx	x.x<br>(x.x, x.x)<br>p = 0.xxx
		+ ½ SD	x.x<br>(x.x, x.x)<br>p = 0.xxx	x.x<br>(x.x, x.x)<br>p = 0.xxx	x.x<br>(x.x, x.x)<br>p = 0.xxx	x.x<br>(x.x, x.x)<br>p = 0.xxx	x.x<br>(x.x, x.x)<br>p = 0.xxx	x.x<br>(x.x, x.x)<br>p = 0.xxx	x.x<br>(x.x, x.x)<br>p = 0.xxx	x.x<br>(x.x, x.x)<br>p = 0.xxx	x.x<br>(x.x, x.x)<br>p = 0.xxx
		+ ¼ SD	x.x<br>(x.x, x.x)<br>p = 0.xxx	x.x<br>(x.x, x.x)<br>p = 0.xxx	x.x<br>(x.x, x.x)<br>p = 0.xxx	x.x<br>(x.x, x.x)<br>p = 0.xxx	x.x<br>(x.x, x.x)<br>p = 0.xxx	x.x<br>(x.x, x.x)<br>p = 0.xxx	x.x<br>(x.x, x.x)<br>p = 0.xxx	x.x<br>(x.x, x.x)<br>p = 0.xxx	x.x<br>(x.x, x.x)<br>p = 0.xxx
Active Comparator	Neutral Case MAR		x.x<br>(x.x, x.x)<br>p = 0.xxx	x.x<br>(x.x, x.x)<br>p = 0.xxx	x.x<br>(x.x, x.x)<br>p = 0.xxx	x.x<br>(x.x, x.x)<br>p = 0.xxx	x.x<br>(x.x, x.x)<br>p = 0.xxx	x.x<br>(x.x, x.x)<br>p = 0.xxx	x.x<br>(x.x, x.x)<br>p = 0.xxx	x.x<br>(x.x, x.x)<br>p = 0.xxx	x.x<br>(x.x, x.x)<br>p = 0.xxx
	↓ WORSE	- ¼ SD	x.x<br>(x.x, x.x)<br>p = 0.xxx	x.x<br>(x.x, x.x)<br>p = 0.xxx	x.x<br>(x.x, x.x)<br>p = 0.xxx	x.x<br>(x.x, x.x)<br>p = 0.xxx	x.x<br>(x.x, x.x)<br>p = 0.xxx	x.x<br>(x.x, x.x)<br>p = 0.xxx	x.x<br>(x.x, x.x)<br>p = 0.xxx	x.x<br>(x.x, x.x)<br>p = 0.xxx	x.x<br>(x.x, x.x)<br>p = 0.xxx
		- ½ SD	x.x<br>(x.x, x.x)<br>p = 0.xxx	x.x<br>(x.x, x.x)<br>p = 0.xxx	x.x<br>(x.x, x.x)<br>p = 0.xxx	x.x<br>(x.x, x.x)<br>p = 0.xxx	x.x<br>(x.x, x.x)<br>p = 0.xxx	x.x<br>(x.x, x.x)<br>p = 0.xxx	x.x<br>(x.x, x.x)<br>p = 0.xxx	x.x<br>(x.x, x.x)<br>p = 0.xxx	x.x<br>(x.x, x.x)<br>p = 0.xxx
		- ¾ SD	x.x<br>(x.x, x.x)<br>p = 0.xxx	x.x<br>(x.x, x.x)<br>p = 0.xxx	x.x<br>(x.x, x.x)<br>p = 0.xxx	x.x<br>(x.x, x.x)<br>p = 0.xxx	x.x<br>(x.x, x.x)<br>p = 0.xxx	x.x<br>(x.x, x.x)<br>p = 0.xxx	x.x<br>(x.x, x.x)<br>p = 0.xxx	x.x<br>(x.x, x.x)<br>p = 0.xxx	x.x<br>(x.x, x.x)<br>p = 0.xxx
		- 1 SD	x.x<br>(x.x, x.x)<br>p = 0.xxx	x.x<br>(x.x, x.x)<br>p = 0.xxx	x.x<br>(x.x, x.x)<br>p = 0.xxx	x.x<br>(x.x, x.x)<br>p = 0.xxx	x.x<br>(x.x, x.x)<br>p = 0.xxx	x.x<br>(x.x, x.x)<br>p = 0.xxx	x.x<br>(x.x, x.x)<br>p = 0.xxx	x.x<br>(x.x, x.x)<br>p = 0.xxx	x.x<br>(x.x, x.x)<br>p = 0.xxx

*the values within each cell will correspond to the estimated difference, 95% CI, and p-value for the particular setting (e.g. Version 9 BrainHQ, Version 5 Active Comparator).

4.2.2 INVERSE PROBABILITY WEIGHTED (IPW) SENSITIVITY ANALYSIS FOR THE PRIMARY ENDPOINT

Another approach to evaluation of the sensitivity of the primary analysis results to missing outcome data will incorporate inverse probability weighting (IPW) into the estimation of the outcome model described in section 4.2. Each participant with non-missing primary endpoint data will receive a weight equal to the inverse of that participant's predicted probability of having non-missing outcome data, estimated using logistic regression model with covariates of randomized treatment, baseline age, sex, years of education, and baseline psychological distress scores ("weights model"). Baseline ECog2 will also be included as a covariate in the weights model if the p-value for this variable in the model is ≤ 0.1; if the p-value is > 0.1, baseline ECog2 will be dropped from the weights model. The required assumptions for this approach to yield valid inferences are: 1) outcomes are missing at random (MAR) and 2) at least one of the two models (the outcome model and missing data model) is correctly specified.

4.2.3 ALTERNATE POPULATION SENSITIVITY ANALYSES FOR THE PRIMARY ENDPOINT

As a supplement to the main analysis, the primary endpoint analysis described in Section 4.2 will be repeated using the per-protocol population and mITT population.

4.2.4 ASSESSMENTS OF TREATMENT-BY-SITE INTERACTION

We will test for an interaction between the treatment effect and the site by running the original model for the primary endpoint with a "treatment group*site" interaction term added to the right side of the regression equation. The p-value associated with the coefficient for this term will inform any conclusions about the difference in treatment effect by site. This will be performed on the ITT population for the 4 primary and 2 secondary pairwise comparisons (A-F).

4.3 SAFETY ANALYSES

BrainHQ, PASC-CoRE, and tDCS have been designated non-significant risk by the FDA. There are no events of special interest for this appendix. Medical occurrences that begin before intervention procedures, but after obtaining informed consent, will not be considered an AE. As stated previously, the endpoints of SAE and UADE will be analyzed and presented as the proportion of participants who experience each specific adverse event, as well as the overall proportion of participants who experienced at least one event. Lastly, the proportion of participants that experience an AE that leads to treatment discontinuation will be presented.

These analyses will be performed on the mITT population.

4.3.1 LABORATORY DATA

Clinical laboratory assessments are not required for this trial.

4.3.2 VITAL SIGNS

Blood pressure (systolic and diastolic), heart rate, and weight will be summarized and tabulated on a Vital Signs table (by treatment group and overall) as continuous variables at Baseline, EOI, and EOS. Blood pressures and heart rate will be presented using a supine measurement separately from a standing measurement. Baseline height will be presented on the Baseline Characteristics table.

4.3.3 PRIOR AND COMCOMITANT MEDICATIONS AND THERAPIES

Concomitant medications will be coded using the WHO Drug Dictionary. The use of prior, concomitant medications and therapies taken, and changes in concomitant medications and therapies during the study will be recorded on the CRFs. The use of concomitant medications and therapies during the study will be summarized by treatment group for the ITT population. The summaries will be repeated for each arm of the trial.

4.3.4 TREATMENT COMPLIANCE

For all compliance analyses described in this section, each participant in the ITT population will be counted once and weighted equally, regardless of whether or not the participant remains active in the trial for the full expected duration. If a participant withdraws from the study or discontinues before completing all treatment then only the portion of follow-up when a participant is actively participating and expected to complete treatment sessions will be counted as “compliant”.

Treatment compliance within each treatment arm (by individual treatment component within each randomized treatment group) will be tabulated, presenting the median (Q1, Q3) compliance rate per arm. For example, patients randomized to “BrainHQ + PASC-CoRE” will have BrainHQ treatment compliance values as well as PASC-CoRE treatment compliance values presented. Finally, the percent of participants whose overall compliance rate is above 75% will be tabulated.

Average treatment compliance for each arm (by individual treatment component) will be presented graphically as a bar chart.

4.3.5 PREGNANCY TESTS

A set of listings of pregnancies and outcomes will be presented.

4.4 BASELINE DESCRIPTIVE STATISTICS

Baseline characteristics will be summarized by arm for the ITT study population. For continuous measures the median (Q1, Q3) will be summarized. Categorical variables will be described by the proportion in each category (with the corresponding sample size). Baseline characteristics will include age, sex, race, ethnicity, years of education, preferred language (English vs. Spanish), supine and standing blood pressures (systolic and diastolic), supine and standing heart rate, height, and medical history. Medical history will include chronic pulmonary disease, asthma, chronic kidney disease, diabetes, prior stroke or TIA, prior heart attack, heart failure, hypertension, coronary artery disease, cardiomyopathy, peripheral vascular disease, cancer, autoimmune disease, immunosuppression, major depression, anxiety disorder, smoking, and vaping.

4.5 SUBGROUP ANALYSES

The following subgroups have been pre-specified for the primary outcome.

- Unimpaired at baseline vs. impaired at baseline on ECog2. Impaired group will include subjects with an average baseline score of "some difficulty" or worse on the ECog2.
- Unimpaired at baseline vs. impaired at baseline on NeuroCognitive Battery, as defined by scoring worse than 1.5 standard deviations below average across at least 2 cognitive domains.
- Sex assigned at birth: Male vs. Female
- With vs. without fatigue: *the Baseline PROMIS 10a Fatigue will be used to categorize those with and without baseline fatigue. Subjects scoring T-score ≥ 60 are 1 standard deviation (10 points) above average in the PROMIS reference population and will be classified as having fatigue.*
- With/without sleep disorder: *the Baseline PROMIS 8a Sleep Impairment and PROMIS 8b Sleep Disturbance will be used to categorize those with and without baseline sleep disorder. Subjects scoring T-score ≥ 60 on either PROMIS questionnaire are 1 standard deviation (10 points) above average in the PROMIS reference population and will be classified as having sleep disorder.*
- Participants experiencing psychological distress vs. not experiencing psychological distress: *the Baseline PROMIS 29+2 will be used to categorize those with either baseline depression or baseline anxiety. Subjects scoring T-score ≥ 60 are 1 standard deviation (10 points) above average in the PROMIS reference population and will be included in the subgroup of interest. Two separate subgroup analyses will be generated: Anxiety vs. No Anxiety, and Depression vs. No Depression.*
- Post Exertional Malaise – Participants in the PEM subgroup will have a score (frequency, severity) of at least (about half the time, moderate) on any of the first 5 questions of the Baseline DSQ-PEM. Participants who have completed the first 5 questions of the Baseline DSQ-PEM and have all (frequency, severity) scores less than (about half the time, moderate) will be in the non-PEM group. If a participant does not answer all of the first 5 questions of

the Baseline DSQ-PEM and does not have any questions answered with at least (about half the time, moderate) for any of the first 5 questions answered then the participant can not be included in this analysis.

- Additionally, the relationship with age vs. outcome will be explored, using a linear model with age as a continuous variable and age*treatment group interaction term.
- Any subgroup with insufficient sample size will not be analyzed. Excluding "Age" analysis, the above subgroups will be analyzed using a linear model similar to the main analysis, but will include a subgroup variable and subgroup*treatment interaction term. Pairwise comparisons of the 3 primary comparisons and 3 secondary comparisons will be evaluated. Interaction p-values will be presented along with adjusted mean change in ECog2 score from Baseline to End of Treatment for each of the individual treatment groups. These analyses will be performed in the ITT population.

4.6 SUBJECTIVE GLOBAL ASSESSMENT QUESTIONNAIRE

Participants complete this 6-question (2x binary response, 2x 5-category response, 2x 4-category response) questionnaire at the End of Intervention and at the End of Study. Results will be tabulated by arm, with a separate table presented at each of the two timepoints.

4.7 APPENDICES

4.7.1 APPENDIX 1. RECOVER-NEURO ROSTER OF STATISTICIANS

Team	Name	Email	Attends DSMB Meetings
Un-blinded Statistical Team (Closed Session DSMB)	Frank Rockhold	frank.rockhold@duke.edu	X
	Steven McNulty	steven.mcnulty@duke.edu	
	Zhen Huang	zhen.huang@duke.edu	X
	Sophia Waymyers	sophia.waymyers@duke.edu	X
	Hayley Nemeth	hayley.nemeth@duke.edu	X
	Michael Proschan	proscham@niaid.nih.gov	X
Blinded Statistical Team (Open Session DSMB)	Sean O'Brien	sean.m.obrien@duke.edu	X
	Adam Silverstein	adam.silverstein@duke.edu	X
	Yuliya Lokhnygina	yuliya.lokhnygina@duke.edu	X
	Dong-Yun Kim	dong-yun.kim@nih.gov	X
