# Supplementary material for: Evaluation of Interventions for Cognitive Symptoms in Long COVID: A Randomized Clinical Trial
Source: JAMA Neurol. 2025 Nov 10;83(1):49–59. doi: 10.1001/jamaneurol.2025.4415 (PMC12603944; doi:10.1001/jamaneurol.2025.4415)
Supplement: Supplement 2. — eTable 1. Inclusion and Exclusion Criteria eTable 2. Modified ECog2 (mECog2) Instrument eTable 3. Exclusions and Nonrandomizations eTable 4. Concomitant Medications eTable 5. Maintenance of Blindedness eTable 6. Additional mECog2 Outcome for Per-Protocol Analysis at End of Intervention (EOI) eTable 7. Additional mECog2 Outcome at End of Study (EOS) eTable 8. Postexertional Malaise Across Treatment Groups eTable 9. Treatment-Emergent Serious Adverse Events eFigure 1. Distribution of mECog2 Scores: ITT Population eFigure 2. Subgroup Analysis Forest Plots: ITT Population [file jamaneurol-e254415-s002.pdf]

Supplementary Online Content

Knopman DS, Koltai D, Laskowitz D, et al; RECOVER-NEURO Clinical Trial Group. Evaluation of interventions for cognitive symptoms in long COVID: a randomized clinical trial. *JAMA Neurol*. Published online November 10, 2025. doi:10.1001/jamaneurol.2025.4415

eTable 1. Inclusion and Exclusion Criteria

eTable 2. Modified ECog2 (mECog2) Instrument

eTable 3. Exclusions and Nonrandomizations

eTable 4. Concomitant Medications

eTable 5. Maintenance of Blindedness

eTable 6. Additional mECog2 Outcome for Per-Protocol Analysis at End of Intervention (EOI)

eTable 7. Additional mECog2 Outcome at End of Study (EOS)

eTable 8. Postexertional Malaise Across Treatment Groups

eTable 9. Treatment-Emergent Serious Adverse Events

eFigure 1. Distribution of mECog2 Scores: ITT Population

eFigure 2. Subgroup Analysis Forest Plots: ITT Population

This supplementary material has been provided by the authors to give readers additional information about their work.

© 2025 Knopman DS et al. *JAMA Neurology*.

eTable 1. Inclusion and Exclusion Criteria

INCLUSION CRITERIA

In order to be eligible to participate in this study, an individual must meet all of the following criteria:

1. ≥ 18 years of age at the time of enrollment
2. PROMIS-Cog T-score < 40
3. Previous suspected, probable, or confirmed SARS-CoV-2 infection, as defined by the Pan American Health Organization

   Suspected case of SARS-CoV-2 infection - three options, A through C:

   A. Met clinical OR epidemiological criteria:
      - *a. Clinical criteria: Acute onset of fever AND cough (influenza-like illness) OR Acute onset of ANY THREE OR MORE of the following signs or symptoms: fever, cough, general, weakness/fatigue, headache, myalgia, sore throat, coryza, dyspnea, nausea, diarrhea, anorexia;*
      - *b. Epidemiological criteria: Contact of a probable or confirmed case or linked to a COVID-19 cluster;*

   B. Presented acute respiratory infection with history of fever or measured fever of ≥ 38°C and cough, with onset within the last 10 days, and who requires hospitalization; or

   C. Presented with no clinical signs or symptoms, NOR meeting epidemiologic criteria with a positive professional use or self-test SARS-CoV-2 Antigen-Rapid Diagnostic Test.

   Probable case of SARS-CoV-2 infection, defined as having met clinical criteria above AND was a contact of a probable or confirmed case or was linked to a COVID-19 cluster.

   Confirmed case of SARS-CoV-2 infection - two options, A through B:

   A. Presented with a positive nucleic acid amplification test, regardless of clinical criteria OR epidemiological criteria; or

   B. Met clinical AND/OR epidemiological criteria (See suspected case A), with a positive professional use or self-test SARS-CoV-2 Antigen-Rapid Diagnostic Test.

   Suspected and probable cases will only be allowed if they occurred before May 1, 2021, and will be limited to 10% of the study population. Otherwise, confirmed cases are required.
4. Cognitive dysfunction symptoms following a SARS-CoV-2 infection that have persisted for at least 12 weeks and are still present at the time of consent
5. Fluent in English or Spanish language
6. Willing and able to provide informed consent, complete the intervention, complete the intervention assessments, and return for all of the necessary follow-up visits

EXCLUSION CRITERIA

An individual who meets any of the following criteria will be excluded from participation in this study:

1. Prior or active unstable or progressive major psychiatric or neurologic condition that would not show improvement and could hide treatment effect, at the investigator’s discretion, including, but not limited to, the following examples:
   - a. Progressive neurodegenerative disease, such as Alzheimer’s disease, Parkinson’s disease, etc.
   - b. Past traumatic brain injury occurrence still associated with active post-concussive symptoms
   - c. Uncontrolled seizure disorder, such as having at least one seizure in the last year that is adjudicated by clinical judgment
   - d. Post-stroke deficits that may interfere with assessment, such as language or communication difficulties, aphasia, etc.
   - e. Formal thought disorders, such as schizophrenia, etc.
   - f. Any neuropsychiatric or neurologic disorder uncontrolled for the previous 6 months or that may interfere with assessment, at discretion of the investigator
2. Known prior diagnosis of myalgic encephalomyelitis/chronic fatigue syndrome, not related to SARS-CoV-2 infection
3. Known active acute SARS-CoV-2 infection ≤ 4 weeks from consent

© 2025 Knopman DS et al. *JAMA Neurology*.

4. Medications as qualified in the Protocol. In overview, stimulants, drugs intended to treat post-acute sequelae of SARS-CoV-2 infection (PASC) symptoms, narcotics, benzodiazepines, and anticholinergics. Antidepressants were allowed if dose had been stable for >90 days prior to randomization.
5. History of electroconvulsive therapy
6. Current use of any medication for treating PASC-related symptoms
7. Attention-deficit/hyperactivity disorder (ADHD) diagnosis following the onset of PASC*
8. Current diagnosis of alcohol and substance use disorders
    a. Prior use disorders acceptable if abstinence achieved and maintained for at least 12 months before study enrollment
9. Insufficient visual, auditory, and motor function to participate in intervention and assessments
10. Known pregnancy
11. Current or recent use (within the last 2 months) of the intervention**
12. Known allergy/sensitivity/hypersensitivity to components of the intervention or comparator**
13. Currently receiving/using intervention from another clinical trial, such as another RECOVER trial***
14. Any condition that would make the participant, in the opinion of the investigator, unsuitable for the study
    a. The site investigator has the discretion to determine whether a participant is too cognitively impaired to participate and should instead be referred for clinical evaluation.

---

* Participants diagnosed with ADHD prior to PASC-related symptoms and who are compliant on stable doses of medication may participate per the investigator's discretion. Participants should agree to maintain a stable dose of their medication while in the trial unless directed by their treating physicians to change the dose.

** Relevant if only one intervention appendix is open at the time of enrollment, though exclusion may be qualified in the appendix. If multiple intervention appendices are open, a participant may be excluded from any intervention appendix based on contraindications listed in the intervention appendix, current use of intervention, or known allergy/sensitivity/hypersensitivity and still remain eligible for the remaining intervention appendices.

*** Following another interventional trial's end-of-study visit, participants must wait 90 days before enrolling in NEURO.

© 2025 Knopman DS et al. *JAMA Neurology*.

eTable 2. Modified ECog2 (mEcog2) Instrument

Revised Everyday Cognition Scale – Self-Report Form *modified*

Directions: Please rate your ability to perform various everyday tasks on a scale of 1 to 5, from having no difficulty (1) to great difficulty/unable (5), focusing on the last 7 days. Circle only one option that best fits your response. Then please also indicate whether this rating reflects a change from how you were doing prior to COVID infection by circling YES or NO.

In the last 7 days…	**No difficulty**	**Little difficulty**	**Some difficulty**	**Much difficulty**	**Great difficulty/ Unable**	**Not Applicable**		**Is this different than before you had COVID?**
**Memory**								
1. Remembering a few shopping items without a list.	1	2	3	4	5	N/A		☐YES ☐NO
2. Remembering things that happened recently (such as outings or news events from the last few weeks).	1	2	3	4	5	N/A		☐YES ☐NO
3. Recalling conversations from a few days ago.	1	2	3	4	5	N/A		☐YES ☐NO
4. Remembering where you have placed personal items or objects.	1	2	3	4	5	N/A		☐YES ☐NO
5. Avoiding telling stories or asking questions multiple times.	1	2	3	4	5	N/A		☐YES ☐NO
6. Remembering the current date or day of the week.	1	2	3	4	5	N/A		☐YES ☐NO

© 2025 Knopman DS et al. *JAMA Neurology*.

7. Recalling that you have already told someone something.	1	2	3	4	5	N/A		☐YES ☐NO
8. Remembering appointments, meetings, or engagements.	1	2	3	4	5	N/A		☐YES ☐NO
9. Remembering to do important tasks like pay bills or take medications.	1	2	3	4	5	N/A		☐YES ☐NO

In the last 7 days…	**No difficulty**	**Little difficulty**	**Some difficulty**	**Much difficulty**	**Great difficulty/ Unable**	**Not applicable**		**Is this different than before COVID?**
**Language**								
1. Coming up with the right names of commonly used everyday objects (e.g., phone, toothbrush).	1	2	3	4	5	N/A		☐YES ☐NO
2. Verbally giving instructions to others.	1	2	3	4	5	N/A		☐YES ☐NO
3. Finding the exact right words to use in a conversation.	1	2	3	4	5	N/A		☐YES ☐NO
4. Communicating thoughts or expressing ideas in a conversation.	1	2	3	4	5	N/A		☐YES ☐NO
5. Following a story in a book or on TV.	1	2	3	4	5	N/A		☐YES ☐NO
6. Understanding the point of what other people are trying to say.	1	2	3	4	5	N/A		☐YES ☐NO
7. Remembering the meaning of common words.	1	2	3	4	5	N/A		☐YES ☐NO
8. Describing a TV show or movie you have watched.	1	2	3	4	5	N/A		☐YES ☐NO

© 2025 Knopman DS et al. *JAMA Neurology*.

9. Understanding spoken directions or instructions.	1	2	3	4	5	N/A	☐YES ☐NO
In the last 7 days…	**No difficulty**	**Little difficulty**	**Some difficulty**	**Much difficulty**	**Great difficulty/ Unable**	**Not applicable**	**Is this different than before COVID?**
**Visual-spatial and Perceptual Abilities**							
1. Finding your way around town without needing to use your GPS.	1	2	3	4	5	N/A	☐YES ☐NO
2. Finding your car in a parking lot.	1	2	3	4	5	N/A	☐YES ☐NO
3. Finding the way to a familiar location (e.g., a long-term friend's home).	1	2	3	4	5	N/A	☐YES ☐NO
4. Finding your way around your own neighborhood.	1	2	3	4	5	N/A	☐YES ☐NO
5. Finding your way around a familiar store or other building.	1	2	3	4	5	N/A	☐YES ☐NO
6. Finding your way around a familiar home.	1	2	3	4	5	N/A	☐YES ☐NO
7. Using landmarks in the environment to find locations (e.g., turn left after the grocery store).	1	2	3	4	5	N/A	☐YES ☐NO
8. Driving with safe/appropriate distances from other vehicles or objects.	1	2	3	4	5	N/A	☐YES ☐NO

© 2025 Knopman DS et al. *JAMA Neurology*.

In the last 7 days…	No difficulty	Little difficulty	Some difficulty	Much difficulty	Great difficulty/ Unable	Not applicable	Is this different than before COVID?
**Executive Functioning: Planning**							
1. Planning the sequence of stops on a shopping trip.	1	2	3	4	5	N/A	☐YES ☐NO
2. Anticipating weather changes and planning accordingly (e.g., bringing a coat or umbrella).	1	2	3	4	5	N/A	☐YES ☐NO
3. Planning and sticking to a schedule for the day when there are several things to be done.	1	2	3	4	5	N/A	☐YES ☐NO
4. Thinking things through before making a major decision (e.g., making a big purchase).	1	2	3	4	5	N/A	☐YES ☐NO
5. Planning a trip, vacation, or outing.	1	2	3	4	5	N/A	☐YES ☐NO
In the last 7 days…	**No difficulty**	**Little difficulty**	**Some difficulty**	**Much difficulty**	**Great difficulty/ Unable**	**Not applicable**	**Is this different than before COVID?**
**Executive Functioning: Organization**							
1. Keeping living and work spaces (office, garage or pantry) organized.	1	2	3	4	5	N/A	☐YES ☐NO
2. Managing bill payments.	1	2	3	4	5	N/A	☐YES ☐NO

© 2025 Knopman DS et al. *JAMA Neurology*.

3. Keeping financial records or other documents organized so they are easy to find when needed.	1	2	3	4	5	N/A	☐YES ☐NO
4. Prioritizing tasks by importance (doing the most important things first).	1	2	3	4	5	N/A	☐YES ☐NO
5. Keeping mail and papers organized.	1	2	3	4	5	N/A	☐YES ☐NO
6. Using an organized strategy to manage a medication schedule involving multiple medications.	1	2	3	4	5	N/A	☐YES ☐NO
In the last 7 days…	**No difficulty**	**Little difficulty**	**Some difficulty**	**Much difficulty**	**Great difficulty/ Unable**	**Not applicable**	**Is this different than before COVID?**
**Executive Functioning: Divided Attention**							
1. Doing two things at once.	1	2	3	4	5	N/A	☐YES ☐NO
2. Returning to a task after being interrupted.	1	2	3	4	5	N/A	☐YES ☐NO
3. Concentrating on and completing a task without being distracted by other things.	1	2	3	4	5	N/A	☐YES ☐NO
4. Working on a particular task while talking to someone at the same time.	1	2	3	4	5	N/A	☐YES ☐NO

© 2025 Knopman DS et al. *JAMA Neurology*.

eTable 3. Exclusions and Nonrandomizations

	All Participants (N=378)
**Number of participants screened for inclusion/exclusion**	378 (100%)
Number of participants who did not meet inclusion OR met exclusion	56 (14.8%)
Number of participants who met inclusion and did not meet exclusion	322 (85.2%)
Number of participants who were randomized	328 (86.8%)
**Met exclusion criteria**	
1. Prior or active unstable or progressive major psychiatric or neurologic condition that would not show improvement and could hide treatment effect, at the investigator's discretion	2 (0.5%)
2. Known prior diagnosis of myalgic encephalomyelitis/chronic fatigue syndrome, not related to SARS-CoV-2 infection	1 (0.3%)
3. Known active acute SARS-CoV-2 infection ≤ 4 weeks from consent	4 (1.1%)
4. Current use of prohibited medications	6 (1.6%)
5. Current use of any medication/stimulant for treating PASC-related symptoms	4 (1.1%)
6. Current diagnosis of alcohol and substance use disorders	1 (0.3%)
7. Insufficient visual, auditory, and motor function to participate in intervention and assessments	1 (0.3%)
8. Known pregnancy	1 (0.3%)
9. Current or recent use (within the last 2 months) of intervention	2 (0.5%)
10. Known allergy/sensitivity/hypersensitivity to components of the intervention or comparator	2 (0.5%)
13. Currently receiving/using intervention from another clinical trial, such as another RECOVER trial	3 (0.8%)
14. Any condition that would make the participant, in the opinion of the investigator, unsuitable for the study	7 (1.9%)
15. Attention-deficit/hyperactivity disorder (ADHD) diagnosis following the onset of PASC	1 (0.3%)
16. History of electroconvulsive therapy	0
17. Presence of metal objects in the head or neck	6 (1.6%)
18. Skin disorders or skin-sensitive areas near transcranial direct current stimulation locations that would interfere with electrode placement or stimulation, at the investigator's discretion	2 (0.5%)
**Did not meet inclusion criteria**	
1. ≥ 18 years of age at enrollment	0
2. PROMIS-Cog T-score < 40	31 (8.2%)
3. Previous suspected, probable, or confirmed SARS-CoV2 infection	1 (0.3%)
4. Cognitive dysfunction symptoms following a SARS-CoV2 infection that have persisted for at least 12 weeks and were still present at the time of consent	2 (0.5%)
5. Fluent in English or Spanish	1 (0.3%)
6. Willing and able to provide informed consent, complete the surveys, clinical assessments, and return for all of the necessary follow-up visits	4 (1.1%)

© 2025 Knopman DS et al. *JAMA Neurology*.

eTable 4. Concomitant Medications

	All Participants (N=328)
Anticholinergic/Antihistamine	34 (10.4%)
loratadine	12 (3.7%)
azelastine	7 (2.1%)
fexofenadine hydrochloride	6 (1.8%)
fexofenadine	4 (1.2%)
diphenhydramine hydrochloride	2 (0.6%)
ketotifen	2 (0.6%)
meclozine	2 (0.6%)
azelastine hydrochloride	1 (0.3%)
azelastine hydrochloride;fluticasone propionate	1 (0.3%)
doxylamine succinate	1 (0.3%)
loratadine;pseudoephedrine sulfate	1 (0.3%)
meclozine hydrochloride	1 (0.3%)
Antidepressants	99 (30.2%)
Ssri/Snri/Ndri	93 (28.4%)
bupropion	14 (4.3%)
sertraline	10 (3.0%)
trazodone	10 (3.0%)
bupropion hydrochloride	8 (2.4%)
duloxetine	8 (2.4%)
sertraline hydrochloride	8 (2.4%)
duloxetine hydrochloride	7 (2.1%)
fluoxetine hydrochloride	7 (2.1%)
fluoxetine	5 (1.5%)
citalopram	4 (1.2%)
escitalopram	4 (1.2%)
venlafaxine hydrochloride	4 (1.2%)
buspirone	3 (0.9%)
citalopram hydrobromide	3 (0.9%)
escitalopram oxalate	3 (0.9%)
venlafaxine	3 (0.9%)
desvenlafaxine	2 (0.6%)
mirtazapine	2 (0.6%)
trazodone hydrochloride	2 (0.6%)
bupropion hcl ;naltrexone hcl	1 (0.3%)
desvenlafaxine succinate monohydrate	1 (0.3%)
esketamine	1 (0.3%)
fluvoxamine	1 (0.3%)
oxitriptan	1 (0.3%)
paroxetine	1 (0.3%)
Tricyclic	7 (2.1%)
amitriptyline	3 (0.9%)
nortriptyline	2 (0.6%)
amitriptyline hydrochloride	1 (0.3%)
imipramine hydrochloride	1 (0.3%)
Anti-psychotics	5 (1.5%)
aripiprazole	2 (0.6%)
quetiapine	2 (0.6%)
lurasidone	1 (0.3%)

© 2025 Knopman DS et al. *JAMA Neurology*.

	All Participants (N=328)
Cannabinoid	6 (1.8%)
cannabidiol	2 (0.6%)
tetrahydrocannabinols NOS	2 (0.6%)
cannabidiol;tetrahydrocannabinols NOS	1 (0.3%)
promethazine	1 (0.3%)
Narcotics	4 (1.2%)
codeine;guaifenesin	1 (0.3%)
hydrocodone bitartrate;paracetamol	1 (0.3%)
hydrocodone;paracetamol	1 (0.3%)
oxycodone	1 (0.3%)
Other agents often used for Long COVID	29 (8.8%)
naltrexone	18 (5.5%)
acetylcysteine	11 (3.4%)
naltrexone hydrochloride	2 (0.6%)
(6s)-5-methyltetrahydrofolate glucosamine;acetylcarnitine hydrochloride;acetylcysteine;ascorbic acid;betacarotene;biotin;brassica oleracea seed;calcium citrate;calcium pantothenate;choline bitartrate;	1 (0.3%)
bupropion hydrochloride;naltrexone hydrochloride	1 (0.3%)
Sedative hypnotics	40 (12.2%)
Anti-spasmodics	19 (5.8%)
cyclobenzaprine	6 (1.8%)
baclofen	5 (1.5%)
methocarbamol	4 (1.2%)
cyclobenzaprine hydrochloride	3 (0.9%)
tizanidine	2 (0.6%)
muscle relaxants	1 (0.3%)
Benzodiazepines and z drugs	28 (8.5%)
alprazolam	12 (3.7%)
clonazepam	4 (1.2%)
lorazepam	4 (1.2%)
zolpidem tartrate	4 (1.2%)
diazepam	2 (0.6%)
eszopiclone	2 (0.6%)
zolpidem	2 (0.6%)
zaleplon	1 (0.3%)
Stimulants	23 (7.0%)
amphetamine based	18 (5.5%)
[amphetamine] aspartate; [amphetamine] sulfate;dexamfetamine saccharate;dexamfetamine sulfate	9 (2.7%)
lisdexamfetamine mesilate	2 (0.6%)
methylphenidate	2 (0.6%)
phentermine	2 (0.6%)
[amphetamine];dexamfetamine	1 (0.3%)
dexamfetamine	1 (0.3%)
methylphenidate hydrochloride	1 (0.3%)
non-amphetamine	7 (2.1%)
guanfacine	4 (1.2%)
amantadine	1 (0.3%)

© 2025 Knopman DS et al. *JAMA Neurology*.

	All Participants (N=328)
guanfacine hydrochloride	1 (0.3%)
phosphatidyl serine	1 (0.3%)

© 2025 Knopman DS et al. *JAMA Neurology*.

eTable 5. Maintenance of Blindedness

	Active Comparator (N=64)	BrainHQ (N=67)	PASC-CoRE + BrainHQ (N=66)	tDCS-active + BrainHQ (N=66)	tDCS-sham + BrainHQ (N=65)
Do you believe you received the active intervention or the active comparator tDCS?					
Active intervention				26 / 55 (47.3%)	22 / 59 (37.3%)
Active comparator				3 / 55 (5.5%)	16 / 59 (27.1%)
I don't know				26 / 55 (47.3%)	21 / 59 (35.6%)
I did not receive this treatment				0 / 55	0 / 59
Do you believe you received the active intervention or the active comparator cognitive training games?					
Active intervention	11 / 61 (18.0%)	23 / 62 (37.1%)	36 / 63 (57.1%)	28 / 55 (50.9%)	36 / 59 (61.0%)
Active comparator	26 / 61 (42.6%)	10 / 62 (16.1%)	4 / 63 (6.3%)	4 / 55 (7.3%)	8 / 59 (13.6%)
I don't know	22 / 61 (36.1%)	26 / 62 (41.9%)	20 / 63 (31.7%)	23 / 55 (41.8%)	14 / 59 (23.7%)
I did not receive this treatment	2 / 61 (3.3%)	3 / 62 (4.8%)	3 / 63 (4.8%)	0 / 55	1 / 59 (1.7%)

© 2025 Knopman DS et al. *JAMA Neurology*.

eTable 6. Additional mECog2 Outcome for Per-Protocol Analysis at End of Intervention (EOI)

	Baseline<br>N<br>Mean[1] (95% CI)	EOI<br>N<br>Mean[1] (95% CI)	Unadjusted Change From Baseline to EOI<br>N<br>Mean (95% CI)	Adjusted Difference in Mean Change vs Active Comparator[2]	Adjusted Difference in Mean Change vs BrainHQ[2]	Adjusted Difference in Mean Change vs tDCS sham + BrainHQ[2]
All (N=277)	276<br>2.8 (2.7, 2.9)	267<br>2.4 (2.3, 2.5)	267<br>-0.4 (-0.5, -0.3)			
Active comparator (N=54)	54<br>2.8 (2.6, 2.9)	53<br>2.4 (2.2, 2.6)	53<br>-0.4 (-0.6, -0.2)			
BrainHQ (N=57)	57<br>2.8 (2.7, 3.0)	56<br>2.4 (2.2, 2.6)	56<br>-0.4 (-0.5, -0.2)	**0.0 (-0.2, 0.2)<br>p = 0.872**		
PASC-CoRE + BrainHQ (N=55)	55<br>2.8 (2.6, 2.9)	55<br>2.6 (2.4, 2.8)	55<br>-0.2 (-0.4, -0.0)	**0.2 (-0.0, 0.4)<br>p = 0.113**	**0.2 (-0.1, 0.4)<br>p = 0.147**	
tDCS-active + BrainHQ (N=54)	54<br>3.0 (2.7, 3.2)	49<br>2.3 (2.1, 2.5)	49<br>-0.5 (-0.7, -0.4)	-0.1 (-0.4, 0.1)<br>p = 0.252	-0.1 (-0.4, 0.1)<br>p = 0.187	**0.0 (-0.2, 0.2)<br>p = 0.906**
tDCS-sham + BrainHQ (N=57)	56<br>2.8 (2.6, 3.0)	54<br>2.3 (2.1, 2.5)	54<br>-0.5 (-0.7, -0.3)			

The per-protocol population includes participants with at least 75% treatment compliance in each of the components of their randomized treatment, and no major protocol deviations which could affect treatment efficacy.

[1] mECog2 has a score from 1 to 5, with 5 being worst.

[2] Negative adjusted difference in mean change indicates the treatment group in the row did better than the treatment group in the column.

© 2025 Knopman DS et al. *JAMA Neurology*.

eTable 7. Additional mECog2 Outcome at End of Study (EOS)

	Baseline N Mean[1] (95% CI)	EOS N Mean[1] (95% CI)	Unadjusted Change From Baseline to EOS N Mean (95% CI)	Adjusted Difference in Mean Change vs Active Comparator[2]	Adjusted Difference in Mean Change vs BrainHQ[2]	Adjusted Difference in Mean Change vs tDCS sham + BrainHQ[2]
All (N=328)	327<br>2.8 (2.7, 2.9)	295<br>2.3 (2.2, 2.4)	295<br>-0.5 (-0.6, -0.4)			
Active comparator (N=64)	64<br>2.8 (2.6, 2.9)	59<br>2.3 (2.1, 2.5)	59<br>-0.5 (-0.6, -0.3)			
BrainHQ (N=67)	67<br>2.8 (2.7, 3.0)	62<br>2.3 (2.1, 2.5)	62<br>-0.5 (-0.7, -0.4)	**0.0 (-0.3, 0.2)**<br>**p = 0.716**		
PASC-CoRE + BrainHQ (N=66)	66<br>2.8 (2.7, 3.0)	62<br>2.4 (2.2, 2.6)	62<br>-0.4 (-0.6, -0.2)	**0.0 (-0.2, 0.3)**<br>**p = 0.724**	**0.1 (-0.1, 0.3)**<br>**p = 0.467**	
tDCS-active + BrainHQ (N=66)	66<br>2.9 (2.8, 3.1)	54<br>2.3 (2.1, 2.5)	54<br>-0.6 (-0.7, -0.4)	-0.1 (-0.3, 0.1)<br>p = 0.502	0.0 (-0.3, 0.2)<br>p = 0.745	**0.0 (-0.2, 0.2)**<br>**p = 0.963**
tDCS-sham + BrainHQ (N=65)	64<br>2.7 (2.5, 2.9)	58<br>2.2 (2.0, 2.4)	58<br>-0.5 (-0.7, -0.4)			

[1] mECog2 has a score from 1 to 5, with 5 being worst.
[2] Negative adjusted difference in mean change indicates the treatment group in the row did better than the treatment group in the column.

© 2025 Knopman DS et al. *JAMA Neurology*.

eTable 8. Postexertional Malaise Across Treatment Groups

	All Participants* (N=320)	Active Comparator (N=63)	BrainHQ (N=66)	PASC-CoRE + BrainHQ (N=66)	tDCS- active + BrainHQ (N=62)	tDCS- sham + BrainHQ (N=63)
Baseline	214/283 (75.6%)	43/56 (76.8%)	41/59 (69.5%)	43/54 (79.6%)	43/54 (79.6%)	44/60 (73.3%)
Middle of Intervention	167/259 (64.5%)	30/53 (56.6%)	37/55 (67.3%)	39/53 (73.6%)	28/46 (60.9%)	33/52 (63.5%)
End of Intervention	143/256 (55.9%)	26/54 (48.1%)	32/56 (57.1%)	31/50 (62.0%)	25/46 (54.3%)	29/50 (58.0%)
End of Study	102/182 (56.0%)	20/40 (50.0%)	18/34 (52.9%)	26/36 (72.2%)	14/34 (41.2%)	24/38 (63.2%)

* Missing values were a result of a procedural workflow flaw not related to individual patient characteristics.

© 2025 Knopman DS et al. *JAMA Neurology*.

eTable 9. Treatment-Emergent Serious Adverse Events

	All Participants (N=320) n (%)[1] nAE	Active Comparator (N=63) n (%)[1] nAE	BrainHQ (N=66) n (%)[1] nAE	PASC-CoRE + BrainHQ (N=66) n (%)[1] nAE	tDCS-active + BrainHQ (N=62) n (%)[1] nAE	tDCS-sham + BrainHQ (N=63) n (%)[1] nAE
Any serious adverse event[2]	11 (3.4%) 18	3 (4.8%) 7	2 (3.0%) 2	3 (4.5%) 5	0	3 (4.8%) 4
Atrial fibrillation	1 (0.3%) 1	0	0	0	0	1 (1.6%) 1
Autonomic nervous system imbalance	1 (0.3%) 1	0	0	0	0	1 (1.6%) 1
Bacteremia	1 (0.3%) 1	0	0	1 (1.5%) 1	0	0
Breast cancer	1 (0.3%) 1	0	0	0	0	1 (1.6%) 1
Bronchial secretion retention	1 (0.3%) 1	1 (1.6%) 1	0	0	0	0
COVID-19	1 (0.3%) 1	1 (1.6%) 1	0	0	0	0
Cerebral aneurysm perforation	1 (0.3%) 1	0	1 (1.5%) 1	0	0	0
Concussion	1 (0.3%) 1	0	0	1 (1.5%) 1	0	0
Dehydration	1 (0.3%) 1	1 (1.6%) 1	0	0	0	0
Gastrointestinal viral infection	1 (0.3%) 1	0	1 (1.5%) 1	0	0	0
Hypersensitivity	1 (0.3%) 1	0	0	1 (1.5%) 1	0	0
Hypertransaminasaemia	1 (0.3%) 1	1 (1.6%) 1	0	0	0	0
Pancreatitis	1 (0.3%) 2	0	0	1 (1.5%) 2	0	0
Pneumonia	1 (0.3%) 1	1 (1.6%) 1	0	0	0	0
Post-acute COVID-19 syndrome	1 (0.3%) 1	1 (1.6%) 1	0	0	0	0
Pyelonephritis	1 (0.3%) 1	0	0	0	0	1 (1.6%) 1
Sepsis	1 (0.3%) 1	1 (1.6%) 1	0	0	0	0

Abbreviation: nAE, number of adverse events.
1. Number and percentage of participants with an emergent serious adverse event.
2. An event is presented in this table if the start date is on the same day of or after the start of randomized treatment. One subject received an animal bite the day before starting randomized treatment. The animal bite became infected and met serious adverse event criteria after starting randomized treatment. As a result of the event start date being before randomized treatment start date, this event is not included in the summary above.

© 2025 Knopman DS et al. *JAMA Neurology*.

eFigure 1. Distribution of mECog2 Scores: ITT Population

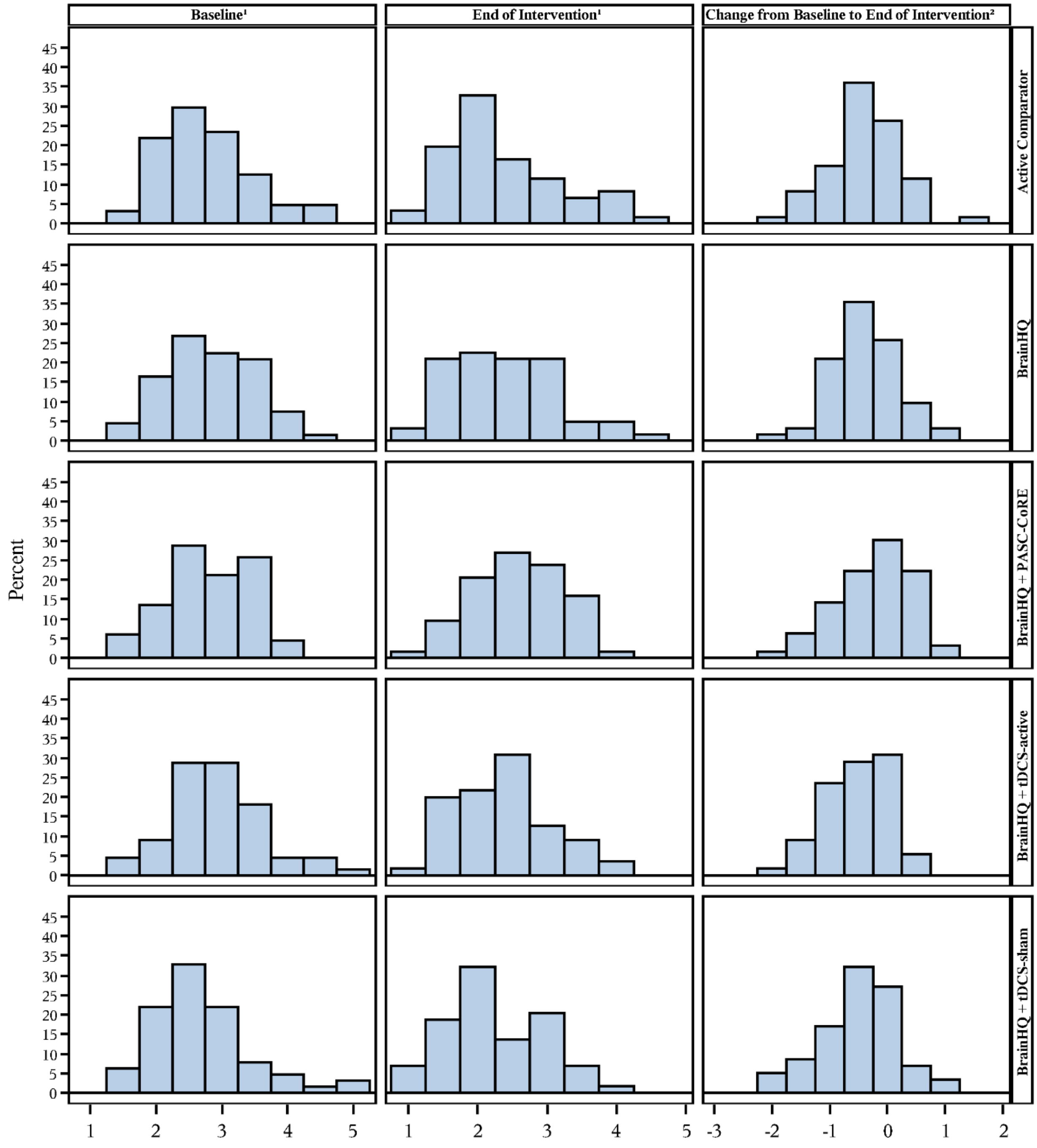

1. mECog2 has a score from 1 to 5, with 5 being worst.
2. Negative values indicate improvement when interpreting Change from Baseline to End of Intervention.

© 2025 Knopman DS et al. *JAMA Neurology*.

eFigure 2. Subgroup Analysis Forest Plots: ITT Population

A. BrainHQ vs active comparator

Group	Interaction P-Value	N	Adj. Diff. In Mean Change	95% CI
All subjects		300	0.0	-0.20, 0.20
Baseline ECog2	0.922			
Impaired		110	0.0	-0.35, 0.33
Unimpaired		190	0.0	-0.28, 0.23
Sex	0.423			
Male		78	0.2	-0.27, 0.58
Female		222	0.0	-0.27, 0.18
Baseline Fatigue	0.897			
Fatigue		163	0.0	-0.31, 0.24
No Fatigue		137	0.0	-0.29, 0.28
Baseline Sleep Disorder	0.412			
Sleep Disorder		123	-0.1	-0.47, 0.22
No Sleep Disorder		176	0.1	-0.20, 0.31
Baseline Anxiety	0.869			
Anxiety		122	0.0	-0.28, 0.32
No Anxiety		178	0.0	-0.28, 0.25
Baseline Depression	0.689			
Depression		60	0.1	-0.38, 0.54
No Depression		240	0.0	-0.25, 0.20
Baseline Post Exertional Malaise (PEM)	0.679			
PEM		201	0.1	-0.19, 0.30
No PEM		67	0.0	-0.46, 0.37

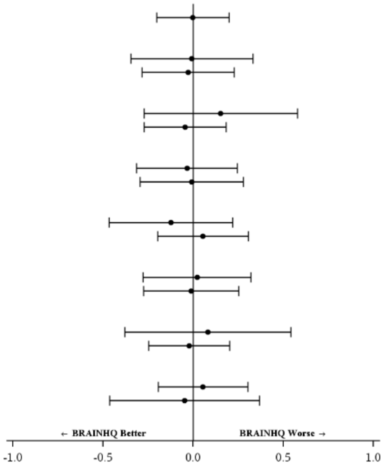

B. PASC-CoRE + BrainHQ vs active comparator

Group	Interaction P-Value	N	Adj. Diff. In Mean Change	95% CI
All subjects		300	0.1	-0.06, 0.34
Baseline ECog2	0.048			
Impaired		110	-0.1	-0.45, 0.21
Unimpaired		190	0.3	0.04, 0.56
Sex	0.530			
Male		78	0.3	-0.17, 0.69
Female		222	0.1	-0.12, 0.33
Baseline Fatigue	0.118			
Fatigue		163	0.0	-0.30, 0.25
No Fatigue		137	0.3	-0.00, 0.59
Baseline Sleep Disorder	0.727			
Sleep Disorder		123	0.1	-0.26, 0.40
No Sleep Disorder		176	0.1	-0.11, 0.40
Baseline Anxiety	0.833			
Anxiety		122	0.1	-0.19, 0.41
No Anxiety		178	0.1	-0.11, 0.41
Baseline Depression	0.468			
Depression		60	0.3	-0.16, 0.73
No Depression		240	0.1	-0.13, 0.33
Baseline Post Exertional Malaise (PEM)	0.795			
PEM		201	0.1	-0.12, 0.38
No PEM		67	0.1	-0.42, 0.54

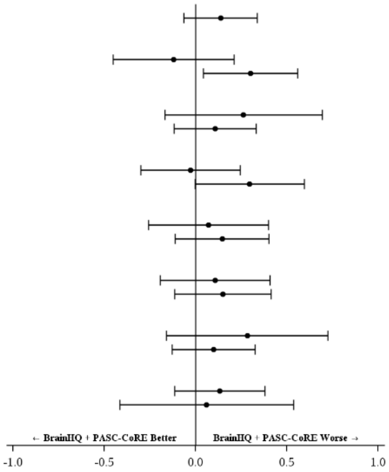

© 2025 Knopman DS et al. *JAMA Neurology*.

C. tDCS Active + BrainHQ vs tDCS Sham + BrainHQ

Group	Interaction P-Value	N	Adj. Diff. In Mean Change	95% CI
All subjects		300	0.0	-0.20, 0.21
Baseline ECog2	0.090			
Impaired		110	0.3	-0.08, 0.65
Unimpaired		190	-0.1	-0.37, 0.16
Sex	0.006			
Male		78	0.5	0.07, 0.83
Female		222	-0.2	-0.43, 0.06
Baseline Fatigue	0.242			
Fatigue		163	0.1	-0.16, 0.40
No Fatigue		137	-0.1	-0.43, 0.18
Baseline Sleep Disorder	0.635			
Sleep Disorder		123	0.1	-0.24, 0.37
No Sleep Disorder		176	0.0	-0.33, 0.25
Baseline Anxiety	0.014			
Anxiety		122	0.3	0.01, 0.68
No Anxiety		178	-0.2	-0.44, 0.08
Baseline Depression	0.536			
Depression		60	0.1	-0.33, 0.62
No Depression		240	0.0	-0.26, 0.21
Baseline Post Exertional Malaise (PEM)	0.335			
PEM		201	0.0	-0.26, 0.24
No PEM		67	0.2	-0.20, 0.69

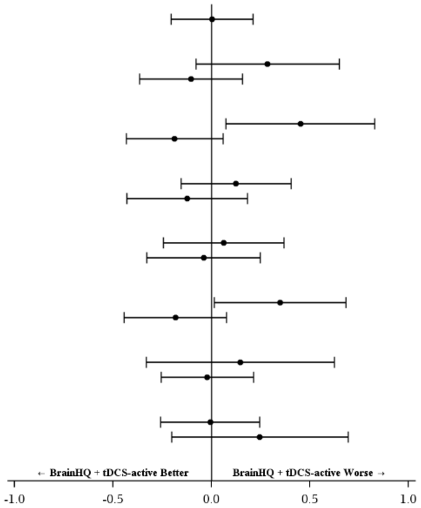

D. PASC-CoRE + BrainHQ vs BrainHQ

Group	Interaction P-Value	N	Adj. Diff. In Mean Change	95% CI
All subjects		300	0.1	-0.06, 0.34
Baseline ECog2	0.037			
Impaired		110	-0.1	-0.43, 0.21
Unimpaired		190	0.3	0.07, 0.59
Sex	0.856			
Male		78	0.1	-0.27, 0.49
Female		222	0.2	-0.08, 0.38
Baseline Fatigue	0.148			
Fatigue		163	0.0	-0.25, 0.27
No Fatigue		137	0.3	-0.00, 0.61
Baseline Sleep Disorder	0.618			
Sleep Disorder		123	0.2	-0.12, 0.51
No Sleep Disorder		176	0.1	-0.17, 0.35
Baseline Anxiety	0.708			
Anxiety		122	0.1	-0.23, 0.40
No Anxiety		178	0.2	-0.09, 0.41
Baseline Depression	0.761			
Depression		60	0.2	-0.26, 0.66
No Depression		240	0.1	-0.10, 0.34
Baseline Post Exertional Malaise (PEM)	0.905			
PEM		201	0.1	-0.17, 0.33
No PEM		67	0.1	-0.33, 0.54

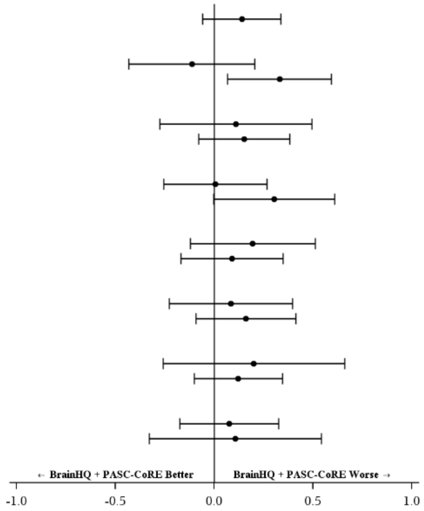

© 2025 Knopman DS et al. *JAMA Neurology*.
