# Supplementary material for: Evaluation of Interventions for Cognitive Symptoms in Long COVID: A Randomized Clinical Trial
Source: JAMA Neurol. 2025 Nov 10;83(1):49–59. doi: 10.1001/jamaneurol.2025.4415 (PMC12603944; doi:10.1001/jamaneurol.2025.4415)
Supplement: Supplement 3. — Nonauthor Collaborators. RECOVER-NEURO Clinical Trial Group [file jamaneurol-e254415-s003.pdf]

Supplemental Online Content: Nonauthor Collaborators
*First name, last name, and suffix (if applicable) are required and will appear in PubMed.

*Group Name(s): RECOVER-NEURO Clinical Trial Group							
*First Name and Middle Initial(s)	*Last Name	*Suffix (eg, Jr, III)	Academic Degrees	Institution	Location (city, state/province, country)	Role or Contribution, eg, chair, principal investigator	Group (if more than 1 Group listed in the byline) and/or Subgroup (eg, Steering Committee)
Andrew	Lawler			University of Colorado	Aurora, CO	Legal Contact	
Brandon	Johnson			University of Colorado	Aurora, CO	Co-Coordinator	
Chloe	Pitsch			University of Colorado	Aurora, CO	Co-Coordinator	
Elen	Feuerriegel			University of Colorado	Aurora, CO	Research Manager	
Jeff	McKeehan			University of Colorado	Aurora, CO	Azure DCRI Data Portal Contact; Financial Contact; Regulatory Contact; Study Coordinator	
Joie	Molden			University of Colorado	Aurora, CO	Rater	
Kayleigh	Reid			University of Colorado	Aurora, CO	Co-Coordinator	
Kristine	Erlandson			University of Colorado	Aurora, CO	Sub-Investigator	
Sarah	Dowling			University of Colorado	Aurora, CO	Azure DCRI Data Portal Contact; Co-Coordinator	
Thomas	Wodushek			University of Colorado	Aurora, CO	Rater	
Diara	Canton			Beth Israel Deaconess Medical Center	Boston, MA	Co-Coordinator	
Dilara	Turut			Beth Israel Deaconess Medical Center	Boston, MA	Co-Coordinator	
Ethan	Sheppard			Beth Israel Deaconess Medical Center	Boston, MA	Co-Coordinator	
Jennifer	Scott-Sutherland			Beth Israel Deaconess Medical Center	Boston, MA	Rater	
Kristine	Hauser			Beth Israel Deaconess Medical Center	Boston, MA	Sub-Investigator; Nurse	
Lynn	Shaughnessy			Beth Israel Deaconess Medical Center	Boston, MA	Rater	
Michael	Doyle			Beth Israel Deaconess Medical Center	Boston, MA	Azure DCRI Data Portal Contact; Study Coordinator	
Michelle	Beck			Beth Israel Deaconess Medical Center	Boston, MA	Financial Contact; Regulatory Contact; Study Coordinator	
Sabrina	Heisey			Beth Israel Deaconess Medical Center	Boston, MA	Legal Contact	

Supplemental Online Content: Nonauthor Collaborators
*First name, last name, and suffix (if applicable) are required and will appear in PubMed.

***First Name and Middle Initial(s)**	***Last Name**	***Suffix (eg, Jr, III)**	Academic Degrees	Institution	Location (city, state/province, country)	Role or Contribution, eg, chair, principal investigator	Group (if more than 1 Group listed in the byline) and/or Subgroup (eg, Steering Committee)
Vaishanvi	Katragadda			Beth Israel Deaconess Medical Center	Boston, MA	Co-Coordinator	
Amidat	Yusuff			Boston Medical Center	Boston, MA	Lab Contact	
Amy	Perlack			Boston Medical Center	Boston, MA	Legal Contact	
Anh	Tran			Boston Medical Center	Boston, MA	Nurse	
Brigid	Dwyer			Boston Medical Center	Boston, MA	Financial Contact; Regulatory Contact; Study Coordinator	
Caroline	Altaras			Boston Medical Center	Boston, MA	Rater	
Christie	Merisme			Boston Medical Center	Boston, MA	Lab Contact	
David	Krause			Boston Medical Center	Boston, MA	Study Coordinator	
Duncan	Schulte			Boston Medical Center	Boston, MA	Azure DCRI Data Portal Contact; Regulatory Contact; Study Coordinator	
Fabiola	Dupre			Boston Medical Center	Boston, MA	Lab Contact	
Gina	Sanchez			Boston Medical Center	Boston, MA	Study Coordinator	
Maxine	Krengel			Boston Medical Center	Boston, MA	Rater	
Monica	Ly			Boston Medical Center	Boston, MA	Rater	
Olanike	Asupoto			Boston Medical Center	Boston, MA	Study Coordinator	
Quinneil	Simmons			Boston Medical Center	Boston, MA	Financial Contact; Research Manager	
Ryan	Schroeder			Boston Medical Center	Boston, MA	Regulatory Contact; Research Director	
Shannon	Timlin			Boston Medical Center	Boston, MA	Other	
Ycar	Devis			Boston Medical Center	Boston, MA	Lab Contact	
Della	Carter			Boston University School of Medicine	Boston, MA	Lab Contact	
Leomaris	Caceres			NYU Langone Health	Brooklyn, NY	Co-Coordinator	
Miguel	Rodriguez			NYU Langone Health	Brooklyn, NY	Co-Coordinator	
Zariya	Alvarez			NYU Langone Health	Brooklyn, NY	Study Coordinator	
Amin	Yakubov			NYU Langone Health - Brooklyn	Brooklyn, NY	Co-Coordinator	

Supplemental Online Content: Nonauthor Collaborators
*First name, last name, and suffix (if applicable) are required and will appear in PubMed.

*First Name and Middle Initial(s)	*Last Name	*Suffix (eg, Jr, III)	Academic Degrees	Institution	Location (city, state/province, country)	Role or Contribution, eg, chair, principal investigator	Group (if more than 1 Group listed in the byline) and/or Subgroup (eg, Steering Committee)
Celia	Engelson			NYU Langone Health - Brooklyn	Brooklyn, NY	Sub-Investigator	
Michael	Reyes			NYU Langone Health - Brooklyn	Brooklyn, NY	Co-Coordinator	
Alvin	Youkhana			Rush University Medical Center	Chicago, IL	Research Assistant	
Cheryl	Jennings			Rush University Medical Center	Chicago, IL	Lab Contact	
David	Gonzalez			Rush University Medical Center	Chicago, IL	Rater	
Dina	Naquiallah			Rush University Medical Center	Chicago, IL	Azure DCRI Data Portal Contact; Study Coordinator	
Dylan	Behun			Rush University Medical Center	Chicago, IL	Azure DCRI Data Portal Contact; Co-Coordinator	
Ellen	Stephen			Rush University Medical Center	Chicago, IL	Sub-Investigator	
Emily	Timm			Rush University Medical Center	Chicago, IL	Rater	
Farrah	Bilimoria			Rush University Medical Center	Chicago, IL	Lab Contact	
Grace	Li			Rush University Medical Center	Chicago, IL	Financial Contact; Legal Contact; Legal Contact	
Jakob	Stricker			Rush University Medical Center	Chicago, IL	Lab Contact	
Jimena	Torres			Rush University Medical Center	Chicago, IL	Lab Contact	
Maria	Garcia			Rush University Medical Center	Chicago, IL	Co-Coordinator; Research Assistant	
Michelle	Thomas			Rush University Medical Center	Chicago, IL	Financial Contact; Legal Contact; Regulatory Contact	
Minnie	Kang			Rush University Medical Center	Chicago, IL	Azure DCRI Data Portal Contact; Co-Coordinator	
Rebeca	Suarez			Rush University Medical Center	Chicago, IL	Co-Coordinator	
Tania	Carmona			Rush University Medical Center	Chicago, IL	Co-Coordinator	
Ana	Ramirez			University of Illinois at Chicago	Chicago, IL	Study Coordinator	
Grace	Kadubek			University of Illinois at Chicago	Chicago, IL	Co-Coordinator	
Aileen	Baker			University of Illinois, Breathe Chicago Center	Chicago, IL	Administrative Assistant	

Supplemental Online Content: Nonauthor Collaborators
*First name, last name, and suffix (if applicable) are required and will appear in PubMed.

***First Name and Middle Initial(s)**	***Last Name**	***Suffix (eg, Jr, III)**	Academic Degrees	Institution	Location (city, state/province, country)	Role or Contribution, eg, chair, principal investigator	Group (if more than 1 Group listed in the byline) and/or Subgroup (eg, Steering Committee)
Andrea	Romo-Serna			University of Illinois, Breathe Chicago Center	Chicago, IL	Co-Coordinator	
Barbara	Predki			University of Illinois, Breathe Chicago Center	Chicago, IL	Co-Coordinator	
Cindy	Leman			University of Illinois, Breathe Chicago Center	Chicago, IL	Financial Contact; Legal Contact	
Cristina	Panhans			University of Illinois, Breathe Chicago Center	Chicago, IL	Research Manager	
Genevieve	Roth			University of Illinois, Breathe Chicago Center	Chicago, IL	Rater	
Gowrisree	Rudraraju			University of Illinois, Breathe Chicago Center	Chicago, IL	Co-Coordinator	
Hannah	Miller			University of Illinois, Breathe Chicago Center	Chicago, IL	Co-Coordinator	
Julie	DeLisa			University of Illinois, Breathe Chicago Center	Chicago, IL	Regulatory Contact	
Kat	Williams			University of Illinois, Breathe Chicago Center	Chicago, IL	Co-Coordinator	
Kimberly	Woodson			University of Illinois, Breathe Chicago Center	Chicago, IL	Research Manager	
Kyle	Jennette			University of Illinois, Breathe Chicago Center	Chicago, IL	Sub-Investigator; Rater	
Laura	Carrera			University of Illinois, Breathe Chicago Center	Chicago, IL	Co-Coordinator	
Laura	Villanueva			University of Illinois, Breathe Chicago Center	Chicago, IL	Co-Coordinator	
Maria	Norwick			University of Illinois, Breathe Chicago Center	Chicago, IL	Co-Coordinator	
Mariam	Martinez			University of Illinois, Breathe Chicago Center	Chicago, IL	Co-Coordinator	
Matt	Rowley			University of Illinois, Breathe Chicago Center	Chicago, IL	Lab Contact	

Supplemental Online Content: Nonauthor Collaborators
*First name, last name, and suffix (if applicable) are required and will appear in PubMed.

*First Name and Middle Initial(s)	*Last Name	*Suffix (eg, Jr, III)	Academic Degrees	Institution	Location (city, state/province, country)	Role or Contribution, eg, chair, principal investigator	Group (if more than 1 Group listed in the byline) and/or Subgroup (eg, Steering Committee)
Melissa	Rutherford			University of Illinois, Breathe Chicago Center	Chicago, IL	Co-Coordinator	
Michael	Carrithers			University of Illinois, Breathe Chicago Center	Chicago, IL	Sub-Investigator	
Neil	Pliskin			University of Illinois, Breathe Chicago Center	Chicago, IL	Sub-Investigator	
Nicolas	Perez			University of Illinois, Breathe Chicago Center	Chicago, IL	Lab Contact	
Nicole	Durkin			University of Illinois, Breathe Chicago Center	Chicago, IL	Rater	
Nikita	Maniar			University of Illinois, Breathe Chicago Center	Chicago, IL	Research Director; Sub-Investigator	
Rachel	Beety			University of Illinois, Breathe Chicago Center	Chicago, IL	Co-Coordinator	
Raktima	Daspgupta			University of Illinois, Breathe Chicago Center	Chicago, IL	Co-Coordinator	
Ramaswamy	Ramchandran			University of Illinois, Breathe Chicago Center	Chicago, IL	Lab Contact	
Ruthie	Jaramillo			University of Illinois, Breathe Chicago Center	Chicago, IL	Rater	
Sharon	Hasek			University of Illinois, Breathe Chicago Center	Chicago, IL	Co-Coordinator	
Susy	Macias			University of Illinois, Breathe Chicago Center	Chicago, IL	Co-Coordinator	
Tina	Schuh			University of Illinois, Breathe Chicago Center	Chicago, IL	Regulatory Contact; Study Coordinator	
Sarah	Ialacci			University Hospitals of Cleveland	Cleveland, OH	Financial Contact; Legal Contact	
Christopher	Bailey			University Hospitals of Cleveland	Cleveland, OH	Rater	
Danielle	Labbato			University Hospitals of Cleveland	Cleveland, OH	Co-Coordinator	
Amanda	Hautmann			University Hospitals of Cleveland	Cleveland, OH	Rater	
Beth	Smith			University Hospitals of Cleveland	Cleveland, OH	Lab Contact	

Supplemental Online Content: Nonauthor Collaborators
*First name, last name, and suffix (if applicable) are required and will appear in PubMed.

***First Name and Middle Initial(s)**	***Last Name**	***Suffix (eg, Jr, III)**	Academic Degrees	Institution	Location (city, state/province, country)	Role or Contribution, eg, chair, principal investigator	Group (if more than 1 Group listed in the byline) and/or Subgroup (eg, Steering Committee)
Dia	Nath			University Hospitals of Cleveland	Cleveland, OH	Rater	
Jessica	Meyer			University Hospitals of Cleveland	Cleveland, OH	Rater	
Kathryn	DiFrancesco			University Hospitals of Cleveland	Cleveland, OH	Regulatory Contact	
Kevin	Walkup			University Hospitals of Cleveland	Cleveland, OH	Rater	
Kimberly	Pettinato			University Hospitals of Cleveland	Cleveland, OH	Co-Coordinator; Study Coordinator	
Lynette	Curtis			University Hospitals of Cleveland	Cleveland, OH	Azure DCRI Data Portal Contact; Co-Coordinator	
Joviane	Daher			University Hospitals of Cleveland	Cleveland, OH	Co-Coordinator	
Sarah	Scott			University Hospitals of Cleveland	Cleveland, OH	Lab Contact	
Ziad	Koberssy			University Hospitals of Cleveland	Cleveland, OH	Co-Coordinator	
Caleb	Mavar			University Hospitals of Cleveland	Cleveland, OH	Study Coordinator	
John	Andrefsky			University Hospitals of Cleveland	Cleveland, OH	Sub-Investigator	
Alexander	Merheb			University Hospitals of Cleveland	Cleveland, OH	Co-Coordinator	
Carla	Hernandez			University Hospitals of Cleveland	Cleveland, OH	Co-Coordinator; Research Manager	
Jhony	Baissary			University Hospitals of Cleveland	Cleveland, OH	Azure DCRI Data Portal Contact; Study Coordinator	
Ornina	Atieh			University Hospitals of Cleveland	Cleveland, OH	Co-Coordinator; Azure DCRI Data Portal Contact	
Carol	Pereira			Duke Clinical Research Institute	Durham, NC		
Deborah	Carpenter			Duke Clinical Research Institute	Durham, NC		
Jennifer	Raynor			Duke Clinical Research Institute	Durham, NC		
Kristine	Brunton			Duke Clinical Research Institute	Durham, NC		
Andreea	Podgoreanu			Duke Clinical Research Institute	Durham, NC		
Pyper	Bunch			Duke Clinical Research Institute	Durham, NC		
Richard	Brown			Duke Clinical Research Institute	Durham, NC		
Sean	O'Brien			Duke Clinical Research Institute	Durham, NC		
Sheila	Dickey			Duke Clinical Research Institute	Durham, NC		
Steven	McNulty			Duke Clinical Research Institute	Durham, NC		

Supplemental Online Content: Nonauthor Collaborators
*First name, last name, and suffix (if applicable) are required and will appear in PubMed.

***First Name and Middle Initial(s)**	***Last Name**	***Suffix (eg, Jr, III)**	Academic Degrees	Institution	Location (city, state/province, country)	Role or Contribution, eg, chair, principal investigator	Group (if more than 1 Group listed in the byline) and/or Subgroup (eg, Steering Committee)
Varsha	Gajjar			Duke Clinical Research Institute	Durham, NC		
Alec	Neale			NorthShore Medical Group	Evanston, IL	Rater	
Cari	Cohen			NorthShore Medical Group	Evanston, IL	Rater	
Carolina	Escobar			NorthShore Medical Group	Evanston, IL	Study Coordinator	
Elizabeth	Geary			NorthShore Medical Group	Evanston, IL	Rater	
Franco	Campanella			NorthShore Medical Group	Evanston, IL	Sub-Investigator	
Kirsten	Anderson			NorthShore Medical Group	Evanston, IL	Co-Coordinator	
Laura	Prestige			NorthShore Medical Group	Evanston, IL	Sub-Investigator	
Leslie	Guidotti-Breting			NorthShore Medical Group	Evanston, IL	Rater	
Megan	Biewer			NorthShore Medical Group	Evanston, IL	Co-Coordinator	
Melissa	Morton			NorthShore Medical Group	Evanston, IL	Nurse	
Nicholas	Mathenia			NorthShore Medical Group	Evanston, IL	Sub-Investigator	
Oluwadamilola	Adeyemi			NorthShore Medical Group	Evanston, IL	Sub-Investigator	
Samanike	Hengst			NorthShore Medical Group	Evanston, IL	Co-Coordinator	
Wei Ning	Chi			NorthShore Medical Group	Evanston, IL	Co-Coordinator	
Adam	Vanderloo			NorthShore UHS Research Institute	Evanston, IL	Financial Contact	
Amanda	Caplan			NorthShore University Health System	Evanston, IL	Sub-Investigator	
July	Chen			NorthShore University Health System	Evanston, IL	Study Coordinator	
Mary Ellen	Acree			NorthShore University Health System	Evanston, IL	Sub-Investigator	
Michael	Glickman			NorthShore University Health System	Evanston, IL	Sub-Investigator	
Mukund	Nakhate			NorthShore University Health System	Evanston, IL	Study Coordinator	
Zaw Nu	Dan			NorthShore University Health System	Evanston, IL	Co-Coordinator	
Phyliis	Switzer			KU Alzheimer’s Disease Research Center	Fairway, KS	Rater	
Shambhu	Aryal			Inova Fairfax Hospital	Falls Church, VA	Co-Coordinator	
Jessica	Sanchez			University of Texas Medical School	Houston, TX	Co-Coordinator	
Luke	Sims			University of Texas Medical School	Houston, TX	Co-Coordinator	
Rebecca	Ferdinand			McGovern Medical School at UTHealth	Houston, TX	Co-Coordinator; Rater	
Mehriban	Mammadova			MHH-TMC	Houston, TX	Regulatory Contact; Study Coordinator	
Carolyn	Grimes			University of Texas - Houston Medical School	Houston, TX	Research Manager	

*First name, last name, and suffix (if applicable) are required and will appear in PubMed.

***First Name and Middle Initial(s)**	***Last Name**	***Suffix (eg, Jr, III)**	Academic Degrees	Institution	Location (city, state/province, country)	Role or Contribution, eg, chair, principal investigator	Group (if more than 1 Group listed in the byline) and/or Subgroup (eg, Steering Committee)
Elizabeth	Vidales			University of Texas - Houston Medical School	Houston, TX	Co-Coordinator	
Hoai Phuang	Pham			University of Texas - Houston Medical School	Houston, TX	Research Assistant	
John	Lopez			University of Texas - Houston Medical School	Houston, TX	Rater	
Laura	Nielsen			University of Texas - Houston Medical School	Houston, TX	Co-Coordinator	
Maryjane	Keller			University of Texas - Houston Medical School	Houston, TX	Co-Coordinator	
Syeda	Uruba			University of Texas - Houston Medical School	Houston, TX	Co-Coordinator	
Maria	Hernandez			University of Texas Health Science Center	Houston, TX	Lab Contact	
Bela	Patel			University of Texas Health Science Center - Houston	Houston, TX	Sub-Investigator	
Matthew	Lin			University of Texas Health Science Center - Houston	Houston, TX	Sub-Investigator	
Virginia	Umana-Sanchez			University of Texas Health Science Center - Houston	Houston, TX	Azure DCRI Data Portal Contact; Study Coordinator	
Chong	Pak			University of Texas Health Sciences Center at Houston	Houston, TX	Legal Contact	
Chong	Pak			University of Texas Health Sciences Center at Houston	Houston, TX	Financial Contact	
Jeffery	Gainer			Jacksonville Aging Studies Center	Jacksonville, FL	Co-Coordinator	
Renae	Burr			Jacksonville Aging Studies Center	Jacksonville, FL	Co-Coordinator	
Ashleigh	Trapuzzano			University of Florida Jacksonville	Jacksonville, FL	Regulatory Contact; Azure DCRI Data Portal Contact	
Brian	Celso			University of Florida Jacksonville	Jacksonville, FL	Rater	

Supplemental Online Content: Nonauthor Collaborators
*First name, last name, and suffix (if applicable) are required and will appear in PubMed.

*First Name and Middle Initial(s)	*Last Name	*Suffix (eg, Jr, III)	Academic Degrees	Institution	Location (city, state/province, country)	Role or Contribution, eg, chair, principal investigator	Group (if more than 1 Group listed in the byline) and/or Subgroup (eg, Steering Committee)
Grace	Bienkowski			University of Florida Jacksonville	Jacksonville, FL	Azure DCRI Data Portal Contact; Study Coordinator	
Jennifer	Bowman			University of Florida Jacksonville	Jacksonville, FL	Financial Contact; Legal Contact; Research Manager	
Jorge	Verdecia			University of Florida Jacksonville	Jacksonville, FL	Sub-Investigator	
Kathryn	Zerwekh			University of Florida Jacksonville	Jacksonville, FL	Regulatory Contact	
Liliana	Serrano			University of Florida Jacksonville	Jacksonville, FL	Research Director	
Meghan	Marotz			University of Florida Jacksonville	Jacksonville, FL	Co-Coordinator	
Piper	Propp			University of Florida Jacksonville	Jacksonville, FL	Co-Coordinator	
Yonatan	Sarig			University of Florida Jacksonville	Jacksonville, FL	Rater	
Annie	Murphy			University of Kansas Medical Center	Kansas City, KS	Rater	
Brandon	Comfort			University of Kansas Medical Center	Kansas City, KS	Sub-Investigator	
Gina	Brown			University of Kansas Medical Center	Kansas City, KS	Legal Contact	
Iri	Torres			University of Kansas Medical Center	Kansas City, KS	Rater	
Jeffrey	Klein			University of Kansas Medical Center	Kansas City, KS	Sub-Investigator	
Jessica	Reed			University of Kansas Medical Center	Kansas City, KS	Azure DCRI Data Portal Contact; Study Coordinator	
John	Walker			University of Kansas Medical Center	Kansas City, KS	Rater	
John	Moore			University of Kansas Medical Center	Kansas City, KS	Co-Coordinator; Study Coordinator	
Lisa	Tempest-Browning			University of Kansas Medical Center	Kansas City, KS	Regulatory Contact	
Mudassar	Arain			University of Kansas Medical Center	Kansas City, KS	Co-Coordinator	
Pamela	Kemp			University of Kansas Medical Center	Kansas City, KS	Rater	
Samantha	Sullivan			University of Kansas Medical Center	Kansas City, KS	Rater	
Sean	Obrien			University of Kansas Medical Center	Kansas City, KS	Co-Coordinator; Study Coordinator	
Sonja	Way			University of Kansas Medical Center	Kansas City, KS	Financial Contact	
Jessica	Chevalier			Dartmouth Hitchcock Medical Center	Lebanon, NH	Financial Contact; Legal Contact	
Carly	Sykes			Dartmouth Hitchcock Medical Center	Lebanon, NH	Other	

Supplemental Online Content: Nonauthor Collaborators
*First name, last name, and suffix (if applicable) are required and will appear in PubMed.

***First Name and Middle Initial(s)**	***Last Name**	***Suffix (eg, Jr, III)**	Academic Degrees	Institution	Location (city, state/province, country)	Role or Contribution, eg, chair, principal investigator	Group (if more than 1 Group listed in the byline) and/or Subgroup (eg, Steering Committee)
Carrie	Kruck			Dartmouth Hitchcock Medical Center	Lebanon, NH	Rater	
Elaine	Kiriakopoulos			Dartmouth Hitchcock Medical Center	Lebanon, NH	Sub-Investigator	
Lisa	Sackett			Dartmouth Hitchcock Medical Center	Lebanon, NH	Rater	
Sarah	Kaden			Dartmouth Hitchcock Medical Center	Lebanon, NH	Rater	
Claire	Bang			Dartmouth-Hitchcock Medical Center	Lebanon, NH	Azure DCRI Data Portal Contact; Regulatory Contact; Study Coordinator	
Mary	Hynes			Dartmouth-Hitchcock Medical Center	Lebanon, NH	Nurse	
Torrey	Gallagher			Dartmouth-Hitchcock Medical Center	Lebanon, NH	Lab Contact	
Margaret	Childers-Kakos			West Virginia Clinical	Morgantown, WV	Regulatory Contact	
Ahmad	Zia			West Virginia Clinical and Translational Science Institute	Morgantown, WV	Co-Coordinator	
Amy	South			West Virginia Clinical and Translational Science Institute	Morgantown, WV	Co-Coordinator	
Anne	Oravets			West Virginia Clinical and Translational Science Institute	Morgantown, WV	Other	
Carolyn	Ferreyra			West Virginia Clinical and Translational Science Institute	Morgantown, WV	Rater	
Chad	Glaze			West Virginia Clinical and Translational Science Institute	Morgantown, WV	Research Manager	
Daphne-Dominique	Villanueva			West Virginia Clinical and Translational Science Institute	Morgantown, WV	Sub-Investigator	
Erica	Blystone			West Virginia Clinical and Translational Science Institute	Morgantown, WV	Azure DCRI Data Portal Contact; Study Coordinator	
Jane	Pritchard			West Virginia Clinical and Translational Science Institute	Morgantown, WV	Financial Contact; Legal Contact	
Laura	Alyward			West Virginia Clinical and Translational Science Institute	Morgantown, WV	Rater	
Melissa	Cooper			West Virginia Clinical and Translational Science Institute	Morgantown, WV	Co-Coordinator	

Supplemental Online Content: Nonauthor Collaborators
*First name, last name, and suffix (if applicable) are required and will appear in PubMed.

*First Name and Middle Initial(s)	*Last Name	*Suffix (eg, Jr, III)	Academic Degrees	Institution	Location (city, state/province, country)	Role or Contribution, eg, chair, principal investigator	Group (if more than 1 Group listed in the byline) and/or Subgroup (eg, Steering Committee)
Michelle	Chidester			West Virginia Clinical and Translational Science Institute	Morgantown, WV	Regulatory Contact	
Rachael	Alderson			West Virginia Clinical and Translational Science Institute	Morgantown, WV	Azure DCRI Data Portal Contact; Co-Coordinator	
Renee	Clark			West Virginia Clinical and Translational Science Institute	Morgantown, WV	Other	
Sally	Hodder			West Virginia Clinical and Translational Science Institute	Morgantown, WV	Co-Coordinator; Sub-Investigator	
Arif	Sarwari			West Virginia University	Morgantown, WV	Sub-Investigator	
James	Bardes			West Virginia University	Morgantown, WV	Sub-Investigator	
Joy	Juskowich			West Virginia University	Morgantown, WV	Sub-Investigator	
Shelly	Welch			WVU Biomedical Research Center	Morgantown, WV	Research Director	
Alyssa	Civil			Icahn School of Medicine at Mount Sinai	New York, NY	Research Manager	
Ashley	Bendl			Icahn School of Medicine at Mount Sinai	New York, NY	Co-Coordinator	
Elisa	Dicker			Icahn School of Medicine at Mount Sinai	New York, NY	Co-Coordinator	
Hannah	Hanson			Icahn School of Medicine at Mount Sinai	New York, NY	Azure DCRI Data Portal Contact; Co-Coordinator	
Heather	Viola			Icahn School of Medicine at Mount Sinai	New York, NY	Sub-Investigator	
Janice	Morinigo			Icahn School of Medicine at Mount Sinai	New York, NY	Lab Contact	
Karisma	Pantaleon			Icahn School of Medicine at Mount Sinai	New York, NY	Rater	
Kathryn	Marcon			Icahn School of Medicine at Mount Sinai	New York, NY	Azure DCRI Data Portal Contact; Financial Contact; Regulatory Contact; Regulatory Contact; Research Manager; Study Coordinator	
Kimberly	Stone			Icahn School of Medicine at Mount Sinai	New York, NY	Data Management Contact	

Supplemental Online Content: Nonauthor Collaborators
*First name, last name, and suffix (if applicable) are required and will appear in PubMed.

***First Name and Middle Initial(s)**	***Last Name**	***Suffix (eg, Jr, III)**	Academic Degrees	Institution	Location (city, state/province, country)	Role or Contribution, eg, chair, principal investigator	Group (if more than 1 Group listed in the byline) and/or Subgroup (eg, Steering Committee)
Kiwan	Stewart			Icahn School of Medicine at Mount Sinai	New York, NY	Study Coordinator	
Lorraine	Evo-Ortega			Icahn School of Medicine at Mount Sinai	New York, NY	Co-Coordinator	
Maria	Loizos			Icahn School of Medicine at Mount Sinai	New York, NY	Sub-Investigator	
Megan	McVeety			Icahn School of Medicine at Mount Sinai	New York, NY	Study Coordinator	
Sabina	Guliyeva			Icahn School of Medicine at Mount Sinai	New York, NY	Research Manager	
Alyssa	Civil			Icahn School of Medicine at Mount Sinai	New York, NY		
Eric	Watson			NYU Langone Health	New York, NY		
Shayna	Pehel			NYU Langone Health	New York, NY		
Tim	Ko			NYU Langone Health	New York, NY		
Leah	Caropolo			NYULH Office of Science and Research	New York, NY	Financial Contact; Legal Contact	
Huriye Deniz	Kocas			NYU Lagone Medical Center	New York, NY	Rater	
Luis	Fernandez			NYU Lagone Medical Center	New York, NY	Rater	
Andrew	ODonnell			Stanford University	Palo Alto, CA	Co-Coordinator	
Corey	Saperia			Stanford University	Palo Alto, CA	Sub-Investigator	
Daniel	Thai			Stanford University	Palo Alto, CA	Azure DCRI Data Portal Contact	
Divya	Pathak			Stanford University	Palo Alto, CA	Research Manager	
Jennifer	Keller			Stanford University	Palo Alto, CA	Rater	
Kelly	Olszewski			Stanford University	Palo Alto, CA	Co-Coordinator	
Kimberly	Clinton			Stanford University	Palo Alto, CA	Nurse	
Kirsten	Cherian			Stanford University	Palo Alto, CA	Rater	
Layton	Dutton			Stanford University	Palo Alto, CA	Legal Contact	
Ma	Elepano			Stanford University	Palo Alto, CA	Nurse	
Rebecca	Lin			Stanford University	Palo Alto, CA	Nurse	
Sukanya	Mohapatra			Stanford University	Palo Alto, CA	Azure DCRI Data Portal Contact; Co-Coordinator	
Ting	Chen			Stanford University	Palo Alto, CA	Nurse	
Tonita	Wroolie			Stanford University	Palo Alto, CA	Rater	
Zenita	Leang			Stanford University	Palo Alto, CA	Co-Coordinator	
Sumana	Shashidhar			Stanford University School of Medicine	Palo Alto, CA	Research Manager	
Tiffany	Nguyen			Stanford University School of Medicine	Palo Alto, CA	Lab Contact	

*First name, last name, and suffix (if applicable) are required and will appear in PubMed.

*First Name and Middle Initial(s)	*Last Name	*Suffix (eg, Jr, III)	Academic Degrees	Institution	Location (city, state/province, country)	Role or Contribution, eg, chair, principal investigator	Group (if more than 1 Group listed in the byline) and/or Subgroup (eg, Steering Committee)
Ashwath	Ravisankar			OSF Saint Francis Medical Center	Peoria, IL	Study Coordinator	
Brycie	Weiden			OSF Saint Francis Medical Center	Peoria, IL	Rater	
Daniel	Brown			OSF Saint Francis Medical Center	Peoria, IL	Rater	
Dawn	Bolliger			OSF Saint Francis Medical Center	Peoria, IL	Co-Coordinator	
Heather	Glesne			OSF Saint Francis Medical Center	Peoria, IL	Financial Contact; Legal Contact	
Jennifer	Dixon			OSF Saint Francis Medical Center	Peoria, IL	Regulatory Contact; Study Coordinator	
Praneeth	Chebrolu			OSF Saint Francis Medical Center	Peoria, IL	Research Director	
Zarine	Gazali			OSF Saint Francis Medical Center	Peoria, IL	Co-Coordinator	
Whitney	Hurst			OSF St. Francis Medical Center	Peoria, IL	Study Coordinator	
Avery	Fry			Banner University Medical Center	Phoenix, AZ	Lab Contact	
Ganesh	Murthy			Banner University Medical Center	Phoenix, AZ	Sub-Investigator	
Garrett	Grischo			Banner University Medical Center	Phoenix, AZ	Lab Contact	
Heidi	Erickson			Banner University Medical Center	Phoenix, AZ	Azure DCRI Data Portal Contact; Study Coordinator; Regulatory Contact	
Isaias	Gomez			Banner University Medical Center	Phoenix, AZ	Azure DCRI Data Portal Contact; Study Coordinator	
Jacquelynn	Copeland			Banner University Medical Center	Phoenix, AZ	Rater	
Judy	Galaz			Banner University Medical Center	Phoenix, AZ	Azure DCRI Data Portal Contact; Co-Coordinator	
Lynn	Autry			Banner University Medical Center	Phoenix, AZ	Rater	
Marjorie	Dilise-Russo			Banner University Medical Center	Phoenix, AZ	Rater	
Michelle	James			Banner University Medical Center	Phoenix, AZ	Rater	
Mrinalini	Kala			Banner University Medical Center	Phoenix, AZ	Lab Contact	
Nikki	Marshell			Banner University Medical Center	Phoenix, AZ	Study Coordinator	
Sabine	Borwege			Banner University Medical Center	Phoenix, AZ	Lab Contact	
Sheila	Vadovicky			Banner University Medical Center	Phoenix, AZ	Rater	

Supplemental Online Content: Nonauthor Collaborators
*First name, last name, and suffix (if applicable) are required and will appear in PubMed.

*First Name and Middle Initial(s)	*Last Name	*Suffix (eg, Jr, III)	Academic Degrees	Institution	Location (city, state/province, country)	Role or Contribution, eg, chair, principal investigator	Group (if more than 1 Group listed in the byline) and/or Subgroup (eg, Steering Committee)
Stephanie	Marsh			Univ of Arizona College of Medicine Phoenix	Phoenix, AZ	Study Coordinator	
William	Hartley			University of Arizona	Phoenix, AZ	Study Coordinator	
Linda	Murtagh			Stanford University	Redwood, CA	Financial Contact	
Anna	Kennedy			University Of Utah	Salt Lake City, UT	Rater	
Annie	Meltzer-Christensen			University Of Utah	Salt Lake City, UT	Financial Contact	
Caitlyn	Stringham			University Of Utah	Salt Lake City, UT	Rater	
Ella	Gaskin			University Of Utah	Salt Lake City, UT	Azure DCRI Data Portal Contact; Co-Coordinator	
Melissa	Judd			University Of Utah	Salt Lake City, UT	Azure DCRI Data Portal Contact; Regulatory Contact; Study Coordinator	
Ryan	Smith			University Of Utah	Salt Lake City, UT	Co-Coordinator	
Sarah	Cote			University Of Utah	Salt Lake City, UT	Rater	
Shreya	Mahasenan			University Of Utah	Salt Lake City, UT	Azure DCRI Data Portal Contact; Study Coordinator	
Todd	Bjorklund			University Of Utah	Salt Lake City, UT	Legal Contact	
Cheryl	Farner			Medical Arts and Research Center	San Antonio, TX	Other	
Barbara	Taylor			The University of Texas Health Science Center at San Antonio	San Antonio, TX	Sub-Investigator	
Bridgette	Soileau			The University of Texas Health Science Center at San Antonio	San Antonio, TX	Sub-Investigator	
Cyrena	Cote			The University of Texas Health Science Center at San Antonio	San Antonio, TX	Study Coordinator	
Gabrielle	Hromas			The University of Texas Health Science Center at San Antonio	San Antonio, TX	Rater	

Supplemental Online Content: Nonauthor Collaborators
*First name, last name, and suffix (if applicable) are required and will appear in PubMed.

***First Name and Middle Initial(s)**	***Last Name**	***Suffix (eg, Jr, III)**	Academic Degrees	Institution	Location (city, state/province, country)	Role or Contribution, eg, chair, principal investigator	Group (if more than 1 Group listed in the byline) and/or Subgroup (eg, Steering Committee)
Irma	Scholler			The University of Texas Health Science Center at San Antonio	San Antonio, TX	Azure DCRI Data Portal Contact; Regulatory Contact; Study Coordinator	
Lisa	Longoria			The University of Texas Health Science Center at San Antonio	San Antonio, TX	Co-Coordinator	
Patricia	Heard			The University of Texas Health Science Center at San Antonio	San Antonio, TX	Co-Coordinator	
Hillary	Johnson			University of Texas Health Science Center	San Antonio, TX	Administrative Assistant	
Johnnie	Jones			University of Texas Health Science Center	San Antonio, TX	Administrative Assistant; Financial Contact	
Robin	Tragus			University of Texas Health Science Center	San Antonio, TX	Study Coordinator	
Maya	Harris			University of Texas Health Science Center at San Antonio	San Antonio, TX	Co-Coordinator	
Alexis	Pinones			University of Texas Health Sciences Center at San Antonio	San Antonio, TX	Lab Director	
Alyssa	Anderson			University of Texas Health Sciences Center at San Antonio	San Antonio, TX	Lab Contact	
Gabrielyd	Hastings			University of Texas Health Sciences Center at San Antonio	San Antonio, TX	Co-Coordinator	
jessica	Courtright			University of Texas Health Sciences Center at San Antonio	San Antonio, TX	Co-Coordinator	
Jessica	Hernandez			University of Texas Health Sciences Center at San Antonio	San Antonio, TX	Co-Coordinator	
Madhumita	Jayakumar			University of Texas Health Sciences Center at San Antonio	San Antonio, TX	Co-Coordinator; Lab Contact	
Melinda	Fischer			University of Texas Health Sciences Center at San Antonio	San Antonio, TX	Azure DCRI Data Portal Contact; Study Coordinator	

Supplemental Online Content: Nonauthor Collaborators
*First name, last name, and suffix (if applicable) are required and will appear in PubMed.

***First Name and Middle Initial(s)**	***Last Name**	***Suffix (eg, Jr, III)**	Academic Degrees	Institution	Location (city, state/province, country)	Role or Contribution, eg, chair, principal investigator	Group (if more than 1 Group listed in the byline) and/or Subgroup (eg, Steering Committee)
Michael	Sullivan			University of Texas Health Sciences Center at San Antonio	San Antonio, TX	Co-Coordinator	
Pamela	Solis			University of Texas Health Sciences Center at San Antonio	San Antonio, TX	Research Assistant	
Alex	Harteloo			University of Washington	Seattle, WA	Co-Coordinator	
Anna	Elias-Warren			University of Washington	Seattle, WA	Study Coordinator; Azure DCRI Data Portal Contact	
Daniel	Nguyen			University of Washington	Seattle, WA	Co-Coordinator	
Dylan	McDonald			University of Washington	Seattle, WA	Regulatory Contact; Study Coordinator	
Helen	Nguyen			University of Washington	Seattle, WA	Azure DCRI Data Portal Contact; Co-Coordinator	
Holly	Rau			University of Washington	Seattle, WA	Rater	
Jean	Mernaugh			University of Washington	Seattle, WA	Co-Coordinator	
Jennifer	Logue			University of Washington	Seattle, WA	Azure DCRI Data Portal Contact; Financial Contact; Legal Contact; Research Manager	
Kathleen	Pagulayan			University of Washington	Seattle, WA	Sub-Investigator	
Kino	Watanabe			University of Washington	Seattle, WA	Co-Coordinator	
Kristen	Huden			University of Washington	Seattle, WA	Co-Coordinator	
Laurel	Peabody			University of Washington	Seattle, WA	Rater	
Tiffany	Mei			University of Washington	Seattle, WA	Study Coordinator; Azure DCRI Data Portal Contact	
Catherine	Ogunwuyi			Adventist Healthcare White Oak Medical Center	Silver Spring, MD	Co-Coordinator	

Supplemental Online Content: Nonauthor Collaborators
*First name, last name, and suffix (if applicable) are required and will appear in PubMed.

***First Name and Middle Initial(s)**	***Last Name**	***Suffix (eg, Jr, III)**	Academic Degrees	Institution	Location (city, state/province, country)	Role or Contribution, eg, chair, principal investigator	Group (if more than 1 Group listed in the byline) and/or Subgroup (eg, Steering Committee)
Esther	Cobbina			Jadestone Clinical Research, LLC	Silver Spring, MD	Azure DCRI Data Portal Contact; Co-Coordinator	
Ian	Sankar			Jadestone Clinical Research, LLC	Silver Spring, MD	Azure DCRI Data Portal Contact; Regulatory Contact	
Lisa	Nguyen			Jadestone Clinical Research, LLC	Silver Spring, MD	Co-Coordinator; Study Coordinator	
Nicole	Fromm			Jadestone Clinical Research, LLC	Silver Spring, MD	Rater	
Rowan	Arnold			Jadestone Clinical Research, LLC	Silver Spring, MD	Azure DCRI Data Portal Contact; Co-Coordinator; Study Coordinator	
Stephanie	Ogu			Jadestone Clinical Research, LLC	Silver Spring, MD	Co-Coordinator	
Ying	Yuan			Jadestone Clinical Research, LLC	Silver Spring, MD	Co-Coordinator; Financial Contact; Legal Contact; Research Manager	
Homam	Ibrahim			White Oak Medical Center	Silver Spring, MD	Sub-Investigator	
Izabella	Szum			NorthShore Medical Group	Skokie, IL	Nurse	
Jignesh	Patel			NorthShore Medical Group	Skokie, IL	Study Coordinator	
Sonia	Sultan			NorthShore Medical Group	Skokie, IL	Co-Coordinator	
Yasmin	Jazayeri			Stanford University School of Medicine	Stanford, CA	Study Coordinator	
Timothy	Hatlen			Division of Infectious Diseases Harbor University of California at Los Angeles Medical Center	Torrance, CA	Sub-Investigator	
Betty	Anderson			Lundquist Institute at Harbor-UCLA Medical Center	Torrance, CA	Study Coordinator	
Isabel	Munoz			Lundquist Institute at Harbor-UCLA Medical Center	Torrance, CA	Co-Coordinator	
Logan	Rhys			Lundquist Institute at Harbor-UCLA Medical Center	Torrance, CA	Rater	

Supplemental Online Content: Nonauthor Collaborators
*First name, last name, and suffix (if applicable) are required and will appear in PubMed.

***First Name and Middle Initial(s)**	***Last Name**	***Suffix (eg, Jr, III)**	Academic Degrees	Institution	Location (city, state/province, country)	Role or Contribution, eg, chair, principal investigator	Group (if more than 1 Group listed in the byline) and/or Subgroup (eg, Steering Committee)
Matthew	Wright			Lundquist Institute at Harbor-UCLA Medical Center	Torrance, CA	Rater; Sub-Investigator	
Ryan	Vane			Lundquist Institute at Harbor-UCLA Medical Center	Torrance, CA	Rater	
Jessica	Kim			Lundquist Institute for Biomedical Innovation at Harbor-UCLA Medical Center	Torrance, CA	Legal Contact	
Leticia	Diaz			Lundquist Institute for Biomedical Innovation at Harbor-UCLA Medical Center	Torrance, CA	Co-Coordinator	
Nathan	Yee			Lundquist Institute for Biomedical Innovation at Harbor-UCLA Medical Center	Torrance, CA	Sub-Investigator	
Robert	Gruhn			Lundquist Institute for Biomedical Innovation at Harbor-UCLA Medical Center	Torrance, CA	Regulatory Contact	
Sarah	Browngoetz			Lundquist Institute for Biomedical Innovation at Harbor-UCLA Medical Center	Torrance, CA	Financial Contact; Legal Contact	
Thomas	Decato			Lundquist Institute for Biomedical Innovation at Harbor-UCLA Medical Center	Torrance, CA	Sub-Investigator	
Elisha	Johnson			University of Arizona	Tucson, AZ	Financial Contact; Legal Contact	
Arista	Chand			University of Arizona - Banner Medical Center	Tucson, AZ	Sub-Investigator	
Ernesto	Ortiz			University of Arizona - Banner Medical Center	Tucson, AZ	Co-Coordinator	
Heidi	Erickson			University of Arizona - Banner Medical Center	Tucson, AZ	Azure DCRI Data Portal Contact; Study Coordinator; Regulatory Contact	
Ken	Bottrill			University of Arizona - Banner Medical Center	Tucson, AZ	Rater	
Kymberly	Henderson			University of Arizona - Banner Medical Center	Tucson, AZ	Rater	
Lindsey	Hildebrand			University of Arizona - Banner Medical Center	Tucson, AZ	Rater	

Supplemental Online Content: Nonauthor Collaborators
*First name, last name, and suffix (if applicable) are required and will appear in PubMed.

***First Name and Middle Initial(s)**	***Last Name**	***Suffix (eg, Jr, III)**	Academic Degrees	Institution	Location (city, state/province, country)	Role or Contribution, eg, chair, principal investigator	Group (if more than 1 Group listed in the byline) and/or Subgroup (eg, Steering Committee)
Marketta	Johnson			University of Arizona - Banner Medical Center	Tucson, AZ	Lab Contact	
Richard	DeArmond			University of Arizona - Banner Medical Center	Tucson, AZ	Lab Contact	
Trina	Hughes			University of Arizona - Banner Medical Center	Tucson, AZ	Azure DCRI Data Portal Contact; Study Coordinator	
