# Supplementary material for: Evaluation of Interventions for Cognitive Symptoms in Long COVID: A Randomized Clinical Trial
Source: JAMA Neurol. 2025 Nov 10;83(1):49–59. doi: 10.1001/jamaneurol.2025.4415 (PMC12603944; doi:10.1001/jamaneurol.2025.4415)
Supplement: Supplement 4. — Data Sharing Statement. [file jamaneurol-e254415-s004.pdf]

Data Sharing Statement

Knopman DS, et al; RECOVER-NEURO Clinical Trial Group. Evaluation of interventions for cognitive symptoms in long COVID. *JAMA Neurol.* Published online November 10, 2025. doi: 10.1001/jamaneurol.2025.4415

Data
Additional Information: ClinicalTrials.gov Identifier: NCT05965739
Data available: Yes
Data types: Deidentified participant data
How to access data: All study data will be posted on BioData Catalyst website at the time that this article is published
When available: With publication

Supporting Documents
Document types: Statistical/analytic code, Informed consent form
How to access documents: Bio Data catalyst
When available: With publication

Additional Information
Who can access the data: responsible researchers
Types of analyses: any valid purpose
Mechanisms of data availability: through RECOVER trial leadership
